# Learning the PTM code through a coarse-to-fine mechanism-aware framework

**DOI:** 10.1038/s41467-026-73148-3

**Published:** 2026-05-15

**Authors:** Jingjie Zhang, Hanqun Cao, Zijun Gao, Yu Wang, Shaoning Li, Jun Xu, Cheng Tan, Jun Zhu, Chang-Yu Hsieh, Chunbin Gu, Pheng Ann Heng

**Affiliations:** 1 Department of Computer Science and Engineering, The Chinese University of Hong Kong, Hong Kong, China; 2 School of Computer Science, Peking University, Beijing, China; 3 Institute of Reproductive Medicine, Medical School, Nantong University, Nantong, China; 4 School of Life Sciences, Tsinghua University, Beijing, China; 5 College of Pharmaceutical Sciences, Zhejiang University, Hangzhou, China

**Keywords:** Machine learning, Protein function predictions

## Abstract

Post-translational modifications (PTMs) form a complex combinatorial “code” that orchestrates protein function and cellular signaling. However, deciphering this code by predicting PTM sites and linking sites to their regulatory enzymes remains a fundamental challenge. Here, we present COMPASS-PTM, a mechanism-aware, coarse-to-fine learning framework that unifies residue-level multi-label PTM prediction with enzyme-substrate assignment by jointly modeling PTM patterns and their catalytic regulators. COMPASS-PTM builds upon protein language models, integrating physicochemical descriptors and a crosstalk-aware prompting mechanism to learn biologically coherent patterns of cooperative and antagonistic modifications, while addressing the dual long-tail distribution inherent in PTM data. Across multiple proteome-scale benchmarks, COMPASS-PTM improves over the strongest evaluated baselines, with a 122% relative improvement in F1-score for multi-label site prediction and a 54% gain in zero-shot enzyme assignment. Furthermore, the model demonstrates interpretable generalization, recovering canonical kinase motifs and mechanistically linking missense variants to both local PTM disruptions and global rewiring of enzyme-substrate networks. By coupling statistical learning with explicit biochemical knowledge, COMPASS-PTM unifies site-level and enzyme-level prediction into a single framework that learns the grammar underlying protein regulation and signaling.

## Abstract (web summary)

Post-translational modifications regulate protein function but are difficult to decode systematically. Here, the authors present COMPASS-PTM, a coarse-to-fine framework that predicts multi-label PTM sites, assigns regulatory enzymes, and links variants to PTM and network rewiring.

## Introduction

Post-translational modifications (PTMs) constitute a dynamic regulatory layer that orchestrates signal integration, spatiotemporal control, and the functional diversification of proteins^1,2^. This regulation is often achieved through a combinatorial “code”, whereby specific patterns of modifications—governed by a network of crosstalk—collectively dictate a protein’s fate^3–5^. Deciphering this regulatory code is therefore foundational to understanding health and the molecular basis of diseases, such as cancer and neurodegeneration, which are frequently characterized by profound dysregulation of PTM circuitry^6–9^.

However, despite decades of experimental and computational work, the PTM code remains only partially understood, and in particular we lack systematic ways to track how it is rewired by genetic variation. Mass spectrometry-based proteomics has transformed PTM discovery but remains limited by cost, coverage, and context-specificity^10,11^, motivating the development of computational predictors. Historically, most models have adopted a “site-centric” viewpoint: they assign a probability that a given residue can carry a specific PTM based on local sequence motifs. Early neural networks operating on small sequence windows^12–15^, followed by RNNs^16^ and more recently protein language model (PLM)-based architectures^17–20^, have substantially improved sensitivity by leveraging evolutionary and long-range sequence context. Yet even modern PLM-based PTM predictors still tend to score one PTM type at a time and treat each site as an independent decision, providing little insight into how multiple PTM types cooperate or compete on the same substrate, or how enzyme specificity and genetic variation shape these patterns.

Recent multi-label frameworks and large-scale PTM models have begun to relax the one-site/one-PTM assumption, allowing several modifications to co-localize on a residue and extending the predictive context to the whole protein^21,22^. For example, MIND-S^22^ expands the predictive context from local windows to a broader protein-level schema using Graph Neural Networks (GNNs). However, three fundamental challenges remain. First, most methods lack explicit biochemical constraints to encode rules of crosstalk—such as when phosphorylation facilitates ubiquitination, or acetylation competes with methylation on lysine—so they struggle in co-modified contexts where conditional dependence is central to function^5^. Second, PTM data exhibit a dual long-tail distribution: (i) across PTM types, a small number of common modifications account for most labeled instances, whereas many biologically important PTMs are rare^23^; and (ii) across sequence positions, positive modification sites are sparse relative to the overwhelming background of unmodified residues. Together, these two coupled long-tail effects induce severe imbalance at both the class and instance levels. Third, even when enzyme information or PTM networks are considered, site-level PTM prediction and enzyme assignment are often modeled separately, with no shared representation bridging the two tasks. Most crucially, the prediction task is usually truncated at site profiling: models infer “where” a modification may occur but not “who” writes or erases it, leaving PTM predictions disconnected from the network of enzymes that implement the regulatory code. Even recent variant-centric PTM models, such as DeepMVP^16^, primarily quantify how mutations perturb local PTM propensities, without linking these changes to specific enzymes or to the rewiring of upstream regulatory circuits.

**Fig. 1:**
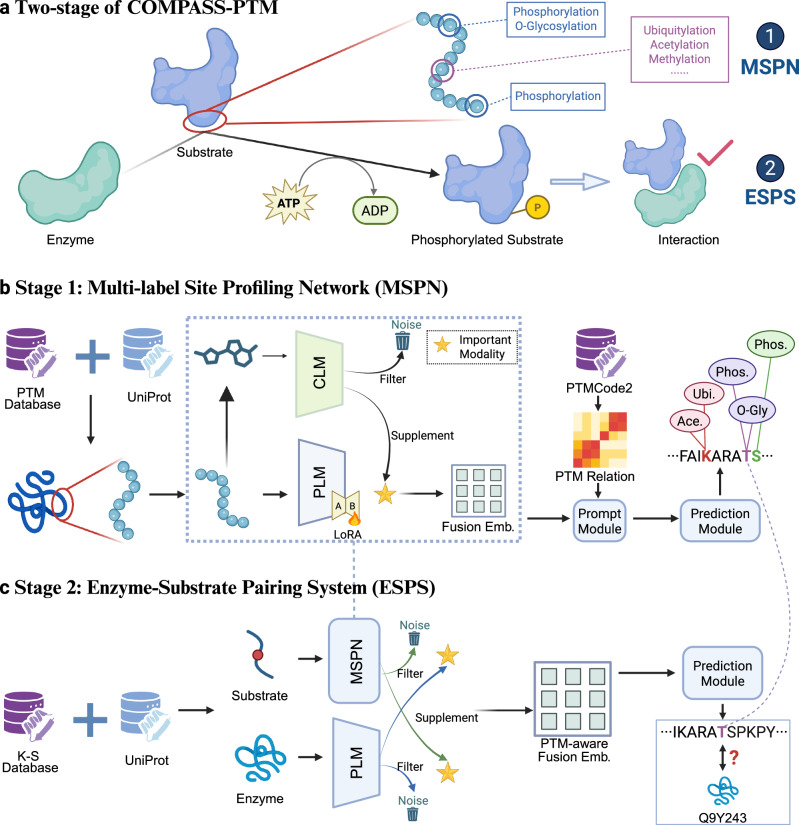
The COMPASS-PTM framework for mechanism-aware PTM prediction. **a** Conceptual overview of the two-stage, coarse-to-fine framework. Stage 1, the Multi-label Site Profiling Network (MSPN), performs proteome-scale prediction to identify potential PTM sites and their modification types. Stage 2, the Enzyme-Substrate Pairing System (ESPS), takes high-confidence sites from Stage 1 and predicts their cognate enzymes, providing mechanistic context. The yellow circle labeled “P” denotes a phosphate group. **b** Architecture of the Stage 1 MSPN. A dual-modal encoder fuses representations from a protein language model (PLM), efficiently adapted via low-rank adaptation (LoRA)^53^, and a chemical language model (CLM). A crosstalk-aware prompting module uses a PTM relationship matrix, initialized from co-occurrence statistics in the PTMCode2 database^5^, as a learnable inductive bias to guide prediction of interdependent PTM patterns. The heatmap indicates PTM relationship strength. The yellow star denotes retained informative features, the trash-bin icon denotes filtered noise, and colored circles/labels denote different PTM types. **c**, Architecture of the Stage 2 ESPS. For a given substrate-enzyme pair, the system integrates the PTM-aware substrate embedding generated by MSPN with an enzyme embedding to predict their interaction probability, thereby linking a modification site to its regulator. K-S database denotes the enzyme-substrate relationship database used in Stage 2. Emb. denotes embedding. Created in BioRender. Cao, H. (2026) https://BioRender.com/ax2kzid.

To move beyond isolated site scoring, we reframe PTM inference as decoding a regulatory program that links sequence, combinatorial modification patterns, and upstream enzymes. In this view, PTM sites and enzyme assignments are not two unrelated tasks but two resolutions of the same underlying regulatory code: a coarse, substrate-centric description of multi-PTM potential and a fine-grained, enzyme-resolved description of the enzyme-substrate relationships that realize that potential, which we model jointly rather than in isolation. Guided by this view, we introduce COMPASS-PTM (Coarse-to-fine Oriented Multi-label PTM Site Prediction And enzyme-Substrate aSsignment), a two-stage, mechanism-aware (enzyme-resolved specificity-aware modeling) framework that learns a single, unified PTM-aware representation bridging both levels of inference, as shown in Fig. 1a. In Stage 1, the Multi-label Site Profiling Network (MSPN) fine-tunes a PLM and combines its sequence representations with physicochemical descriptors through a dual-modal encoder, producing residue-level embeddings that capture both evolutionary constraints and chemical reactivity. A crosstalk-aware prompting module injects priors on PTM co-occurrence as a learnable attention bias, steering the model toward biologically plausible multi-PTM configurations (Fig. 1b), while a hybrid loss specifically counteracts the dual long-tail distribution. This yields a PTM program for each protein, represented as a structured multi-label profile of plausible residue–modification assignments under realistic class imbalance. In Stage 2, an Enzyme-Substrate Pairing System (ESPS) reuses this PTM-aware substrate representation as its starting point and learns enzyme embeddings for multiple classes of PTM regulators. Rather than acting as an independent classifier, ESPS asks which enzymes best explain the PTM patterns encoded by MSPN for a given enzyme-mediated substrate. In this way, Stage 2 refines the coarse PTM program produced by Stage 1 into an enzyme-resolved regulatory map that ties specific enzyme-mediated modification sites and patterns to the annotated enzymes that write or erase them, without discarding the information learned at the site level.

In this work, we show that this coarse-to-fine paradigm yields a synergistic effect across both levels of inference. Stage 1 improves performance on multiple multi-label site benchmarks by up to 122% in relative F1-score, whereas Stage 2 surpasses the previous best method on the DARKIN zero-shot kinase assignment benchmark by 54%^24^, indicating that a single PTM-aware representation supports both site and enzyme prediction. Beyond these quantitative gains, COMPASS-PTM recovers canonical, family-specific kinase motifs and organizes enzymes and substrates into biochemically coherent manifolds. Because both stages share the same PTM-aware representation, the framework also links missense variants to changes in local PTM probabilities, the gain or loss of specific enzyme-substrate interactions, and the rewiring of pathway-level regulatory programs. We further demonstrate this variant → PTM → enzyme trajectory in downstream applications, from proteome-wide screens for PTM-disrupting mutations to mechanistic case studies that recapitulate known pathogenic signaling pathways and propose enzyme-rewiring hypotheses for uncharacterized variants. Collectively, COMPASS-PTM illustrates a general, enzyme-resolved paradigm for decoding regulatory codes from sequence and translating PTM predictions into experimentally testable models of signaling and disease, one that we anticipate will extend beyond PTMs to enzymatic cascades and protein-protein interaction networks.

## Results

### Overview

PTMs represent a fundamental layer of cellular regulation, yet systematic mapping of these modifications and their regulatory enzymes remains a significant challenge. To address this gap, we developed COMPASS-PTM, a deep learning framework that resolves PTM regulatory networks through a coarse-to-fine, enzyme-aware learning approach, as shown in Fig. 1a. The framework employs a two-stage architecture that first profiles PTM sites at residue resolution and then assigns plausible regulatory enzymes to candidate sites, yielding enzyme-resolved regulatory hypotheses.

The first stage, the Multi-label Site Profiling Network (MSPN), performs proteome-scale PTM site detection, as shown in Fig. 1b. It employs a dual-modal fusion architecture integrating evolutionary sequence information from a Protein Language Model (PLM) with physicochemical reactivity determinants from a Chemical Language Model (CLM). Here, PLM features serve as the primary source of information, while CLM features act as auxiliary inputs. The fusion model preserves critical chemical knowledge while filtering out potential noise arising from erroneous predictions in the chemical modality. This multi-dimensional representation simultaneously captures residue conservation patterns and chemical reactivity principles. A key innovation, crosstalk-aware prompting, introduces biological interdependence directly into the model’s attention mechanism via a learnable prior matrix initialized with empirical PTM co-occurrence statistics^5^. This enables coherent multi-label predictions—for example, increasing confidence in a ubiquitination signal when adjacent phosphorylation is detected—thus echoing known crosstalk mechanisms. To address the dual long-tail distribution inherent in PTM datasets, MSPN employs a hybrid loss function that prioritizes rare but biologically critical classes while focusing learning on challenging individual instances.

The second stage, the Enzyme-Substrate Pairing System (ESPS), links high-confidence sites from MSPN to their regulatory enzymes. ESPS integrates local substrate sequence motifs with global enzyme embedding representations to capture recognition specificity. In this fusion, both substrate and enzyme features are treated as primary sources of information, retaining key characteristics, such as domain architecture while filtering out redundancy and noise from excessively long enzyme sequences. The model directly predicts enzyme-substrate relationships, translating statistical predictions into mechanistic insights, as shown in Fig. 1c. This pipeline produces experimentally testable hypotheses that can elucidate the precise architecture of PTM regulatory networks.

The COMPASS-PTM architecture moves beyond isolated PTM classification by learning a mechanistic map that connects modification sites to their regulatory enzymes. This provides an experimentally actionable framework to systematically identify key regulatory nodes and potential druggable targets within disease-associated signaling pathways.

### COMPASS-PTM improves PTM site profiling across benchmarks

To rigorously evaluate the performance and generalization capabilities of the first stage MSPN, we curated a suite of three distinct benchmark datasets from established proteomics resources. Our evaluation suite comprises two multi-label benchmarks, dbPTM-ML and qPTM-ML, which we developed from the dbPTM^25^ and qPTM^26^ datasets, respectively, to assess the model on the complex biological reality of sites harboring multiple, co-occurring PTM types. To specifically test the model’s generalization to a different task formulation, we also included the PTMint-MC dataset, a multi-class benchmark where each site is annotated with a single PTM type, developed from PTMint dataset^27^. Across these benchmarks, proteins are processed into PTM-site–anchored segments (≤50 aa) and split at the protein level via MMseqs2 clustering (40% identity) to minimize redundancy and evolutionary leakage. Detailed specifications for each dataset are provided in the Supplementary Information S.3.1.2. And Supplementary Fig. 1 summarizes key dataset statistics for our benchmarks, including PTM-type frequency and site-level imbalance, as well as sequence-level distributions. Critically, all three datasets exhibit the dual long-tail distributions characteristic of real-world proteomic data (Supplementary Fig. 1b, d, f, h, i), providing a stringent and realistic testbed for evaluating proteome-scale prediction models.

We assessed model performance using a suite of metrics, primarily F1-score and the Matthews Correlation Coefficient (MCC), two metrics renowned for their reliability in evaluating classifiers on imbalanced multi-label data. To rigorously evaluate performance across both common and rare PTMs, all metrics were calculated using a macro-averaging approach that prevents class imbalance from skewing the results. Detailed description of these metrics are shown in Supplementary Information S.3.2. We compared MSPN against representative PTM predictors, including SAGEPhos, MusiteDeep, MeToken, and MIND-S. For a fair comparison under the multi-label setting, each baseline was adapted where necessary to match the benchmark label space and then retrained using the same train/validation/test splits and evaluation protocol as our model. Full implementation details and baseline adaptations are provided in Supplementary Information S.3.4.

**Fig. 2:**
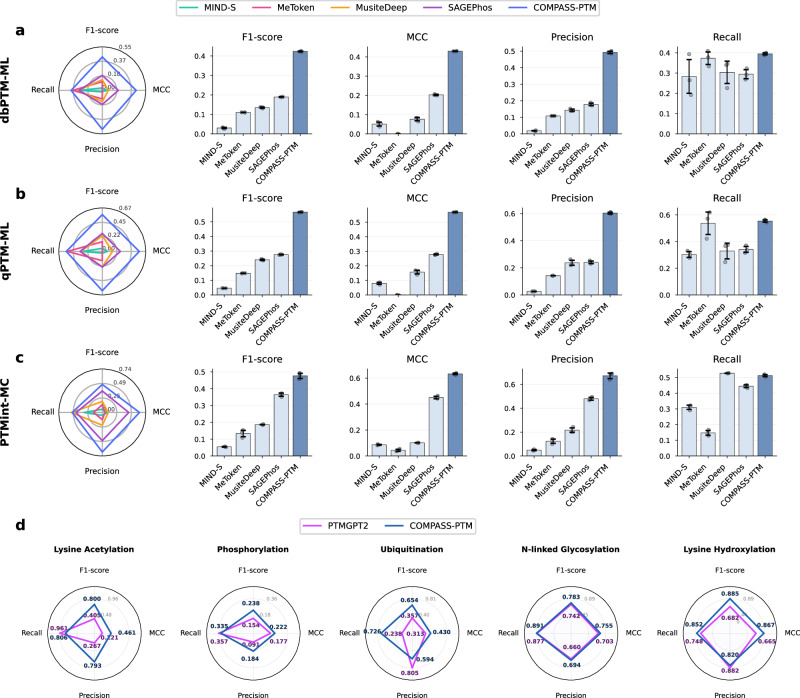
MSPN improves PTM site profiling across benchmark datasets. **a** Performance comparison with four baselines^14,20 --22^ on the dbPTM-ML multi-label benchmark derived from the dbPTM database^25^. **b** Performance comparison on the qPTM-ML multi-label benchmark derived from the qPTM database^26^. **c** Zero-shot generalization performance on the PTMint-MC multi-class benchmark derived from the PTMint database^27^, where models trained on dbPTM-ML were evaluated directly without task-specific fine-tuning. **d** Cross-task generalization performance on five binary classification datasets from the PTMGPT2 study^17^, where MSPN was retrained from scratch on the corresponding dataset without carrying over weights or performing task-specific fine-tuning. For **a**–**c**, the radar charts summarize the same mean metric values shown in the bar plots. Bar heights and radar values indicate mean values across *n* = 3 independent training runs with different random seeds; overlaid dots represent individual runs; error bars indicate s.d. F1, harmonic mean of precision and recall; MCC, Matthews correlation coefficient. Source data are provided as a Source Data file.

MSPN demonstrated strong performance across both large-scale benchmarks, surpassing existing methods. On the dbPTM-ML dataset, as shown in Fig. 2a, MSPN achieved a macro-averaged F1-score of 0.423, representing a 122% improvement over the previous best-performing method, SAGEPhos (0.190). This performance gain was accompanied by a corresponding 111% increase in MCC to 0.429, indicating improved performance across both common and rare modification types. The qPTM-ML benchmark confirmed these results with similar improvements, where MSPN again achieved greater than two-fold enhancement in F1-score compared to competing approaches, alongside a 104% improvement in MCC, as shown in Fig. 2b. These consistent gains across independent datasets underscore the robustness and generalizability of the MSPN architecture, which effectively encodes rich sequence features and models PTM interdependencies to generate biologically meaningful insights.

A critical distinction emerges when examining the underlying drivers of performance improvement. While several existing methods achieve superficially impressive AUROC scores, such performance often reflects high sensitivity at the expense of prohibitive false-positive rates. For instance, MusiteDeep achieves 0.964 AUROC on dbPTM-ML yet produces a precision of only 0.179, rendering its predictions impractical for experimental validation. In contrast, MSPN’s superior F1-score stems from strong precision (0.493 on dbPTM-ML), indicating its capacity to generate high-confidence predictions suitable for experimental follow-up. This high precision is vital for practical utility, as minimizing false positives is essential for guiding limited experimental resources. Therefore, MSPN’s balanced performance delivers reliable, experimentally actionable predictions, marking a significant step forward for the field.

The PTMint-MC evaluation provided a stringent test of MSPN’s discriminative capability across diverse PTM types and generalization performance, assessing the model’s sensitivity to the subtle biochemical signatures that differentiate competing PTM pathways under physiologically relevant but data-constrained scenarios. Despite being optimized for multi-label prediction, MSPN excelled in this multi-class discrimination task, surpassing baseline methods by 30.4% in F1-score and 40.3% in MCC, as shown in Fig. 2c. MSPN’s strong performance on this multi-class task highlights its ability to learn the fundamental, generalizable principles of PTM site recognition.

While residue-wise macro-F1 and MCC quantify per-residue correctness, a “PTM program” interpretation additionally requires that the PTM set assigned at each site remain coherent and restrained. A common multi-label failure mode is over-calling: after a site is localized as modified, the model assigns extra PTM types beyond the curated label set, which can inflate apparent combinatorial PTM patterns and blur protein-level regulatory structure. To quantify this aspect of program-level coherence, we evaluate spurious multi-label burden in both overall and conditional settings, further stratified by all sites versus the single-label subset. We also report the average number of extra labels per spurious residue to capture error severity (details in Supplementary Information S.3.5 and Supplementary Table 1).

Across the multi-label benchmarks, COMPASS-PTM substantially reduces both the frequency and severity of spurious over-calling relative to the strongest baseline, SAGEPhos. In the conditional setting, where at least one true PTM label is already recovered at a residue, the spurious rate decreases from 0.654 to 0.096, indicating that COMPASS-PTM is far less likely to assign additional unsupported PTM types once a site is localized. Moreover, when spurious errors do occur, COMPASS-PTM typically adds only one extra label on average (1.04–1.11), whereas SAGEPhos more often produces multiple extra labels (1.30–1.76). The same pattern holds on the single-label subset, supporting that COMPASS-PTM produces cleaner and more coherent PTM label sets rather than relying on over-expanded multi-label predictions. Consistent with these set-level coherence gains, COMPASS-PTM achieves a mean protein-level event-F1 of 0.6430 on the test set, indicating that the predicted residue–PTM event collection aligns with the ground-truth program at the level of whole proteins.

To rigorously test the generalizability of our architecture’s core principles, we benchmarked MSPN against PTMGPT2, a strong single-PTM predictor, on five of its native binary classification tasks. By retraining our model from scratch for each task—a deliberately challenging setting—we found that MSPN consistently learned more robust representations. This was highlighted by its performance on Lysine Acetylation, where it exceeded the baseline’s F1-Score and MCC by 96.8% and 108.6%, respectively (see Fig. 2d and Supplementary S.3.6 for full results). This superior cross-task performance demonstrates that our architecture effectively learns the intrinsic rules of PTMs rather than task-specific correlations, validating its robustness as a versatile predictive engine.

### COMPASS-PTM achieves robust and generalizable enzyme-substrate pairing

The second stage of our framework, Enzyme–Substrate Pairing System (ESPS), demonstrates strong predictive power on both our curated OmniPath dataset^28^ and the established SAGEPhos benchmark^20^. We evaluate ESPS only on enzyme-mediated PTMs with curated enzyme–substrate annotations: OmniPath spans multiple enzyme-mediated modification classes beyond kinases, whereas SAGEPhos is restricted to kinase–substrate (phosphorylation) relationships.

To assess generalization under realistic deployment scenarios, we evaluate ESPS under three split settings: a warm-start setting where enzymes and substrates may both appear in training and test sets, and two stringent cold-start settings that hold out previously unseen entities, including (i) a substrate cold-start split where all test substrates are excluded from training, and (ii) an enzyme cold-start split where all test enzymes are absent from training. Dataset construction, split protocols, and additional implementation details are provided in Supplementary Information S.3.1.3.

**Fig. 3:**
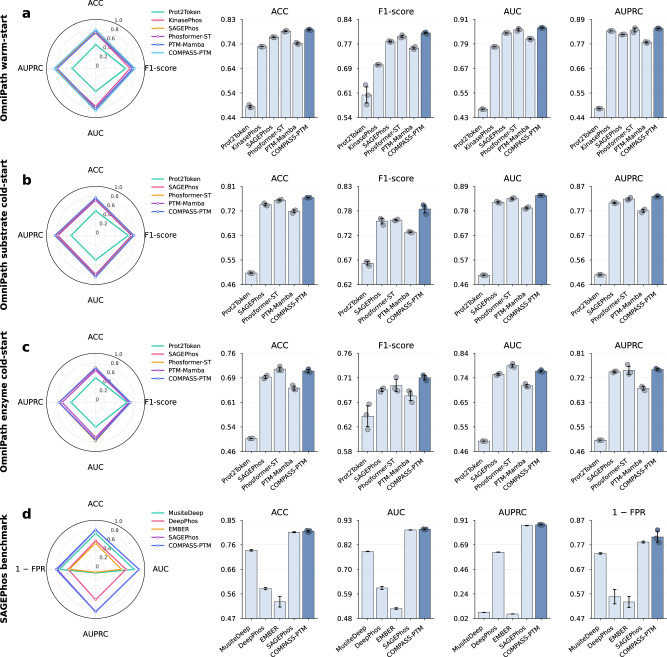
ESPS demonstrates robust performance in enzyme-substrate prediction across diverse benchmarks. **a–c** Performance comparison against competing methods^19,20,29,56,57^ on the OmniPath benchmark^28^ across three data splitting strategies: **a** warm-start; **b** substrate cold-start; and **c** enzyme cold-start. For each panel, the radar chart summarizes the same mean metric values shown in the bar plots. Bar heights and radar values indicate mean metric values across *n* = 3 independent training runs with different random seeds; overlaid dots represent individual independent training runs and error bars indicate s.d. **d** Independent validation on the SAGEPhos benchmark^20^. For COMPASS-PTM, bar heights and radar values indicate mean metric values across *n* = 3 independent training runs with different random seeds, and error bars indicate s.d. For competing methods, the reported values are taken from the SAGEPhos study^20^. ACC, accuracy; AUC, area under the receiver operating characteristic curve; AUPRC, area under the precision-recall curve; FPR, false positive rate. 1 − FPR denotes one minus the false positive rate. Source data are provided as a Source Data file.

On the OmniPath benchmark, we re-trained all relevant baselines under the same protocol to ensure a fair comparison, keeping each baseline’s original architecture unchanged. The only exception is PTM-Mamba^19^: rather than re-implementing an alternative ESPS pipeline, we performed a controlled substitution by replacing the MSPN substrate encoder in our ESPS framework with PTM-Mamba while keeping all other ESPS components identical. We include a detailed discussion of PTM-Mamba and its relationship to COMPASS-PTM in Supplementary Information S.3.4. Under the standard warm-start setting (random split), ESPS achieves high performance, consistently outperforming the leading specialized predictor Phosformer-ST, with an AUC of 86.33% and an AUPRC of 84.36% (Fig. 3a). To rigorously evaluate the model’s generalization capacity, we tested it under two cold-start scenarios, which were defined by partitioning substrates and enzymes using MMseqs2. In the substrate cold-start setting, ESPS again outperformed all baseline models across all key metrics (Fig. 3b), achieving an ACC of 0.769 and an AUC of 0.849. This strong performance underscores the model’s ability to capture intrinsic sequence features that determine substrate suitability, a capability we attribute to the discriminative substrate representations learned through the first stage’s crosstalk-aware training on multi-label PTM prediction. Under the more challenging enzyme cold-start scenario, ESPS remained highly competitive, performing on par with or better than most baselines and only slightly behind Phosformer-ST^29^ (Fig. 3c), indicating its robustness in generalizing to previously unseen regulatory contexts.

To further validate these findings, we assessed ESPS on the independent SAGEPhos benchmark under identical experimental conditions (Fig. 3d). ESPS consistently surpassed the SAGEPhos^20^ model on its own benchmark across all metrics, including accuracy (80.99%), AUC-ROC (88.57%), and AUC-PRC (86.88%). Notably, it reduced the False Positive Rate to 19.58% (compared to 21.7% by SAGEPhos), a critical improvement for ensuring that experimental validation efforts are directed toward high-confidence candidates.

It is critical to contextualize this comparison within the broader prediction workflow. While specialized tools achieve high performance on the discrete task of enzyme assignment, they address only the “fine” inference step, requiring a known site as input. COMPASS-PTM’s defining advantage allows it to operate from a full-length protein sequence alone, making it uniquely suited for scenarios where PTM sites are not known.

Building on our enzyme cold-start experiments that confirmed the model’s generalization to unseen enzymes, we further assessed our ESPS’s performance on the rigorous DARKIN benchmark^24^. This benchmark is designed to identify “dark kinases” for a given phosphosite, enforcing strictly disjoint kinase sets between the training and testing splits to ensure a true zero-shot evaluation. Model performance is quantified by the mean Average Precision (mAP), which assesses the ability to correctly rank true kinases for each substrate. For a detailed description of the DARKIN benchmark and the mAP evaluation protocol, see the Supplementary Information S.3.7. Following the benchmark’s protocol, our model achieves an mAP of 0.2946. This result substantially outperforms the highest reported score from the original study (0.1911) by 54%. Notably, this score was obtained using an ESM2-150M backbone, which has fewer parameters than several models evaluated in the original DARKIN study. This result suggests that ESPS can help prioritize candidate kinase-substrate associations for previously unseen kinases under the DARKIN zero-shot evaluation protocol.

### Learned representations and motifs reveal biochemical organization

To validate that COMPASS-PTM operates not as a “black box” but as a learner of genuine biochemical principles, we conducted a multi-faceted interpretability analysis.

**Fig. 4:**
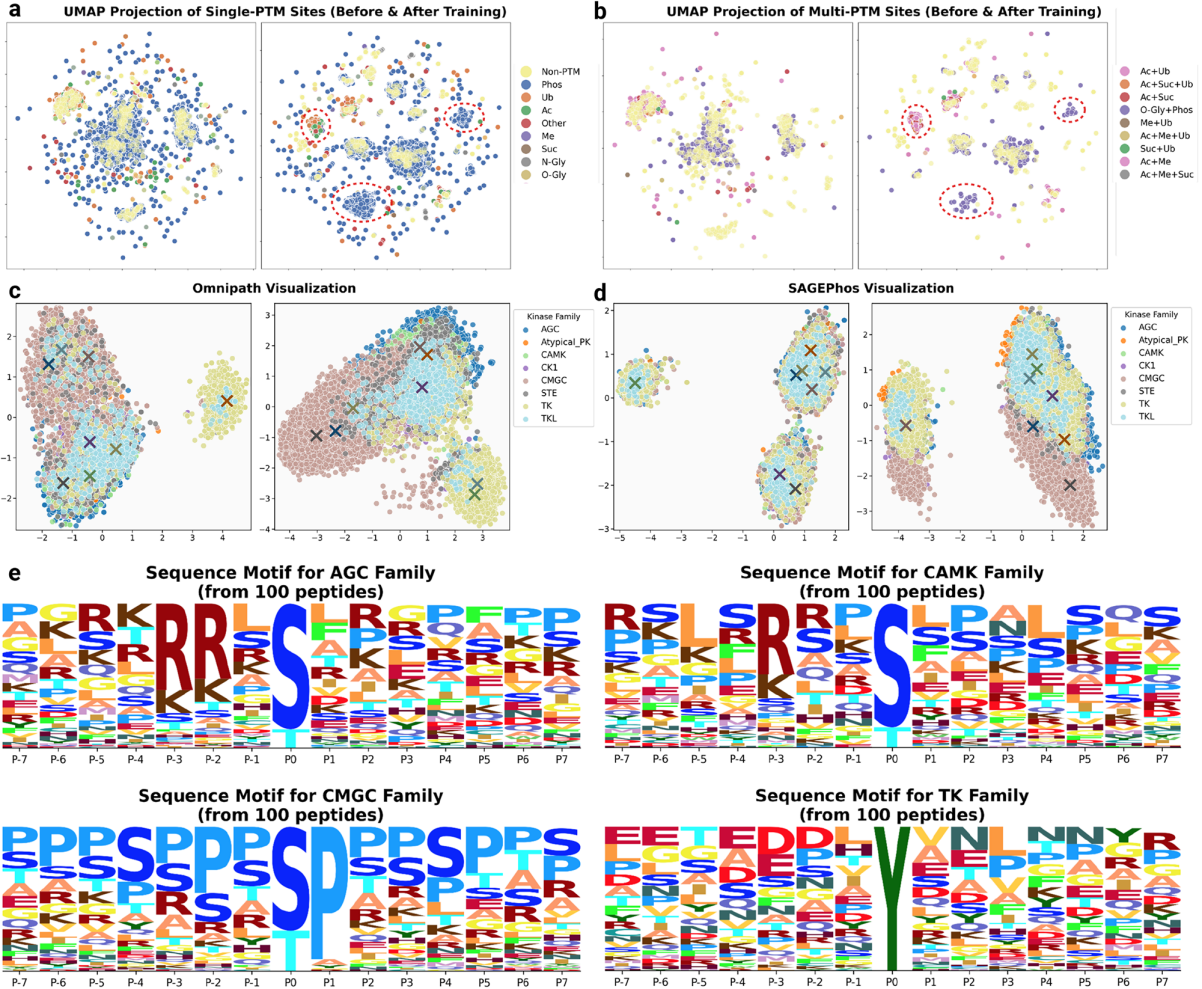
Visualizing the learned representations of the COMPASS-PTM framework. **a**,**b** UMAP projection^30^ of the Stage 1 embedding space for single-PTM (**a**) and multi-PTM (**b**) sites, shown before (left) and after (right) training. Red dashed outlines highlight representative clusters that become more resolved after training. **c**,**d** Principal Component Analysis (PCA)^31^ of Stage 2 embeddings, organized by kinase family, for the OmniPath (**c**) and SAGEPhos (**d**) datasets. For each dataset, clustering based on substrate-only features (left) is coarse, but becomes highly resolved after fusion with enzyme-specific information (right), demonstrating a coarse-to-fine learning dynamic. **e** Canonical kinase recognition motifs recovered from the model’s top-100 high-confidence predictions for four major kinase families. The sequence logos recover known kinase-family-associated motifs, including the basophilic motifs of AGC and CAMK, the proline-directed motif of CMGC, and the tyrosine-specific motif of TK, supporting the biological interpretability of the predictions. In the sequence logos, P0 denotes the central modified residue position.

We first visualized the learned representations of PTM sites to examine how training reshapes the stage 1 embedding space. UMAP (Uniform Manifold Approximation and Projection^30^) visualization reveals the progressive, biochemically-aware organization of PTM site representations learned during training. To elucidate this process, we visualized single- and multi-PTM sites separately, holding the projection of non-modified sites constant as a reference frame (Fig. 4a, b). For single-PTM sites (Fig. 4a), the initial pre-trained embeddings exist in an undifferentiated state, with PTM and Non-PTM sites heavily intermingled (left panel). Upon training, this space self-organizes into a well-defined and logical structure (right panel). The most fundamental transformation is the segregation of bona fide PTM sites from the Non-PTM background, demonstrating that the model learns to effectively separate biologically meaningful signals from noise. Deeper analysis reveals a sophisticated capacity to resolve fine-grained distinctions that mirror biochemical principles. For instance, the numerous phosphorylation sites resolve into several discrete sub-clusters, strongly suggesting the model has learned to distinguish different phosphorylation contexts, such as motifs recognized by distinct kinase families. The model also deciphers the complex “lysine code”: a subset of methylation forms an exclusive cluster, while acetylation and ubiquitination also form their own distinct groups. Although their clusters are in close proximity, they exhibit high internal cohesion, demonstrating that the model learns their unique signatures despite a shared substrate. Furthermore, structurally bulky modifications like N-linked glycosylation converge into a distinct cluster, highlighting the model’s ability to learn unique sequence grammars. A similar organizing principle is evident for multi-PTM sites (Fig. 4b). Initially randomly distributed, these sites converge into discrete clusters defined by specific PTM combinations after training. This demonstrates that COMPASS-PTM successfully learns crosstalk relationships, grouping sites that share common combinatorial modification patterns. In summary, this visualization provides supporting evidence that our model is not a “black box." Instead, it learns a biochemically coherent map where proximity in the embedding space reflects genuine functional relationships, enabling robust and interpretable predictions.

Second, to interrogate the hierarchical representations learned by our two-stage model, we visualized the substrate embeddings from the OmniPath and SAGEPhos datasets using Principal Component Analysis (PCA)^31^, both before and after fusion with enzyme information (Fig. 4c, d). First, we examined the substrate-only embeddings generated by Stage 1 to validate its ability to learn biologically relevant sequence patterns (left panel). The analysis reveals that the embeddings spontaneously organize according to the cognate kinase family that targets them. This emergent organization, a stark contrast to their initial random state (Supplementary Fig. 1j, k), provides supporting evidence that our model successfully extracts the conserved, and often subtle, substrate motifs characteristic of different kinase families. The partial overlap among families like AGC and STE accurately reflects known biological promiscuity in motif recognition. Next, we visualized the final, fused embeddings from Stage 2, which incorporate information from both the substrate and the specific kinase (right panel). The result is a striking reorganization into discrete, compact, and well-separated clusters, each corresponding to a specific kinase family. This separation is consistent with the model learning features relevant to kinase-family specificity and enzyme-substrate assignment. This progression from general motif learning to specific, accurate interaction prediction was consistently observed across datasets.

To further validate that the predictive accuracy of our Stage 2 model is rooted in a genuine understanding of molecular recognition, we designed an experiment to interrogate its learned representation of enzyme specificity. For each of the eight major kinase families, we selected up to 100 substrate peptides from the test set that COMPASS-PTM predicted with the highest confidence. By generating sequence logos from these high-confidence predictions, we could visualize the sequence features the model deemed most important for each family. The resulting sequence logos confirmed that COMPASS-PTM robustly recovered the canonical substrate motifs that serve as the biochemical signatures for these kinase families (Fig. 4e and Supplementary Fig. 4a–d). For the AGC family, which includes central regulators of cell growth and metabolism, such as PKA and Akt, the model correctly identified the classic basophilic motif. This was characterized by a strong preference for basic residues (Arginine/R, Lysine/K) at the P-3 and P-2 positions (where P0 denotes the modified residue and P-n/P+n denote upstream/downstream positions, respectively), conforming to the R-R-X-S/T consensus^32^. The model also captured the more complex, hierarchical requirements of the CAMK family, a group responsive to calcium signaling. It identified a stringent preference for Arginine/R at the P-3 position and, critically, a distinct requirement for a large hydrophobic residue (Leucine/L) at the distal P-5 position, consistent with the L-X-R-X-X-S/T motif^33^. For the CMGC family, comprising essential cell cycle regulators (e.g., CDKs) and signaling kinases (e.g., MAPKs), the model identified the unmistakable proline-directed motif, defined by a near-absolute requirement for Proline/P at both the P-2 and P+1 positions, recapitulating the P-X-S/T-P consensus^34,35^. Finally, for Tyrosine Kinases (TK), a family fundamental to growth factor signaling, the model’s learned representation extended far beyond the absolute specificity for Tyrosine (Y) at the P-0 site. It correctly captured the strong preference for upstream acidic residues that defines the acidophilic tyrosine motif^36^ and, furthermore, identified a subtle preference for Proline/P at the P+3 position, a feature critical for substrate binding and the assembly of downstream signaling complexes. The recovery of these motifs supports the interpretation that COMPASS-PTM captures sequence features associated with enzyme-substrate specificity.

### Mapping pathogenic mutations to PTM and enzyme rewiring

To assess whether COMPASS-PTM can link sequence variation to regulatory consequences, we applied it in two complementary settings. We first carried out a systematic screen for variants predicted to disrupt PTM events in the human sperm proteome. We next examined several disease-associated representative examples discussed in DeepMVP^16^, to evaluate whether COMPASS-PTM can recapitulate known PTM-related mechanisms and further resolve their associated enzyme-level changes.

For the systematic screen, we cross-referenced pathogenic variants from the PhosphoSitePlus PTMVar database^37^ against a comprehensive catalog of the human sperm proteome^38^. Using a stringent filtering criterion, defined as a wild-type prediction score greater than 0.8 and a mutant prediction score less than 0.2, we identified 62 high-confidence variants predicted to abolish PTM events (Supplementary Table 3). Among these hits, our analysis highlighted several compelling cases directly linked to reproductive pathologies. A notable example is the S2621C variant in the Adenomatous Polyposis Coli (APC) protein, a mutation associated with Familial Adenomatous Polyposis 1 (FAP1). While primarily a cancer syndrome, APC is also vital for normal spermatogenesis^39^. COMPASS-PTM predicted that this mutation causes a marked decrease of a key phosphorylation site, with the prediction score decreased from 0.944 in the wild-type to a negligible 0.0002 in the mutant. Another significant finding involved HUWE1, a HECT-domain E3 ubiquitin ligase associated with Turner-type X-linked syndromic intellectual developmental disorder (MRXST). The K4295N variant lies within the C-terminal HECT catalytic domain and has been linked to MRXST, a neurodevelopmental disorder that can include male reproductive abnormalities^40^. COMPASS-PTM predicted that K4295N abolishes a putative ubiquitination site on HUWE1, with the score decreasing from 0.869 in the wild type to 0.0003 in the mutant. These examples highlight the ability of COMPASS-PTM to generate testable hypotheses linking variants to disease phenotypes through disrupted PTM-mediated regulation. We next ask whether the same framework can be used to trace how individual pathogenic variants rewire not only PTM sites but also their cognate enzyme-substrate relationships.

Beyond large-scale screening, we sought to rigorously assess, at single-variant resolution, how COMPASS-PTM links mutations to PTM programs and upstream regulatory enzymes. Importantly, by combining site-level outputs from MSPN with enzyme-substrate scores from ESPS, the framework can quantify how pathogenic variants simultaneously rewire PTM probabilities and the recognition strength of specific regulatory enzymes. In the following case studies, we first validate that COMPASS-PTM can computationally recapitulate established pathogenic pathways and then exploit this joint representation to generate mechanistic hypotheses for less well-characterized variants, closing the loop from a single missense mutation to the loss or gain of PTM events and the corresponding kinases or ligases.

**Fig. 5:**
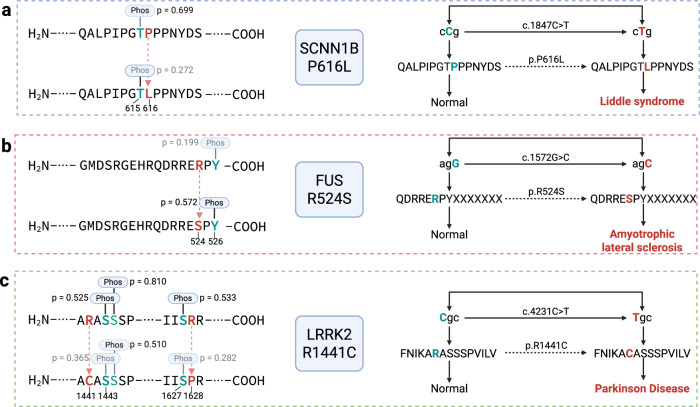
COMPASS-PTM generates mechanistic hypotheses by predicting the PTM consequences of pathogenic variants. **a** The Liddle syndrome-associated p.P616L substitution in SCNN1B is predicted to cause a loss of phosphorylation at the adjacent T615 site (predicted probability drops from 0.70 to 0.27)^42^. **b** The amyotrophic lateral sclerosis (ALS)-associated p.R524S variant in FUS is predicted to induce a gain of phosphorylation at the nearby Y526 site (probability increases from 0.20 to 0.57)^43,44^. **c** Two Parkinson’s disease-associated variants in LRRK2, p.R1441C and p.R1628P, are both predicted to result in a loss of phosphorylation at their respective proximal sites^41^. For each case, the diagrams on the left illustrate the local sequence and predicted PTM probabilities, where the pathogenic substitution is highlighted in red and the affected PTM site is in green. The diagrams on the right summarize the underlying genetic alteration and resulting pathology.

We began by examining two well-studied mutations in the LRRK2 gene, a key locus in Parkinson’s disease. Confirming its accuracy, our stage1 MSPN model predicted that the R1441C variant leads to a loss of phosphorylation at S1443 (Fig. 5c). In parallel, stage2 ESPS model assigned PKA as the cognate kinase for this site and reported a concordant decrease in the enzyme-substrate interaction score from 0.58 in the wild type to 0.46 in the R1441C mutant, indicating weakened PKA recognition of the mutated motif. This dual-level prediction is fully consistent with extensive experimental evidence that this mutation impairs PKA-mediated phosphorylation, thereby disrupting LRRK2’s interaction with 14-3-3 regulatory proteins—a cornerstone of its pathogenic mechanism^41^. The model likewise correctly identified that the R1628P variant causes a loss of phosphorylation at S1627, further validating its ability to pinpoint PTM-altering events in a complex disease protein.

Having established the model’s accuracy, we then deployed it as a hypothesis-generation engine. For the P616L variant in SCNN1B, which causes Liddle syndrome, the model proposes a precise molecular basis for the known overactivation of the ENaC channel. MSPN predicts a substantial decrease of phosphorylation at T615 (Fig. 5a), a site whose modification is the known signal for the channel’s removal from the cell surface^42^. Consistently, ESPS suggests that this site is targeted by ERK1/2 (represented by MAPK3) and that P616L weakens this regulatory link, with the corresponding kinase-substrate score decreasing from 0.55 in the wild type to 0.47 in the mutant. Together, these predictions suggest that P616L may weaken ERK1/2-associated regulation of ENaC internalization, providing a testable hypothesis for altered channel stability. In another case, the model generated a hypothesis for the R524S variant in the FUS protein, which is associated with ALS^43^. MSPN predicts a gain of phosphorylation at Y526 (Fig. 5b), and ESPS assigns this site to Src-family tyrosine kinases. Notably, the predicted Src-substrate interaction score is slightly increased in the mutant compared with the wild type, suggesting that R524S enhances Src-family recognition and thereby amplifies a PTM event that is already known to impair FUS nuclear import, exacerbating its toxic cytoplasmic aggregation^43,44^. See the Supplementary Information S.3.9 for more case studies.

Collectively, these applications demonstrate COMPASS-PTM’s versatility as an engine for translational research. It can efficiently prioritize candidates from large-scale genetic data while also providing the granular, mechanistic insights needed to design focused experiments, bridging the gap between genomic variation and disease pathology.

### Clinically relevant case studies highlight biologically coherent PTM predictions

**Fig. 6:**
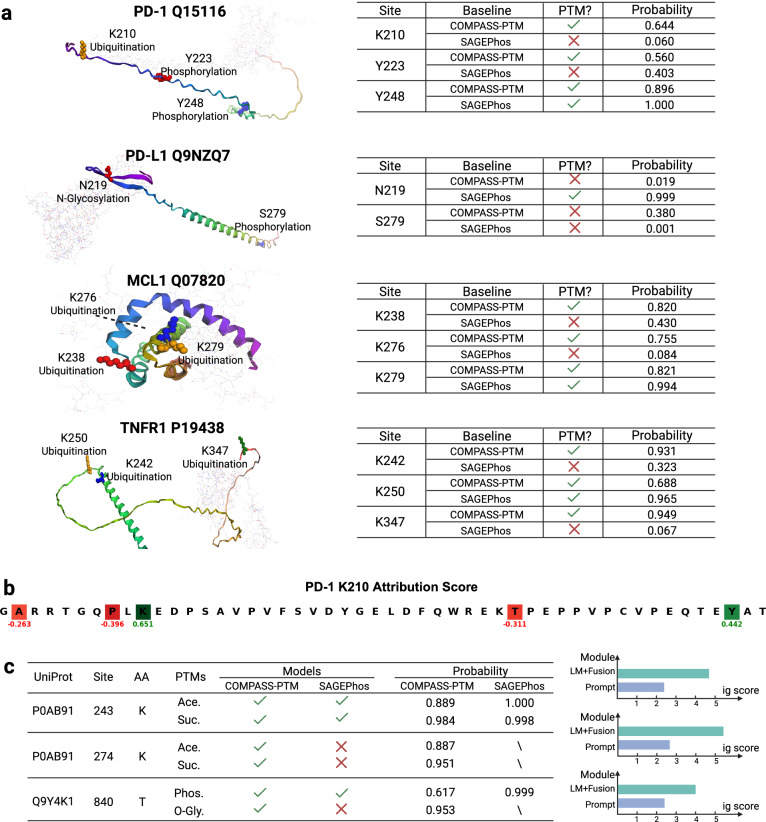
Case studies demonstrating the accuracy and mechanistic interpretability of COMPASS-PTM on clinically relevant targets. **a** Zero-shot prediction of functionally critical PTMs on four disease-associated proteins. The tables compare the predictions of COMPASS-PTM against the baseline (SAGEPhos^20^) for sites that were withheld from the training set. Correct predictions are marked with a green check and incorrect predictions with a red cross. **b** Gradient-based attribution analysis for the prediction of ubiquitination at K210 on PD-1. The height and color of each residue indicate its contribution to the final prediction score (green for positive, red for negative), revealing the sequence determinants the model learned. **c** Superior performance in complex multi-label prediction on sites with co-occurring PTMs. The bar plots on the right show attribution scores for the model’s core components, quantifying the significant contribution of the crosstalk-aware Prompt module to the correct classification of these sites. Ace., acetylation; Suc., succinylation; Phos., Phosphorylation; O-Gly., O-linked glycosylation; ig, integrated gradients.

To assess COMPASS-PTM’s capacity for testable discovery on clinically relevant targets, we performed zero-shot predictions on four proteins central to immune regulation and cell survival: PD-1, PD-L1, MCL1, and TNFR1 (Fig. 6a). By benchmarking against the top baseline, SAGEPhos^20^, on PTM sites not present in the training set, we evaluated the model’s ability to identify functionally critical regulatory sites.

PD-1 reveals superior detection of immune checkpoint regulatory modifications. On PD-1, a key immune checkpoint receptor, COMPASS-PTM successfully identified pivotal regulatory modifications missed by the baseline^45^. It correctly predicted ubiquitination at K210 (score: 0.644 vs. 0.060), a PTM that terminates the inhibitory signal by triggering receptor degradation^46^. Furthermore, it accurately identified phosphorylation at Y248 (score: 0.896), the crucial event for recruiting SHP-2 phosphatases to execute T-cell inhibition^47^. This ability to pinpoint distinct molecular switches underscores the model’s precision in deciphering immune signaling.

PD-L1 modifications reveal complementary predictive capabilities across PTM types. The analysis of PD-L1 revealed complementary strengths between the models, highlighting the complexity of the PTM code^48^. While SAGEPhos showed high-confidence prediction of N-glycosylation at N219 (0.999 vs. 0.019), a PTM that stabilizes the protein, COMPASS-PTM uniquely identified phosphorylation at S279 (0.380 vs. 0.001), a site completely missed by the baseline. This demonstrates how different architectures may capture distinct biochemical principles.

MCL1 ubiquitination patterns demonstrate superior detection of apoptosis regulatory sites. For the anti-apoptotic protein MCL1, whose stability is governed by ubiquitination-mediated degradation, COMPASS-PTM showed superior detection of key “degron" sites^49^. It confidently identified critical turnover sites at K238 (0.820 vs. 0.430) and K276 (0.755 vs. 0.084), sites where the baseline was either less confident or failed to make a positive prediction. This robust performance on functionally validated degrons highlights its potential to identify key regulatory switches in cell survival pathways.

TNFR1 ubiquitination demonstrates comprehensive regulatory site detection. On TNFR1, a receptor that governs inflammatory and cell death pathways, COMPASS-PTM excelled at identifying ubiquitination sites critical for signaling complex assembly^50^. It demonstrated markedly superior performance in detecting ubiquitination at K242 (0.931 vs. 0.323) and K347 (0.949 vs. 0.067), residues essential for recruiting adapters to the pro-survival “Complex I,” showcasing its ability to predict the assembly points for critical cell fate decisions.

Interpretability analysis reveals mechanistic insights into PTM Code. To dissect the sequence determinants underpinning its predictive engine, we employed a robust gradient-based attribution analysis focused on the ubiquitination of lysine 210 (K210) in PD-1 (Fig. 6b). The analysis revealed that: the phosphorylation site at tyrosine 248 (Y248)—the principal switch for PD-1’s inhibitory function—emerged as a dominant predictive feature for ubiquitination at the distal K210 site. This attribution pattern is consistent with the model using information from a functionally relevant phosphorylation site when predicting distal ubiquitination, a relationship that may occur through phospho-degron-associated mechanisms^51^. This ability to integrate signals from a distributed network of local, distal, and key functional residues confirms that COMPASS-PTM transcends simple motif-matching, leveraging its dual-modal architecture to achieve its high accuracy.

COMPASS-PTM Excels in Multi-Label Prediction by Learning Biochemical Constraints. A critical test for any PTM predictor is its ability to accurately profile sites harboring multiple, concurrent modifications. We benchmarked COMPASS-PTM against the top-performing baseline, SAGEPhos, and found that our model not only achieves higher accuracy but, critically, generates more biochemically coherent predictions (Fig. 6c). For instance, on protein P0AB91, COMPASS-PTM correctly identifies the co-occurrence of acetylation and succinylation on two distinct lysine residues (K243 and K274), whereas SAGEPhos fails at the latter site, indicating a sensitivity gap. More strikingly, on a threonine residue (T840) of protein Q9Y4K1, COMPASS-PTM successfully resolves the complex crosstalk between phosphorylation and O-linked Glycosylation. SAGEPhos, while correctly identifying the phosphorylation event with high confidence, completely fails to detect the concurrent O-linked Glycosylation. A deeper analysis of the SAGEPhos predictions for T840 rsuggests a limitation in its learning paradigm. While the baseline model can assign high probabilities to some correct predictions, this appears to be a symptom of a broader tendency to generate spurious, high-probability outputs. On the threonine T840 site, SAGEPhos not only correctly identifies phosphorylation but also erroneously predicts two lysine-exclusive modifications—acetylation and ubiquitination—with biochemically impossible and misleadingly high probabilities. In contrast, COMPASS-PTM’s ability to consistently generate plausible predictions suggests it has learned these foundational rules. The source of this superior biochemical reasoning is quantifiable: a gradient-based attribution analysis shows our crosstalk-aware prompting mechanism—the module explicitly modeling PTM dependencies—consistently accounts for approximately one-third of the predictive power in these complex cases (Fig. 6c, right panels).

### Systematic ablation studies validate the COMPASS-PTM architecture

To systematically validate our design principles, we conducted a series of ablation experiments to interrogate the contribution of each component in the COMPASS-PTM framework. For the Stage 1 Multi-label Site Profiling Network (MSPN), we first confirmed our empirical choice of encoders—ESM2-150M for the protein branch and a SELFIES-based chemical language model for the chemical branch—as well as the superiority of our proposed dual-modal fusion architecture over simpler alternatives. More critically, these studies quantified the substantial impact of our key innovations. The crosstalk-aware prompting module proved essential for modeling PTM interdependencies, as its removal resulted in a 10.7% decrease in F1-score and a 9.8% decrease in MCC. Parameter-efficient fine-tuning with LoRA was also important, boosting overall performance by approximately 30% and precision by 44%, which underscores the necessity of adapting the large PLM to the specific task of PTM profiling. Our hybrid loss function was confirmed to be highly effective, successfully balancing the precision-recall trade-off to outperform standard loss functions in the context of the severe dual long-tail data distribution. Detailed descriptions and full results of these ablation experiments are provided in Supplementary Note S.3.11.1 and Supplementary Fig. 6a–f. Moreover, our model component ablations further confirm that the performance gains are not attributable to any single ingredient: the PLM-anchored dual-modal design yields substantial improvements over a PLM-only baseline, and removing the CLM branch from the full pipeline leads to a pronounced degradation, supporting genuine multimodal synergy. We further dissected the prompt matrix used by the crosstalk-aware prompting module by comparing the PTMCode2-initialized prior with a strict de-duplicated prior recomputed after removing proteins overlapping with validation/test splits. The strict prior remained nearly identical to the original prompt matrix and performed indistinguishably from the default COMPASS-PTM initialization, while both outperformed a learnable random initialization. These results indicate that the prompt acts as a soft, learnable inductive bias rather than a benchmark-specific lookup mechanism (see Supplementary Note S.3.11.1 and Supplementary Fig. 7 for full details).

The design of the Stage 2 Enzyme-Substrate Pairing System (ESPS) was similarly validated. Our proposed dual-gated residual fusion module demonstrated superior performance against standard fusion strategies, including concatenation and cross-attention, confirming its effectiveness in selectively integrating the most informative features from both the enzyme and substrate. Furthermore, we determined that a 15-amino-acid peptide length provided the optimal local context for the pairing task, yielding higher performance than both shorter and longer sequences. Together, these analyses provide a clear rationale for our methodological choices and quantify the synergistic contributions of each component to the framework’s overall success (see Supplementary Note S.3.11.2 and Supplementary Fig. 6g, h for full details).

## Discussion

In this work we recast PTM prediction as a mechanism-aware learning problem and show that a single representation can support both site-level and enzyme-level inference from sequence alone. COMPASS-PTM operationalizes this view with a two-stage, coarse-to-fine architecture in which a Multi-label Site Profiling Network (MSPN) first learns PTM programs on substrates, and an Enzyme-Substrate Pairing System (ESPS) then refines these programs into specific enzyme-substrate relationships. Together, these components bridge the long-standing gap between predicting where modifications occur and who writes or erases them, yielding outputs that are directly interpretable in terms of regulatory enzymes.

Beyond the benchmark results, our work shows that structured biological priors can improve interpretability in a deep learning model for PTM prediction. Two key design choices are critical. First, the crosstalk-aware prompting module leverages PTM co-occurrence data as an inductive bias, improving performance on rare and co-modified sites. Second, and more powerfully, the unified representation linking sites and enzymes allows the model to connect genetic variation to functional consequences. We support this interpretation by showing that COMPASS-PTM associates missense variants with predicted changes in local PTM probabilities and enzyme-substrate scores, including examples consistent with reported LRRK2-related mechanisms in Parkinson’s disease and testable hypotheses for less characterized variants. The model’s interpretability is further underscored by its unsupervised recovery of canonical kinase motifs and its ability to organize enzymes and substrates into biochemically coherent families.

At the same time, several limitations and caveats deserve emphasis. First, COMPASS-PTM is ultimately constrained by the coverage, quality and biases of current PTM resources. The training datasets exhibit strong dual long-tail behavior, an over-representation of well-studied PTM types and enzymes, and under-sampling of context-specific or transient modifications, which may lead to overly confident predictions in poorly characterized regimes. Second, while our crosstalk-aware prompting is initialized from external PTM co-occurrence statistics and curated interaction resources, in this work the prior is used strictly as a global, type-level statistic and does not include protein-, site-, or sequence-resolved benchmark annotations. As a result, it does not constitute direct, instance-level test-set label leakage in the present evaluation. Nevertheless, as COMPASS-PTM is extended to incorporate richer priors—such as protein-/site-resolved interaction graphs or context-specific regulatory networks—careful de-duplication and transparent reporting of data provenance will remain essential to avoid circularity. Third, the current model primarily encodes linear sequence information. It does not explicitly account for higher-order structural features, cellular context, or temporal dynamics of signaling, all of which are known to modulate PTM accessibility and enzyme specificity. In addition, ESPS represents substrates using a site-centered 15-mer, which is well-suited for capturing proximal recognition motifs but cannot explicitly model distal docking interactions or long-range domain/scaffold effects.

These limitations suggest several concrete directions. Integrating structure-aware representations—for example, from experimentally determined or AlphaFold-like models—could help disentangle sequence and structural determinants of PTM regulation. Coupling COMPASS-PTM with context-specific information, such as expression, subcellular localization, or phosphoproteomic time courses may enable modeling of condition-dependent PTM programs rather than context-agnostic propensities. From a modeling perspective, extending the coarse-to-fine paradigm to jointly learn generative models of PTM programs and enzyme-substrate networks could support design tasks, such as predicting the impact of engineered mutations or kinase inhibitors on signaling pathways. Finally, as we demonstrate in variant-centric case studies, the outputs of COMPASS-PTM should be interpreted as hypotheses rather than definitive mechanistic claims, and will be most powerful when used to prioritize focused experimental tests of PTM and enzyme rewiring in specific disease settings.

More broadly, our findings illustrate that coupling statistical learning with explicit mechanistic priors is a promising strategy for decoding biochemical “codes”. While COMPASS-PTM is instantiated for PTMs and their enzymes, the same design principles—multi-label program inference, shared coarse-to-fine representations, and biologically motivated prompting—should be transferable to other regulatory layers, including enzymatic cascades, proteolytic processing, and protein-protein interaction networks. We anticipate that such mechanism-aware frameworks will help move machine learning for molecular biology from predicting isolated events toward building testable, system-level models of cellular regulation.

## Methods

### Problem formulation

We address two coupled prediction tasks concerning PTMs.

Task 1: Multi-label PTM Site Profiling. The primary task is formalized as a residue-wise multi-label classification problem. Given a substrate protein sequence $S={\{{s}_{i}\}}_{i=1}^{L}$, the objective is to predict a *C*-dimensional binary label vector **y**_*i*_ ∈ {0, 1}^*C*^ for each residue *s*_*i*_, where *C* is the number of PTM types considered for a given task. Each dimension in this vector corresponds to a specific modification class (e.g. phosphorylation, acetylation) known to target distinct amino acid residues. The prediction function *f*_*θ*_ thus maps a sequence and a position to a probabilistic output for each modification type *c*:

1 $${y}_{i}^{(c)}={f}_{\theta }(S,i,c),\,\forall c\in \{1,\ldots,C\}$$

where ${\mathbb{P}}({y}_{i}^{(c)}=1| S)\in [0,1]$ is the predicted probability for the residue-type pair (*s*_*i*_, *c*).

Task 2: Enzyme-Substrate Pairing. The second, more fine-grained task is to identify the specific enzyme responsible for a modification. Given a substrate sequence *S* and an enzyme sequence $E={\{{e}_{j}\}}_{j=1}^{M}$, the prediction function *g*_*ϕ*_ aims to identify the specific site of modification:

2 $${z}_{i}^{(c,E)}={g}_{\phi }(S,E,i,c),\,\forall c\in \{1,\ldots,C\},\forall E\in {{\mathcal{E}}}_{c}$$

where ${\mathbb{P}}({z}_{i}^{(c,E)}=1| S,E)\in [0,1]$ represents the probability that enzyme *E* from the set of valid catalysts ${{\mathcal{E}}}_{c}$ performs a type-*c* modification at residue *s*_*i*_.

We developed COMPASS-PTM, a unified coarse-to-fine two-stage computational framework, to address these tasks. The first stage, the Multi-label Site Profiling Network (MSPN), performs Task 1. The second stage, the Enzyme-Substrate Pairing System (ESPS), leverages the outputs of the first stage to perform Task 2.

### Stage 1: multi-label crosstalk-aware site profiling network

The first stage of our framework is designed to predict multi-label PTM sites while explicitly modeling the interdependencies between different modification types.

#### Adaptive segmentation

For Stage 1 training, we convert each full-length protein sequence $S={\{{s}_{i}\}}_{i=1}^{L}$ of length *L* with an annotated PTM-site set ${\mathcal{P}}={\{{p}_{k}\}}_{k=1}^{M}$ of size *M* into a set of substrate peptides ${\mathcal{W}}={\{({l}_{t},{r}_{t})\}}_{t=1}^{T}$ of size *T* via an adaptive greedy segmentation procedure. Each peptide is a contiguous subsequence *S*[*l*_*t*_: *r*_*t*_] subject to two constraints: (*i*) *r*_*t*_ − *l*_*t*_ + 1≤50, and (*ii*) it contains at least one annotated PTM site, that is, ${\mathcal{P}}\cap [{l}_{t},{r}_{t}]\ne {{\varnothing }}$.

Algorithmically, we process PTM sites in ascending order and iteratively form the next peptide to cover the leftmost uncovered site, choosing boundaries (*l*_*t*_, *r*_*t*_) to include as many subsequent nearby PTM sites as possible while respecting the 50-aa limit. This yields a compact set of informative peptides, avoids all-negative fragments, and reduces the redundant overlap induced by fixed-step sliding windows. Full details are provided in Supplementary Notes S.3.1.2.

#### Dual-modal feature representation

Our framework employs dual-branch encoding to derive a comprehensive representation for substrate peptides *S*_peptide_, integrating complementary large-scale biological context and fine-grained physicochemical properties.

Biological context modality. We leverage the evolutionary-scale knowledge within the protein language model ESM2-150M^52^ to extract context-aware features ${{\bf{H}}}_{s}^{{\rm{prot}}}\in {{\mathbb{R}}}^{{d}_{p}}$, where *d*_*p*_ = 640. To specialize the protein language model for PTM substrate recognition, we implement low-rank adaptation (LoRA)^53^, a parameter-efficient fine-tuning technique.

This adaptation enhances detection of evolutionarily conserved motifs and structural propensities pertinent to modification sites.

Chemical attribute encoding. To capture atomic-level properties, we generate a unified chemical representation ${{\bf{H}}}_{s}^{{\rm{chem}}}$ by concatenating 320-dimensional embeddings produced by a chemical language model (CLM) operating on SELFIES-based molecular representations with explicit per-residue physicochemical descriptors **P**_*s*_ (e.g. hydrophobicity and polarity).

#### Bio-coupled and augmented fusion

To effectively integrate the rich, evolutionarily informed features from protein language models, ${{\bf{H}}}_{s}^{{\rm{prot}}}$, with potentially noisy residue-based chemical attributes, ${{\bf{H}}}_{s}^{{\rm{chem}}}$, we developed a Bio-Coupled and Augmented Fusion module. This approach establishes a clear information hierarchy: the robust biological representation serves as the primary modality, which is then selectively refined and augmented by the auxiliary chemical information.

The fusion process is governed by a dynamic mechanism that additively combines a gated component, ***Φ***, and a residual component, ***Ψ***, controlled by learnable parameters *α* and *β*:

3 $${{\bf{H}}}_{s}^{{\rm{fused}}}=\alpha \,{\boldsymbol{\Phi }}\left({{\bf{H}}}_{s}^{{\rm{prot}}},{{\bf{H}}}_{s}^{{\rm{chem}}}\right)+\beta \,{\boldsymbol{\Psi }}\left({{\bf{H}}}_{s}^{{\rm{prot}}},{{\bf{H}}}_{s}^{{\rm{chem}}}\right)$$

Here, the gating mechanism ***Φ*** learns to filter the chemical features, preserving the primary protein signal while allowing only the most task-relevant chemical information to modulate it. Concurrently, the residual mechanism ***Ψ*** is designed to capture valuable cross-modal interactions, enriching the primary feature space with complementary chemical context. The details of both the gating and residual mechanisms are provided in Supplementary Methods S.4.1.1.

#### Crosstalk-aware prompting PTM attention

A key innovation of our model lies in conceptualizing PTM interdependence as a crosstalk-aware prompting mechanism. To model the biological principle that PTMs do not occur in isolation, we designed a custom attention mechanism that incorporates PTM interdependencies as an inductive bias. This is achieved by injecting a dynamic prompt into the self-attention calculation.

The foundation of this prompt is a learnable *C* × *C* matrix **R**, which is initialized with a prior based on known PTM co-occurrence statistics, **R**_prior_, and fine-tuned during training to capture complex, non-linear dependencies. The details of **R** and **R**_prior_ are in Supplementary Section S.4.1.2.

Crucially, this learned matrix is used to generate a dynamic prompt tailored to the specific context of each residue. For each position *i* in a sequence, the model first generates a preliminary PTM probability distribution

4 $${\bf{p}}_{i}={\mathrm{softmax}}\left({\mathrm{PTM}}-{\mathrm{Predictor}}({\bf{H}}_{i})\right)$$

from its final hidden-state embedding **H**_*i*_.

Using these probabilities, we dynamically compute a site-pair-specific attention prompt **B**. The prompt value between any two positions *i* and *j* quantifies the biochemical plausibility of their co-modification, mediated by the learned relationship matrix **R**. This interaction term is passed through a $\tanh$ activation, regularized with dropout, and scaled by a learnable parameter *γ*:

5 $${B}_{ij}={\rm{Dropout}}\left(\tanh \left({{\bf{p}}}_{i}^{\top }{\bf{R}}{{\bf{p}}}_{j}\right)\right)\gamma$$

The resulting bias matrix **B** is added directly to the attention logits, thereby guiding the model to focus on residue pairs with high predicted biochemical synergy:

6 $${\rm{Attention}}({\bf{Q}},{\bf{K}},{\bf{V}})={\rm{softmax}}\left(\frac{{\bf{Q}}{{\bf{K}}}^{\top }}{\sqrt{{d}_{k}}}+{\bf{B}}\right){\bf{V}}$$

By compelling the model to reason about PTMs as an interconnected system, this crosstalk-aware prompting mechanism ensures that the output is not merely a set of independent site predictions, but a single, biologically coherent combinatorial profile.

#### Balanced training objective

To address the severe data imbalance inherent in PTM prediction, characterized by both a long-tail distribution across PTM types (inter-class imbalance) and a vast excess of unmodified sites (intra-class imbalance), we designed a hybrid loss function. This objective combines a macro-level regularized Dice loss ${{\mathcal{L}}}_{{\rm{macro}}}$ with a micro-level focal loss ${{\mathcal{L}}}_{{\rm{micro}}}$, with their contributions balanced by a learnable parameter *η*:

7 $${{\mathcal{L}}}_{{\rm{stage1}}}=\eta {{\mathcal{L}}}_{{\rm{macro}}}+(1-\eta ){{\mathcal{L}}}_{{\rm{micro}}}$$

The macro-level component ${{\mathcal{L}}}_{{\rm{macro}}}$ is designed to tackle inter-class imbalance. It is composed of a Dice loss ${{\mathcal{L}}}_{{\rm{Dice}}}$, which ensures that rare PTM types contribute equally to the training objective, and a custom Magnification loss ${{\mathcal{L}}}_{{\rm{Mag}}}$, weighted by a learnable parameter *ω*:

8 $${{\mathcal{L}}}_{{\rm{macro}}}={{\mathcal{L}}}_{{\rm{Dice}}}+\omega {{\mathcal{L}}}_{{\rm{Mag}}}$$

The Magnification loss acts as a direct regularizer on the output probabilities to mitigate over-confidence and improve model calibration, which is particularly crucial for sparse data:

9 $${{\mathcal{L}}}_{{\rm{Mag}}}={\mathbb{E}}\left[\sigma (\widehat{{\bf{y}}})\right]$$

where *σ* represents the sigmoid activation function, which transforms the model’s raw output logits $\widehat{{\bf{y}}}$ into class-wise probabilities.

The micro-level component ${{\mathcal{L}}}_{{\rm{micro}}}$ is the focal loss, which addresses the intra-class imbalance between the few modified sites and the vast number of unmodified ones. The detailed mathematical formulations for both ${{\mathcal{L}}}_{{\rm{Dice}}}$ and ${{\mathcal{L}}}_{{\rm{micro}}}$ are provided in Supplementary Methods S.4.1.3.

### Stage 2: enzyme-substrate pairing system

The second stage predicts enzyme-substrate specificity by integrating features from both the enzyme and the substrate. We freeze the weights of the Stage 1 network and use it to generate a local substrate representation **H**_substrate_ for a 15-mer peptide centered on the candidate site. The global enzyme representation **H**_enzyme_ is derived from its full-length sequence using ESM-2-150M^52^.

To model the binding interaction, we developed a Dual-Gated Residual Fusion module. This architecture concurrently preserves essential information from both inputs while capturing emergent interaction features. It employs two independent gating mechanisms, **g**_substrate_ and **g**_enzyme_, to selectively retain salient features from the original inputs, while a parallel residual network captures emergent binding characteristics, **H**_residual_. These three components are integrated via a learnable weighted sum:

10 $${{\bf{H}}}_{{\rm{fused}}}={\alpha }_{0}\left({{\bf{g}}}_{{\rm{substrate}}}\odot {{\bf{H}}}_{{\rm{substrate}}}\right)+{\alpha }_{1}\left({{\bf{g}}}_{{\rm{enzyme}}}\odot {{\bf{H}}}_{{\rm{enzyme}}}\right)+{\alpha }_{2}{{\bf{H}}}_{{\rm{residual}}}$$

The learnable scalars *α*_*i*_ enable the model to dynamically balance the contributions of substrate motifs and global enzyme features, enhancing prediction robustness. Further implementation details are provided in Supplementary Methods S.4.2.1.

### Software and computational environment

All experiments were conducted in a Python 3.12 environment. The main software dependencies included PyTorch 2.5.1 with CUDA 12.1, fair-esm 2.0.0, transformers 4.48.1, peft 0.17.1, scikit-learn 1.7.2, pandas 2.3.3, scipy 1.16.2, and numpy 2.3.3. Sequence clustering and split construction were performed using MMseqs2 v16.747c6. The full software environment is provided in the released code repository.

### Reporting summary

Further information on research design is available in the Nature Portfolio Reporting Summary linked to this article.

## Supplementary information

Supplementary Information

Reporting Summary

Transparent Peer Review file

## Source data

Source Data

## Data Availability

The COMPASS-PTM datasets generated and curated in this study have been deposited in Zenodo and are publicly available at 10.5281/zenodo.18389225^54^. These files include the curated benchmark datasets, processed annotations, and train/validation/test splits used in this study. Source data are provided with this paper. The raw data used in this study are available from public databases. For the first stage (Multi-label Site Profiling), the raw PTM data used in this study are available in the dbPTM database (https://biomics.lab.nycu.edu.tw/dbPTM/download.php), qPTM database (https://qptm.omicsbio.info/download.php), and PTMint database (https://ptmint.sjtu.edu.cn/Download). For the second stage (Enzyme–Substrate Pairing), the raw enzyme–substrate relationship data used in this study are available in the OmniPath database (https://omnipathdb.org/) and the SAGEPhos dataset repository (https://github.com/ZhangJJ26/SAGEPhos/releases). Full-length protein sequences used in this study are available in UniProt (https://www.uniprot.org/). Source data are provided with this paper.

## Code availability

The custom code used for data processing, model training, and evaluation in this study is publicly available at GitHub (https://github.com/ZhangJJ26/COMPASS-PTM). An archived version corresponding to the exact release used in this study is available at Zenodo (10.5281/zenodo.19625754^55^).
