# Supplementary material for: Learning the PTM code through a coarse-to-fine mechanism-aware framework
Source: Nat Commun. 2026 May 15;17:6450. doi: 10.1038/s41467-026-73148-3 (PMC13377107; doi:10.1038/s41467-026-73148-3)
Supplement: Supplementary file 1 — Supplementary Information [file 41467_2026_73148_MOESM1_ESM.pdf]

S.1 Supplementary Figures

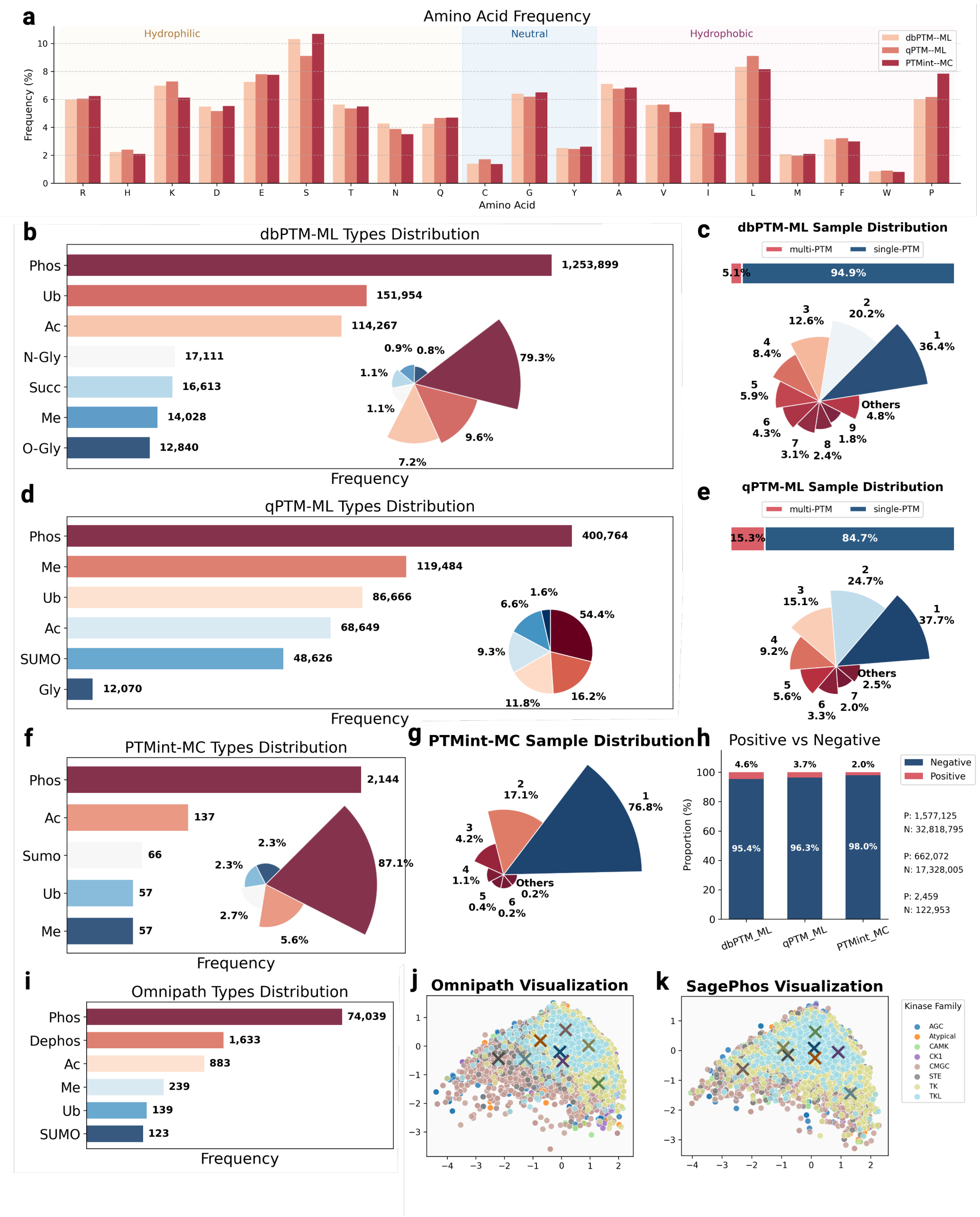

Supplementary Fig. 1: **Overview of the Benchmark Datasets. a**, The amino acid frequencies of the main datasets in Stage 1. **b-c**, Key statistics for the dbPTM-ML dataset, showing (**b**) the frequency of different PTM types, and (**c**) the ratio of single-PTM to multi-PTM sites with a further breakdown of how many sites have 2, 3, or more PTMs. **d-e**, The same statistical analysis performed on the qPTM-ML dataset. **f**, **g**, PTM type and sample distributions for the single-label PTMint-MC dataset. **h**, The ratio of modified (Positive) to unmodified (Negative) sites, highlighting the large class imbalance in the three datasets. **i-k**, A look at the datasets used for Stage 2 enzyme-substrate prediction, showing (**i**) top-6 PTM type frequencies in OmniPath, and (**j**, **k**) a PCA visualization of the initial substrate embeddings, colored by their corresponding kinase family, for the OmniPath (**j**) and SAGEPhos (**k**) datasets. Phos, phosphorylation; Ub, ubiquitylation; Ac, acetylation; Me, methylation; N-Gly, N-linked glycosylation; O-Gly, O-linked glycosylation; Succ, succinylation; SUMO, SUMOylation; Gly, glycosylation.

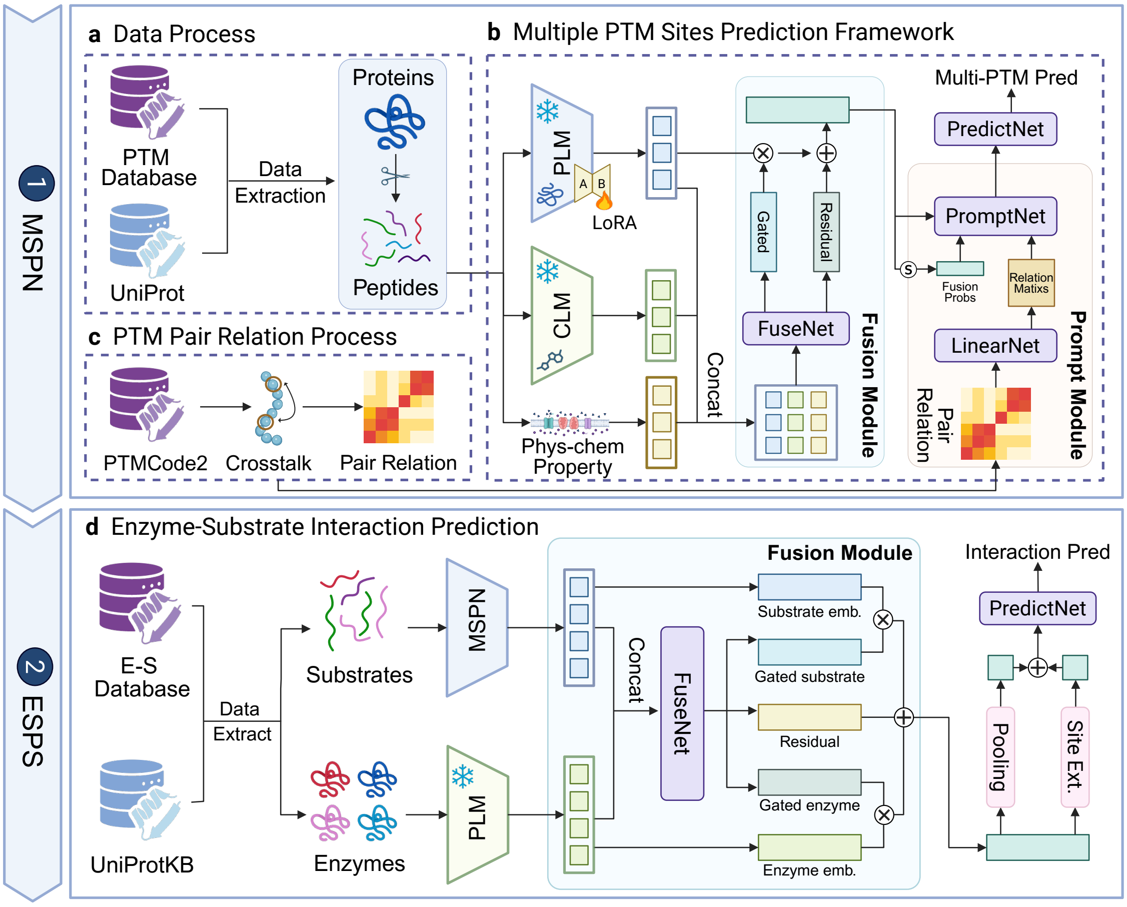

Supplementary Fig. 2: **Details of COMPASS-PTM. a-c**, Components of the first-stage Multi-label Site Profiling Network (MSPN). **a**, Data processing workflow showing extraction of PTM site annotations from curated PTM databases and full-length protein sequences from UniProt, followed by sequence segmentation into peptides. **b**, Multi-label PTM site prediction framework comprising protein language model (PLM) and chemical language model (CLM) encoders, LoRA-based fine-tuning, fusion modules for multimodal integration, and prompt-guided prediction networks that output site-specific PTM probabilities. **c**, PTM crosstalk relationship extraction from PTMCode2 database, processed into learnable pairwise relationship matrix that informs the prompt module for enhanced prediction accuracy. **d**, Second-stage Enzyme-Substrate Pairing System (ESPS) utilizing substrate and enzyme embeddings and fusion module to predict catalytic compatibility. The snowflake symbol indicates frozen model parameters, "×" denotes multiplicative interaction, "+" denotes additive fusion, "S" denotes activation function, and Site Ext. denotes site-centred feature extraction. Created in BioRender. Cao, H. (2026) https://BioRender.com/i9ugn6p.

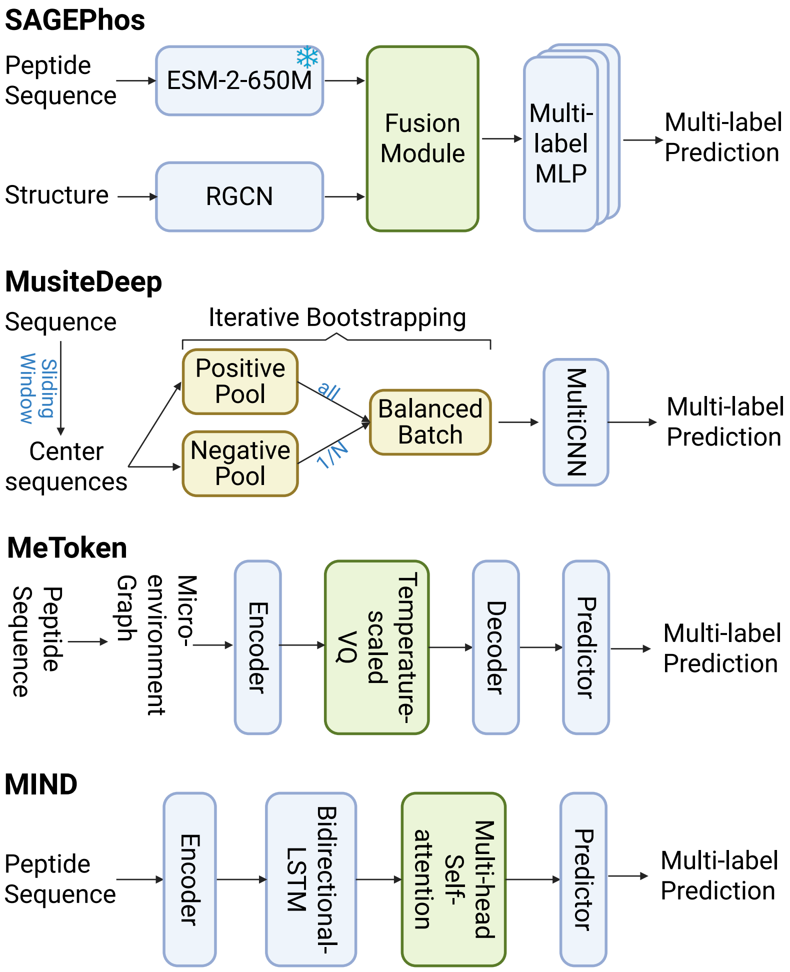

Supplementary Fig. 3: **Baseline model architectures.** Schematic overview of four representative baselines considered in this study: SAGEPhos, MusiteDeep, MeToken, and MIND-S. The snowflake symbol indicates frozen model parameters. Created in BioRender. Cao, H. (2026) https://BioRender.com/bqag6qy.

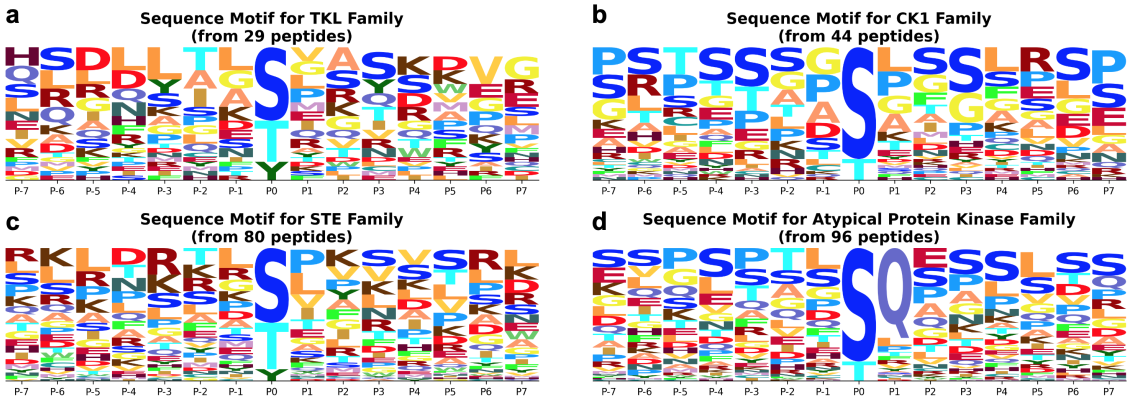

Supplementary Fig. 4: **Substrate Motif Recognition for Additional Kinase Families. a-d**, Sequence logos generated from high-confidence substrate peptides predicted by COMPASS-PTM for four additional kinase families: TKL (**a**, n=29 peptides), CK1 (**b**, n=44), STE (**c**, n=80), and Atypical protein kinases (**d**, n=96).

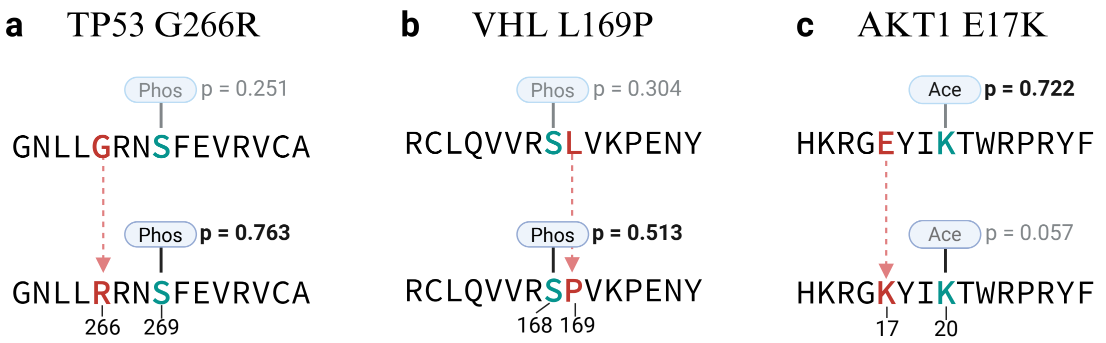

Supplementary Fig. 5: **More cases of predicting the PTM consequences of pathogenic variants.** a, The TP53 p.G266R cancer-associated substitution is predicted to induce a gain of phosphorylation at the proximal S269 site (predicted probability increases from 0.251 to 0.763). b, The VHL p.L169P variant, implicated in renal carcinoma, is predicted to cause a gain of phosphorylation at the adjacent S168 site (probability increases from 0.304 to 0.513). c, The AKT1 p.E17K oncogenic variant is predicted to result in a substantial loss of acetylation at the nearby K20 site (probability drops from 0.722 to 0.057). For each case, the diagrams illustrate the local sequence and predicted PTM probabilities, where the pathogenic substitution is highlighted in red and the affected PTM site is in green. Phos denotes phosphorylation, Ace denotes acetylation.

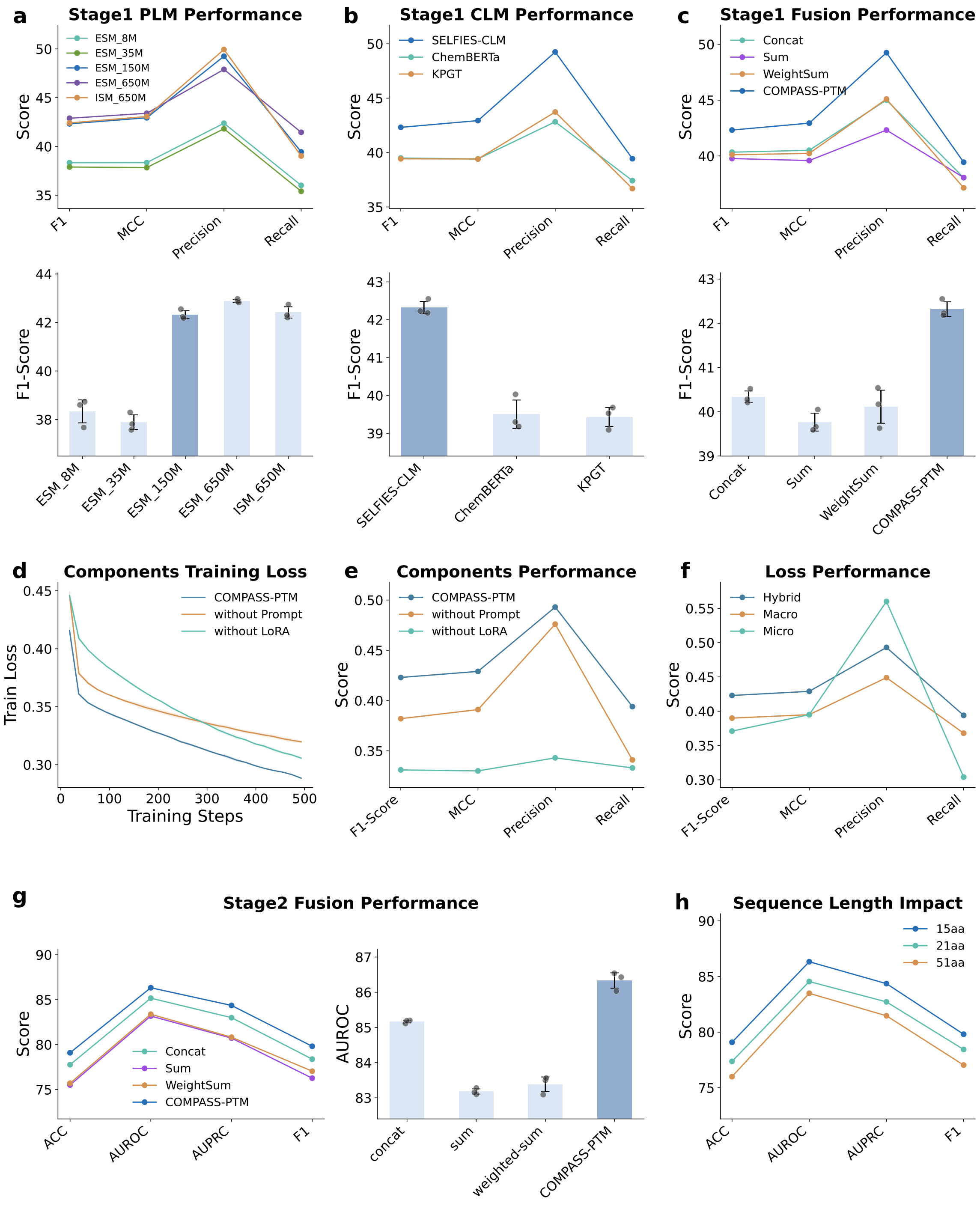

Supplementary Fig. 6: **Ablation Studies.** **a-c**, Empirical selection of suitable encoders and the initial fusion strategy at Stage 1. These panels present a comparative analysis to identify the suitable candidates for: (**a**) the Protein Language Model (PLM) encoder, (**b**) the Chemical Language Model (CLM) encoder, and (**c**) the feature fusion architecture. The results from these evaluations guide the component selection for the final model. **d**, **e**, Ablation studies investigating the contribution of key architectural components. The panels illustrate (**d**) the training loss curves and (**e**) the final performance metrics for the full model versus versions without prompt-tuning and without LoRA, demonstrating their essential role. **f**, Performance evaluation of the hybrid loss function against standard macro and micro averaging approaches. **g-h**, Selection of the suitable fusion architecture (**g**) and input peptide length (**h**) at Stage 2. For all bar charts, overlaid dots represent individual runs, and error bars indicate s.d. across $n = 3$ independent training runs with different random seeds. Source data are provided as a Source Data file.

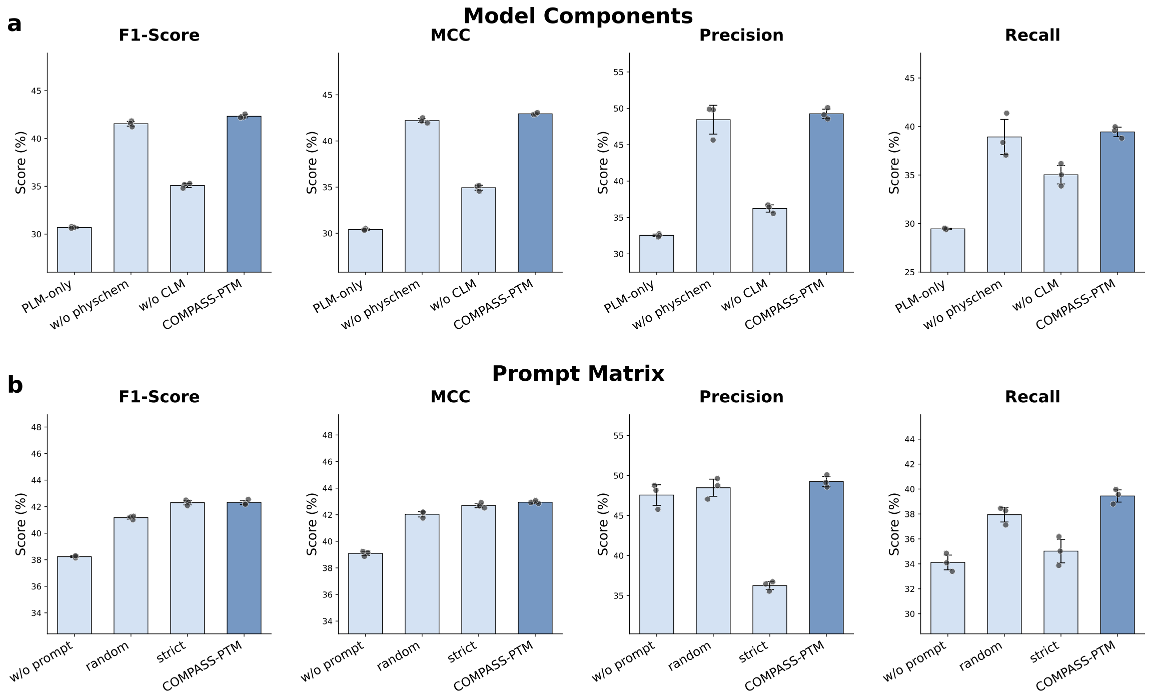

Supplementary Fig. 7: **Ablation studies of model components and prompt-matrix selection. a**, Model-component ablations: PLM-only, w/o physchem, w/o CLM, and COMPASS-PTM. **b**, Prompt-matrix ablations: w/o prompt, random, strict, and COMPASS-PTM. Bars show mean macro-F1, MCC, precision, and recall (in %; higher is better) under the same split and evaluation protocol. Overlaid dots represent individual runs; error bars indicate s.d. across $n = 3$ independent training runs with different random seeds. COMPASS-PTM is highlighted in dark blue. Source data are provided as a Source Data file.

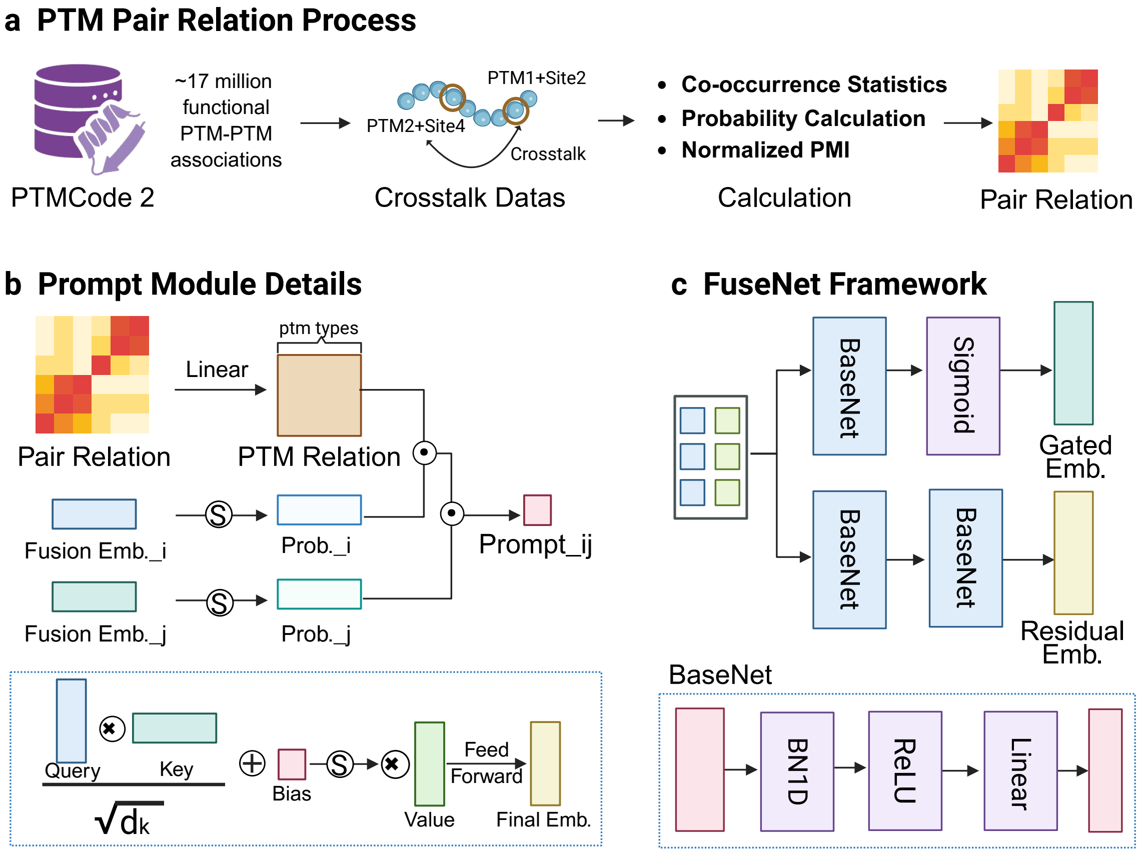

Supplementary Fig. 8: **Submodules of COMPASS-PTM.** **a**, Construction of the PTM relationship map. Approximately 17 million PTM-PTM functional associations from the PTMCode2 database [1] are processed using co-occurrence statistics, probability calculation and normalized pointwise mutual information (PMI) to generate a pairwise PTM relationship matrix. The heatmap represents the PTM relationship matrix, with colour intensity indicating relationship strength. **b**, Details of the prompt module. The module uses the relationship matrix from **a** to modulate predictions based on the likelihood of different PTM types co-occurring at the same site. "S" denotes activation function, "×" denotes multiplicative interaction, and "+" denotes bias addition. Emb. and Prob. denote embedding and predicted probability, respectively. In the scaled dot-product attention, d_k denotes the key dimension and $\sqrt{d_k}$ is the scaling factor. **c**, FuseNet framework. This module combines different input features using a gating mechanism to retain informative signals from each source. BN1D denotes one-dimensional batch normalization. BaseNet denotes the shared basic network block used in FuseNet. Created in BioRender. Cao, H. (2026) BioRender.com/feqiswk.

S.2 Supplementary Tables

Supplementary Table 1: **Program-level coherence metrics (SAGEPhos vs. COMPASS-PTM).** Spurious multi-label burden is reported in unconditional and conditional settings, and on all-sites and single-label residues ($|Y_i| = 1$).

Metric	Setting / Subset	SAGEPhos	COMPASS-PTM
SpuriousRate ↓	Unconditional (all sites)	0.7621	**0.0212**
	Unconditional ($|Y_i| = 1$)	0.7156	**0.0633**
	Conditional (all sites)	0.6541	**0.0957**
	Conditional ($|Y_i| = 1$)	0.6513	**0.0930**
AvgExtraLabels ↓	Unconditional (all sites)	1.7555	**1.0364**
	Unconditional ($|Y_i| = 1$)	1.4445	**1.1114**
	Conditional (all sites)	1.2993	**1.0677**
	Conditional ($|Y_i| = 1$)	1.2971	**1.0681**

Supplementary Table 2: Zero-shot performance on the DARKIN benchmark, evaluated using mean Average Precision (mAP). The best-performing model is highlighted in **bold**; the previous state-of-the-art is *italicized.*

Model	Parameters	mAP
Baseline Sequence Encoders		
ESM2 (avg)	650M	0.1391
ProtT5-XL	3B	0.1552
ESM1B (cls)	650M	0.1631
ESM1v (cls)	650M	0.1640
Encoder with Additional Kinase Features		
SaProt (cls) + Features	650M	0.1800
Encoder with Fine-tuning		
ProtT5-XL + Fine-tuning + Features	3B	0.1800
ESM1B (cls) + Fine-tuning + Features	650M	*0.1911*
**COMPASS-PTM (Ours)**	**150M**	**0.2946**

Supplementary Table 3: Predicted impact of 62 genetic variants on PTM sites. WT_Prob. and MT_Prob. represent the predicted probabilities for the wild-type and mutant sequences, respectively. Diff is the difference score(MT_Probab - WT_Probab). Abbreviations: Phos, Phosphorylation; Ubiq, Ubiquitylation; Meth, Methylation; SUMO, Sumoylation.

Gene	UniProt ID	WT	Pos.	Var	Mod. Pos.	PTM Type	WT Prob.	MT Prob.	Diff
APC	P25054	S	2621	C	2621	Phos	0.944	0.000	−0.944
HUWE1	Q7Z6Z7	K	4295	N	4295	Ubiq	0.869	0.000	−0.869
HEXA	P06865	K	342	R	342	Ubiq	0.820	0.000	−0.820
MAP2K1	Q02750	K	57	E	57	Ubiq	0.843	0.000	−0.843
USP8	P40818	S	718	P	718	Phos	0.931	0.000	−0.931
GIGYF2	Q6Y7W6	S	273	C	273	Phos	0.865	0.000	−0.865
MITF	O75030	E	425	K	423	SUMO	0.863	0.000	−0.862
SCN1A	P35498	S	525	F	525	Phos	0.869	0.000	−0.869
ACP1	P24666	S	137	F	137	Phos	0.867	0.000	−0.867
ACE2	Q9BYF1	K	26	R	26	Ubiq	0.855	0.000	−0.855
TNFRSF10A	O00220	K	443	N	443	Ubiq	0.815	0.000	−0.814
FLNC	Q14315	S	1624	L	1624	Phos	0.889	0.000	−0.889
ATP10D	Q9P241	T	43	I	43	Phos	0.880	0.000	−0.880
LRRFIP1	Q32MZ4	S	68	C	68	Phos	0.917	0.000	−0.917
ZNF592	Q92610	T	1024	N	1024	Phos	0.904	0.000	−0.904
HIP1R	O75146	K	404	Q	404	Ubiq	0.821	0.000	−0.821
WRN	Q14191	S	1133	A	1133	Phos	0.937	0.000	−0.937

Continued on next page

Supplementary Table 3: (Continued) Predicted impact of 62 genetic variants on PTM sites.

Gene	UniProt ID	WT	Pos.	Var	Mod. Pos.	PTM Type	WT Prob.	MT Prob.	Diff
TRIP11	Q15643	T	1846	I	1846	Phos	0.956	0.000	−0.956
RREB1	Q92766	S	1499	Y	1499	Phos	0.818	0.100	−0.717
MAP1B	P46821	S	1400	G	1400	Phos	0.889	0.000	−0.889
LATS1	O95835	S	204	G	204	Phos	0.919	0.000	−0.919
GAS2L1	Q99501	S	490	G	490	Phos	0.912	0.000	−0.912
PBRM1	Q86U86	K	231	R	231	Ubiq	0.895	0.000	−0.895
RBM20	Q5T481	S	637	G	637	Phos	0.952	0.001	−0.951
DHX38	Q92620	T	1217	A	1217	Phos	0.852	0.000	−0.852
KIF17	Q9P2E2	S	369	R	369	Phos	0.891	0.000	−0.891
KCNH2	Q12809	K	897	T	897	Phos	0.000	0.867	0.867
TRMT2A	Q8IZ69	S	602	R	602	Phos	0.843	0.000	−0.843
CLGN	O14967	S	579	F	579	Phos	0.808	0.000	−0.808
BRAF	P15056	S	467	A	467	Phos	0.906	0.000	−0.906
CDK13	Q14004	S	340	F	340	Phos	0.865	0.000	−0.865
FBXO4	Q9UKT5	S	12	L	12	Phos	0.917	0.000	−0.917
PKLR	P30613	R	359	C	354	Ubiq	0.824	0.125	−0.699
MYH9	P35579	K	910	Q	910	Ubiq	0.900	0.000	−0.900
SLC2A11	Q9BYW1	K	469	E	469	Ubiq	0.870	0.000	−0.869
BIN1	O00499	K	35	N	35	Ubiq	0.859	0.000	−0.859
SETX	Q7Z333	S	2612	G	2612	Phos	0.837	0.000	−0.837
MAP2K1	Q02750	K	57	N	57	Ubiq	0.843	0.001	−0.842
LCP2	Q13094	S	410	C	410	Phos	0.917	0.001	−0.916
CDK11B	P21127	S	414	L	414	Phos	0.907	0.000	−0.907
USP8	P40818	S	718	C	718	Phos	0.931	0.000	−0.931
DIABLO	Q9NR28	S	126	L	126	Phos	0.930	0.000	−0.930
ABL1	P00519	T	852	P	852	Phos	0.851	0.000	−0.851
RREB1	Q92766	S	1140	F	1140	Phos	0.905	0.000	−0.905
ST13P4	Q8IZP2	S	71	L	71	Phos	0.802	0.000	−0.802
CANT1	Q8WVQ1	S	303	R	303	Phos	0.867	0.000	−0.867
CDK13	Q14004	T	494	A	494	Phos	0.950	0.000	−0.950
LATS1	O95835	T	255	N	255	Phos	0.856	0.000	−0.856
FMR1	Q06787	R	546	H	546	Meth	0.980	0.000	−0.980
RAD50	Q92878	K	616	E	616	Ubiq	0.847	0.000	−0.847
BRCA2	P51587	T	582	P	581	Phos	0.154	0.863	0.709
MSH6	P52701	S	65	L	65	Phos	0.942	0.000	−0.942
SPATA31H1	Q68DN1	S	1665	P	1665	Phos	0.816	0.000	−0.816
VRK3	Q8IV63	S	59	F	59	Phos	0.889	0.000	−0.889
RAF1	P04049	T	310	A	310	Phos	0.856	0.000	−0.856
CERT1	Q9Y5P4	S	132	L	132	Phos	0.843	0.000	−0.843
PPP4R1	Q8TF05	S	593	N	593	Phos	0.882	0.000	−0.882
RAF1	P04049	S	259	F	259	Phos	0.872	0.000	−0.872
RAF1	P04049	S	259	A	259	Phos	0.872	0.000	−0.872
KIF16B	Q96L93	K	772	T	772	Ubiq	0.863	0.000	−0.863
RALA	P11233	K	128	R	128	Ubiq	0.931	0.000	−0.931
NCL	P19338	P	68	L	67	Phos	0.809	0.182	−0.627

Supplementary Table 4: **Protein-level overlap filtering statistics for the PTM-Code2 crosstalk source.** We performed protein-ID based de-duplication by removing PTMCode2 crosstalk records whose protein IDs overlap with the validation or test sets of our benchmarks. The remaining records were used to recompute the train-only, type-level NPMI prior ("strict" setting).

Benchmark	Split filtered	Filtering strategy	Original records	Removed (overlap)
dbPTM-ML	Test	Protein-level filtering	2,741,240	27,312
dbPTM-ML	Valid	Protein-level filtering	2,741,240	22,013
qPTM-ML	Test	Protein-level filtering	2,741,240	13,191
qPTM-ML	Valid	Protein-level filtering	2,741,240	11,550

S.3 Supplementary Notes

S.3.1 Datasets

In this two-stage process, we employ distinct datasets for different phases and tasks, as shown in Supplementary Fig. 1.

S.3.1.1 Preliminaries

dbPTM database [2], a long-standing integrated resource dedicated to Post Translational Modifications (PTMs) research, has been systematically maintained for over a decade to support functional and structural analyses of PTMs. The 2025 update of dbPTM represents a substantial expansion, now cataloging more than 2.79 million PTM sites across diverse proteomes. Of these, approximately 2.243 million sites are experimentally validated, curated from 48 established databases and over 80,000 peer-reviewed research articles through rigorous manual extraction. This extensive compilation ensures the database's utility as a foundational platform for investigating PTM-mediated regulatory mechanisms.

qPTM database [3] serves as a repository for quantitative PTM proteomics data, addressing the critical need for integrated analysis of reversible PTM dynamics across biological states. This resource expands upon the earlier qPhos framework to encompass six major PTM types — phosphorylation, acetylation, glycosylation, methylation, SUMOylation, and ubiquitylation — across four model organisms. Curated from 2,596 experimental conditions derived from 553 published studies, qPTM integrates 11,482,533 quantitative events mapping to 660,030 non-redundant PTM sites on 40,728 proteins, with matched global proteome data incorporated where available.

PTMint database [4] serves as a manually curated repository for experimentally validated PTM-mediated regulatory events on protein-protein interactions (PPIs). This resource specifically catalogs PTM sites that either enhance (increase affinity) or inhibit (decrease affinity) PPIs across six model organisms. Its inaugural release integrates 2,477 non-redundant PTM sites mapped to 1,169 proteins, which collectively modulate 2,371 protein-protein pairs implicated in 357 human diseases.

PTMCode 2 [1] is an integrative resource documenting functional associations between PTMs within and across protein complexes. It incorporates 316,546 experimentally verified PTM sites spanning 69 modification types across 19 eukaryotic species. Through orthology-based propagation, these annotations expand to $\geq$ 1.6 million predicted PTM sites, enabling the exploration of 17 million functional PTM-PTM associations involving $\geq 100,000$ proteins. This framework provides the most comprehensive landscape of context-specific PTM regulatory networks to date.

OmniPath [5] is a meta-database that integrates molecular prior knowledge from more than 100 publicly available resources to enable systems-level analysis of cellular regulation. Its core innovation lies in standardizing fragmented biological data into five interoperable modules: signaling networks, annotated protein complexes, multi-attribute protein annotations, intercellular communication roles, and critically—a comprehensive repository of curated enzyme–substrate relationships underlying diverse enzyme-mediated PTMs. Importantly, this PTM module is not restricted to kinases/phosphorylation: beyond kinase–substrate interactions, it also includes other enzymatic modification classes such as acetylation (acetyltransferases), methylation (methyltransferases), ubiquitination (E1/E2/E3 cascade), as shown in Supplementary Fig. 1 i. The PTM module, curated from 11 specialized resources, defines 39,201 enzyme–substrate interactions involving 1,821 enzymes and 16,467 substrate-specific modification sites across humans, mice, and rats.

UniprotKB [6] The UniProt Knowledgebase (UniProtKB) serves as a globally recognized resource for comprehensive protein annotation, integrating experimental and computationally inferred data to deliver accurate, standardized functional insights. Comprising two distinct sections, UniProtKB/Swiss-Prot provides manually curated records enriched with literature-derived evidence and expert-evaluated computational predictions, while UniProtKB/TrEMBL offers computationally analyzed entries awaiting full manual curation. The latest release of UniProtKB/Swiss-Prot encompasses 573,230 rigorously annotated protein entries, each detailing sequence information, taxonomic classification, functional roles, structural features, and disease associations.

SAGEPhos [7] dataset integrates phosphorylation sites from major repositories, including Phospho.ELM, PhosphoNetworks, and PhosphoSitePlus, augmented by structural data derived from AlphaFoldDB to enhance prediction accuracy. Following stringent quality control and redundancy removal, the dataset encompasses 18,360 high-confidence positive phosphorylation sites, representing a robust foundation for training and validating computational models.

S.3.1.2 MSPN datasets

In the first stage, we introduced three datasets for PTM site prediction: two novel multi-label datasets curated from dbPTM and qPTM, termed dbPTM-ML and qPTM-ML respectively, and one multi-class dataset derived from PTMint, designated as PTMint-MC.

dbPTM-ML To address the dual long-tail challenges inherent in multi-label PTM prediction—where both PTM type distribution and site-specific positive/negative sample ratios exhibit extreme skewness (Supplementary Fig. 1 b,h) —we implemented stringent data curation criteria. Only PTM types exceeding tens of thousands of experimentally documented sites were retained, ensuring statistically robust representation across both dominant and rare modification categories. This curation yielded seven high-confidence PTM categories with substantial experimental coverage: phosphorylation, acetylation, ubiquitylation, N-linked glycosylation, O-linked glycosylation, methylation, and succinylation, while collectively classifying all other modification types into a consolidated rare category to address long-tail distribution challenges. This process resulted in a dataset where a fraction of sites contains multiple PTMs (Supplementary Fig. 1c). As the dbPTM database lacks full-length protein sequences, we retrieved canonical sequences from UniProt [8] to contextualize PTM sites within native structural environments. To preserve local sequence contexts critical for PTM recognition while optimizing computational efficiency, we developed a greedy segmentation algorithm. Although conceptually analogous to sliding window approaches, our method fundamentally differs through its adaptive fragment selection mechanism: rather than fixed-step window sliding, the algorithm dynamically identifies optimal cleavage boundaries to generate peptides ≤ 50 aa that mandatorily contain ≥ 1 PTM site. This constraint guarantees functional relevance while eliminating non-informative segments, yielding a final curated repository of 1,577,125 PTM sites. We further mitigated overfitting risks using MMseqs2 [9] for sequence-space partitioning. Clustering at 40% sequence identity threshold generated non-redundant, phylogenetically independent subsets: 80% training, 10% validation, and 10% testing. This strategy minimizes bias from evolutionary correlations during model evaluation.

qPTM-ML dataset integrates all six PTM types documented in the qPTM database—phosphorylation, acetylation, ubiquitylation, glycosylation, methylation, and SUMOylation—with each modification type exhibiting robust experimental coverage (Supplementary Fig. 1d). Consequently, no rare-class designation was required. Identical data processing pipelines were implemented for peptide segmentation and sequence partitioning as described for dbPTM-ML, yielding a final curated repository of 662,072 PTM sites, whose multi-label distribution is detailed in Supplementary Fig. 1e.

PTMint-MC Given the limited scale of PTMint (2,477 non-redundant sites) and the near-absence of co-occurring PTM labels at identical sites, we exclusively allocated the entire dataset to a unified test cohort for standalone evaluation (Supplementary Fig. 1f, g). Processed through the same peptide segmentation pipeline as dbPTM-ML, these data were structured as a multi-class classification task, serving as an independent benchmark for model inference of context-specific PTM functional impacts.

S.3.1.3 ESPS datasets

For the second stage, we focused on the Enzyme-Substrate Pairing (ESP) task. To this end, we constructed and utilized two distinct datasets to train and evaluate the model's ability to predict specific enzyme-substrate interactions. Both datasets are structured with peptides centered on the modification site.

Our primary dataset was constructed using enzyme-substrate relationships curated from the OmniPath database [5], with full-length protein sequences for both enzymes and substrates retrieved from UniProt. For this dataset, substrate sequences were processed into 15-mer peptides centered on the modification site. To rigorously assess the model's performance and generalization capabilities, we designed three distinct evaluation settings: a warm-start scenario and two cold-start scenarios. In the warm-start setting, both enzymes and substrate peptides could appear in both the training and test sets, evaluating the model's performance on familiar data. To test generalization to novel entities, we implemented two stringent cold-start splits: a substrate cold-start setting, where all substrate peptides in the test set were strictly excluded from the training set, and an enzyme cold-start setting, where all enzymes present in the test set were entirely absent from the training set. For all settings, negative samples were generated by selecting unmodified residues of the same amino

acid type from within the same substrate peptide, maintaining a balanced 1:1 positive-to-negative sample ratio.

For comprehensive validation, we also utilized the established SAGEPhos benchmark dataset [7]. This dataset provides full-length kinase sequences paired with 11-mer substrate peptides, where the central residue represents the potential phosphorylation site.

The use of these two complementary datasets, with their differing sequence lengths and curation strategies, allows for a thorough and robust assessment of our model's ability to learn the molecular recognition patterns that govern enzyme-substrate specificity.

S.3.2 Evaluation Metric

To comprehensively evaluate the performance of our proposed model, we employed a diverse set of metrics that capture different aspects of classification quality and predictive capability.

Accuracy measures the proportion of correctly predicted instances, including both true positives and true negatives, among all predictions. It is calculated as

$$\text{Accuracy} = \frac{\text{TP} + \text{TN}}{\text{TP} + \text{TN} + \text{FP} + \text{FN}} \tag{S1}$$

where TP (true positives) represents correctly identified positive instances, TN (true negatives) represents correctly identified negative instances, FP (false positives) represents negative instances incorrectly classified as positive, and FN (false negatives) represents positive instances incorrectly classified as negative. While straightforward, accuracy alone may not be informative in imbalanced datasets.

Precision, also known as positive predictive value, quantifies the proportion of true positive predictions among all positive predictions, reflecting the model's ability to avoid false positives. It is defined as

$$\text{Precision} = \frac{\text{TP}}{\text{TP} + \text{FP}} \tag{S2}$$

which measures how many of the samples predicted as positive are actually positive.

Recall, also known as sensitivity, measures the proportion of actual positive instances correctly identified by the model, indicating its ability to detect PTM sites without missing true positives. It is calculated as

$$\text{Recall} = \frac{\text{TP}}{\text{TP} + \text{FN}} \tag{S3}$$

quantifying how completely the model captures all positive instances in the dataset.

The F1 score represents the harmonic mean of precision and recall, providing a balanced measure that is particularly useful for evaluating performance on imbalanced datasets where positive PTM sites are significantly fewer than negative sites. It is defined as

$$\text{F1} = 2 \times \frac{\text{Precision} \times \text{Recall}}{\text{Precision} + \text{Recall}} \tag{S4}$$

thereby balancing the trade-off between precision and recall.

The Matthews correlation coefficient, denoted MCC, serves as a balanced measure that considers all four confusion-matrix categories. It is calculated as

$$\text{MCC} = \frac{\text{TP} \times \text{TN} - \text{FP} \times \text{FN}}{\sqrt{(\text{TP} + \text{FP})(\text{TP} + \text{FN})(\text{TN} + \text{FP})(\text{TN} + \text{FN})}} \tag{S5}$$

producing a value between -1 and $+1$, where $+1$ indicates perfect prediction, 0 indicates random prediction, and -1 indicates complete disagreement between prediction and observation. MCC is especially valuable for evaluating binary classifiers on imbalanced datasets because it remains informative even when class distributions vary significantly.

The area under the receiver operating characteristic curve, denoted AUC-ROC, evaluates the model's ability to discriminate between positive and negative classes across various classification thresholds. The ROC curve plots the true positive rate, TPR, against the false positive rate, FPR, at different threshold settings:

$$\text{TPR} = \frac{\text{TP}}{\text{TP} + \text{FN}} \tag{S6}$$

$$\mathrm{FPR} = \frac{\mathrm{FP}}{\mathrm{FP} + \mathrm{TN}} \tag{S7}$$

AUC-ROC values range from 0 to 1, with higher values indicating better discrimination capability. This metric is particularly valuable because it is relatively insensitive to class imbalance and provides a comprehensive assessment of model performance across all possible decision thresholds.

The area under the precision-recall curve, denoted AUC-PRC, evaluates the trade-off between precision and recall across all thresholds. The precision-recall curve plots precision against recall, where recall is equivalent to TPR. Like AUC-ROC, its value ranges from 0 to 1, with higher scores indicating better performance.

These metrics collectively provide a comprehensive evaluation framework, allowing us to assess both the overall prediction accuracy and the model's performance in correctly identifying the typically underrepresented positive PTM sites.

S.3.3 Network Architecture

Our COMPASS-PTM model is built upon a dual-modal architecture that integrates evolutionary, structural, and chemical information to predict Post-Translational Modification (PTM) sites, as shown in Supplementary Fig. 2

S.3.3.1 Dual-Modal Encoders and Feature Representation

The model foundation consists of two complementary language models. For biological context, we use a Protein Language Model (PLM) based on ESM-2 (esm2_t30_150M_UR50D) [10], which features 30 transformer layers, 20 attention heads per layer, and produces 640-dimensional embeddings. To capture the chemical properties of amino acids, we employ a Chemical Language Model (CLM) operating on SELFIES-based molecular representations, which produces 320-dimensional chemical embeddings. In addition to these learned representations, we incorporate explicit physicochemical features, which provides a 4-dimensional vector for each amino acid encoding its molecular weight, isoelectric point, hydrophobicity, and polarity. To efficiently adapt the powerful PLM to the PTM prediction task, we employ Low-Rank Adaptation (LoRA) [11]. We apply LoRA with a rank of $r = 16$ and a scaling factor of $\alpha = 16$ to the query, key, and value matrices of the PLM's attention layers. A dropout rate of 0.1 is used within the LoRA modules to prevent overfitting. This strategy allows for efficient fine-tuning while keeping the base PLM's parameters frozen, preventing catastrophic forgetting (Supplementary Fig. 2b).

S.3.3.2 Crosstalk-Aware Prompting and Feature Fusion

The outputs from the PLM, CLM, and physicochemical features are first combined by a fusion network into a unified 640-dimensional representation. This fused embedding is then processed by two specialized transformer layers designed to model PTM interdependencies via crosstalk-aware prompting. Our custom attention mechanism within these layers first generates preliminary PTM probabilities via a softmax-activated head. Concurrently, a crosstalk prior matrix (Supplementary Fig.2c) is transformed through two learnable linear projections. An attention bias is then computed by modeling the interaction between the PTM probabilities and the transformed crosstalk matrix. This bias is added to the standard scaled dot-product attention scores, effectively steering the model's attention towards biochemically plausible PTM relationships.

S.3.3.3 Classification Head and Training Details

The final representation is passed to a Residual MLP classification head, which outputs logits for the 8 PTM types. The model was trained using the Adam optimizer [12] (learning rate 2×10^{-5}). We used a batch size of 256, and trained for a maximum of 100 epochs. These hyperparameters were uniformly applied across all model variations, including different ESM2 backbones (8M, 35M, 150M, and 650M). All experiments were conducted on a single NVIDIA A800 GPU.

S.3.4 Details of Baseline Adaptations

To ensure a fair and reproducible comparison under residue-level multi-label PTM site prediction, we evaluated MSPN against four representative baselines: SAGEPhos [7], MusiteDeep [13], MeToken

[14], and MIND-S [15]. For all baselines, we used the same train/validation/test splits as COMPASS-PTM on each benchmark (dbPTM-ML and qPTM-ML), and we report results using an identical evaluation pipeline (macro-averaged metrics; Supplementary Information S.3.2). When a baseline's original formulation did not natively match the multi-label setting (multiple PTM types may co-occur at the same residue), we applied the minimal architectural modifications necessary to align the output space and supervision with the benchmark label set while preserving the core encoder design. The baseline model architectures are shown in Supplementary Fig. 3.

SAGEPhos (adapted to multi-label prediction).

SAGEPhos was originally for an enzyme–substrate association formulation. For the substrate-level multi-label PTM task in this manuscript, we adapted SAGEPhos to predict PTM types at each residue by replacing the original prediction head with a multi-label MLP that outputs one score per PTM type. The upstream representation learning components (sequence encoder based on ESM2 and the fusion module; with an optional structural branch when applicable) were kept consistent with the baseline architecture, and the adapted model was retrained from scratch on our training split.

MusiteDeep (binary-to-multi-label head adaptation).

MusiteDeep is historically formulated as independent binary predictors. To align it with the multi-label benchmark setting, we expanded its output layer to produce C outputs corresponding to the benchmark PTM label set. The remainder of the architecture, including its CNN backbone and iterative bootstrapping strategy, was preserved. The adapted MusiteDeep was retrained from scratch using our training split and selected using the same validation protocol.

MeToken (codebook assignment adapted for PTM co-occurrence).

MeToken employs a vector-quantized (VQ) codebook and an encoder–decoder objective, where the original quantization step typically enforces a near-hard assignment of each input to a single codeword. In multi-label PTM prediction, however, multiple PTM types can co-occur at the same residue, which benefits from representations that can simultaneously express multiple discrete factors. To accommodate our benchmark setting, we apply a minimal adaptation by softening the codebook assignment via temperature scaling, allowing an input representation to be expressed as a mixture over multiple codewords rather than collapsing to a single discrete token. We retrain the model from scratch on the same training split with model selection on the same validation split.

MIND-S (native multi-label baseline).

MIND-S is natively designed for multi-label prediction (BiLSTM encoder with multi-head self-attention and a multi-label predictor). We kept the original architecture intact and retrained it from scratch on our training split to obtain a dataset-matched baseline under the same evaluation protocol.

Training and evaluation consistency.

Across all baselines, the final reported numbers were computed on the held-out test set using the same metric implementation (macro-F1, macro-MCC, etc.). This ensures that the observed improvements reflect differences in modeling assumptions and representational capacity rather than mismatched label spaces or evaluation procedures.

PTM-Mamba (ESPS comparison baseline).

Beyond residue-level PTM site prediction, our study also evaluates enzyme–substrate pairing (ESPS) on OmniPath (Stage 2). Since ESPS relies on a substrate sequence representation as input to the pairing model, we additionally compare against PTM-Mamba [16], a recent work that learns PTM-aware sequence embeddings via explicit proteoform tokenization. In PTM-Mamba, modified residues are treated as distinct tokens and the model is trained over PTM-modified proteoforms, producing embeddings that explicitly reflect the presence and type of modifications. In contrast, COMPASS-PTM operates on wild-type sequences only: Stage 1 learns an implicit, multi-label PTM program with crosstalk-aware prompting, and Stage 2 uses this program-oriented substrate representation for downstream ESPS. To compare representations without conflating differences in downstream

architecture, we use a controlled substitution strategy on OmniPath benchmarks: we keep the entire ESPS pipeline identical to COMPASS-PTM and only replace the substrate encoder (MSPN) with PTM-Mamba. All other components (pairing head, loss, optimizer, training schedule, and data splits and so on) remain unchanged.

S.3.5 Detailed Protein-level PTM program coherence metrics

Residue-wise metrics (macro-F1/MCC) quantify per-residue correctness but do not directly assess whether the predicted PTM label sets are coherent. In multi-label PTM site prediction, a frequent failure mode is over-calling: after localizing a modified residue, the model assigns additional PTM types beyond the curated label set, which can artificially inflate apparent PTM co-occurrence and weaken protein-level "PTM program" interpretation. To quantify this behavior, we introduce complementary program-level coherence metrics and report them in Supplementary Table 1.

S.3.5.1 Evaluation Metrics

Notation and thresholding.

For residue i, let Y_i denote the ground-truth PTM label set and $\widehat{Y}_i$ the predicted set after thresholding. For a fair comparison, we apply the same thresholding strategy used in the main benchmark to all methods, including COMPASS-PTM and all baselines, without method-specific tuning beyond the validation split.

Spurious multi-label burden.

We define a residue as spurious if it contains any predicted PTM labels that are not present in the ground truth:

$$\text{Spurious}(i) = \mathbb{I}\Big[\widehat{Y}_i \setminus Y_i \neq \emptyset\Big]. \tag{S8}$$

We report three related quantities: (i) an unconditional spurious rate over all evaluated residues, (ii) a conditional spurious rate restricted to residues for which at least one true PTM label is correctly recovered, and (iii) the average number of extra labels per spurious residue:

$$\text{SpuriousRate}_{\text{uncond}} = \frac{1}{N}\sum_{i=1}^{N}\text{Spurious}(i), \tag{S9}$$

$$\text{SpuriousRate}_{\text{cond}} = \frac{1}{|\mathcal{S}|}\sum_{i\in\mathcal{S}}\text{Spurious}(i), \quad \mathcal{S} = \Big\{i \mid \widehat{Y}_i \cap Y_i \neq \emptyset\Big\}, \tag{S10}$$

$$\text{AvgExtraLabels} = \mathbb{E}\Big[\Big|\widehat{Y}_i \setminus Y_i\Big| \;\Big|\; \widehat{Y}_i \setminus Y_i \neq \emptyset\Big]. \tag{S11}$$

We additionally report results on the single-label subset $\{i \mid |Y_i| = 1\}$ to isolate unambiguous coherence cases, in which extra PTM types are particularly undesirable.

Protein-level event-F1. To connect residue-level sets to protein-level "programs", for each protein p we form an event set $E_p = \{(i,t) \mid t \in Y_i\}$ and a predicted event set $\widehat{E}_p = \{(i,t) \mid t \in \widehat{Y}_i\}$, compute an event-level F1 per protein, and macro-average across proteins.

S.3.5.2 Analysis and interpretation.

Supplementary Table 1 shows that COMPASS-PTM produces more coherent multi-label outputs than SAGEPhos, beyond the improvements observed in residue-wise macro-F1/MCC.

First, COMPASS-PTM substantially reduces the frequency of spurious over-calling. Under the unconditional evaluation, which reflects end-to-end proteome-scale scanning, the spurious rate decreases from 0.7621 (SAGEPhos) to 0.0212 (COMPASS-PTM) on all sites, and from 0.7156 to 0.0633 on the single-label subset. This suggests that COMPASS-PTM does not generate widespread unsupported co-labeling when scanning full protein sequences. The advantage remains clear under the conditional evaluation, which isolates set-level coherence once at least one true PTM label is recovered at a residue: the spurious rate further decreases from 0.6541 to 0.0957 on all sites, and from 0.6513 to 0.0930 on the single-label subset. Together, these results indicate that COMPASS-PTM is not only better at localizing modified residues, but also more restrained in deciding which PTM types to assign once a site is recognized as modified.

Second, COMPASS-PTM also reduces the severity of spurious errors when they occur. Across all settings, AvgExtraLabels is consistently lower for COMPASS-PTM (approximately 1.04–1.11) than for SAGEPhos (approximately 1.30–1.76), meaning that SAGEPhos more often produces multiple extra PTM types at the same residue, whereas COMPASS-PTM's spurious errors are typically limited to a single additional label. This difference is important because excessive multi-label over-calling can inflate apparent residue-level co-occurrence and distort protein-level PTM patterns.

Third, the single-label subset ($|Y_i| = 1$) provides an especially clear coherence diagnostic. When the curated annotation indicates exactly one PTM type at a residue, additional predicted PTM types are particularly likely to be unsupported. COMPASS-PTM maintains both low spurious rates and low AvgExtraLabels in this subset, supporting that the model does not rely on over-expanded multi-label predictions to achieve residue-wise recall.

Finally, we complement these residue-set coherence analyses with a protein-level event F1 score, computed as a macro-average over proteins. This metric evaluates each protein as a structured set of residue–PTM events. On the test set, COMPASS-PTM achieves a mean protein-level F1 of 0.6430, indicating that the predicted PTM event composition of each protein is aligned with the ground-truth program, rather than being accurate only in an averaged residue-wise sense.

S.3.6 Detailed Cross-Task Benchmark Against PTMGPT2

As stated in the main text, to evaluate the generalizability of the principles embodied in our architecture, we conducted a cross-task benchmark against PTMGPT2, a strong single-PTM predictor. For the five binary classification datasets used in the PTMGPT2 study, we retrained MSPN from scratch using only the corresponding dataset, without carrying over weights or performing task-specific fine-tuning. This deliberately disadvantageous setting was designed to probe the robustness of the learned representations under varied data distributions.

Despite the differences in training objectives, the results show that COMPASS-PTM generalizes well across these tasks (Fig. 2d). Across all five datasets, the model achieved more balanced and accurate performance profiles. For example, on lysine acetylation, COMPASS-PTM achieved an F1 score of 0.800 and an MCC of 0.461, exceeding the baseline scores of 0.406 and 0.221 by 96.8% and 108.6%, respectively. Strong performance was also observed on lysine hydroxylation, a relatively rare modification with distinct biochemical properties, where MSPN achieved an F1 score of 0.885, representing a 29.8% improvement over PTMGPT2.

Overall, these results suggest that COMPASS-PTM learns representations that transfer across related PTM prediction tasks, rather than depending primarily on task-specific correlations. This supports the robustness and generalizability of the architecture in cross-task PTM prediction settings.

S.3.7 Detailed Zero-Shot Study to Unseen Kinases

S.3.7.1 Evaluation Metrics

The model's performance on the zero-shot kinase-substrate prediction task was quantitatively assessed using the mean average precision, denoted mAP, which measures the model's ability to rank true positive kinases above negative candidates across all substrates in the test set.

For a dataset comprising N substrates, mAP is defined as the mean of the average precision scores computed for individual substrates:

$$\mathrm{mAP} = \frac{1}{N} \sum_{i=1}^{N} \mathrm{AP}_i \tag{S12}$$

where AP_i is the average precision for the i-th substrate.

For a single substrate s, the average precision is computed from the ranked list of all K candidate kinases, ordered by the model's predicted interaction scores. It is defined as the sum of the precision values at those ranks corresponding to true positive kinases, normalized by the total number of true positive kinases for that substrate:

$$\mathrm{AP}_s = \frac{1}{|\mathcal{K}_s^+|} \sum_{k=1}^{K} \mathrm{Prec}(k)\, \mathbb{I}\big(\mathrm{rank}_k(s) \in \mathcal{K}_s^+\big) \tag{S13}$$

Here, $\mathcal{K}_s^+$ denotes the set of true positive kinases for substrate s, and $|\mathcal{K}_s^+|$ denotes its cardinality. The term $\text{rank}_k(s)$ denotes the kinase at rank k in the predicted list. The indicator function $\mathbb{I}(\cdot)$ takes value 1 if the kinase at rank k is a true positive and 0 otherwise.

The precision at rank k, denoted $\text{Prec}(k)$, measures the fraction of true positives among the top-k predictions:

$$\text{Prec}(k) = \frac{1}{k}\sum_{j=1}^{k} \mathbb{I}\big(\text{rank}_j(s) \in \mathcal{K}_s^+\big) \tag{S14}$$

This formulation ensures that the average precision rewards models that not only retrieve correct kinases but also place them at higher ranks in the prediction list, thereby providing a robust evaluation of generalization in the zero-shot setting.

S.3.7.2 Performance Comparison on the DARKIN Benchmark

To rigorously assess the zero-shot generalization capability of our model, we evaluated it on the DARKIN benchmark[17]. The DARKIN benchmark is a zero-shot learning framework designed to evaluate a model's ability to associate phosphosites with previously unseen "dark kinases"—kinases that are understudied. This task is of significant biological importance, as experimentally identifying the specific kinase for over 95% of known phosphosites remains a major challenge , and kinases are crucial drug targets in many diseases

The benchmark's integrity is ensured through a carefully designed data splitting strategy. It enforces strictly disjoint sets of kinases between its training, validation, and testing splits. Furthermore, to prevent overly optimistic results and ensure a true zero-shot evaluation, the splits are stratified by kinase groups and kinases with high sequence similarity are exclusively assigned to the same set. Performance is measured using macro Average Precision (mAP), a metric chosen for its stability and robustness against class imbalance, which is common in this problem.

We compare our model, COMPASS-PTM, against the top-performing protein language model encoders and fine-tuning strategies reported in the original DARKIN study. As shown in Supplementary Table 2, COMPASS-PTM substantially outperforms all prior methods. The previous state-of-the-art performance on this challenging benchmark was an mAP of 0.1911, achieved by a fine-tuned ESM1B model that was augmented with explicit biological features (kinase family, group, and EC information). In stark contrast, our model, COMPASS-PTM, achieves a new state-of-the-art mAP of 0.2946, surpassing the best baseline by a remarkable 54%.

Notably, our model achieves this superior performance while using a significantly more parameter-efficient ESM2-150M backbone. This result is particularly significant given that the DARKIN study itself concludes that dark kinase prediction remains a "highly challenging task" for current protein language models. The substantial performance gain underscores the effectiveness of COMPASS-PTM's architecture, which learns representations highly informative for kinase-substrate specificity through an initial stage dedicated to learning PTM-specific codes.

S.3.8 Substrate Motif Recognition for Additional Kinase Families

Our analysis of the remaining kinase families revealed that the model successfully recovered a diverse range of canonical motifs, each with distinct biochemical features. For the TKL (Tyrosine Kinase-Like) Family, a diverse group that structurally resembles tyrosine kinases but phosphorylates serine/threonine, the resulting logo reflects the known diversity of this group; while lacking a single, highly constrained consensus, the model successfully captured several subtle but significant preferences, including an enrichment for basic residues at P-2 and small, non-polar residues at P+1, showing it is capable of learning weaker sequence patterns from functionally diverse enzyme families (Supplementary Fig. 4a). The model's capacity to learn complex signatures was further evidenced by the logo for the CK1 (Casein Kinase 1) Family, a highly conserved group of acidophilic kinases involved in numerous cellular processes, which perfectly captures its hallmark feature: a strong preference for a Serine (S) or Threonine (T) at the P-3 position. This reflects the well-established primed acidophilic motif (S/T-X-X-S/T) for CK1, where an upstream phosphorylation event is often required for substrate recognition (Supplementary Fig. 4b). For the STE (Sterile Homolog) family, which constitutes a major part of the mitogen-activated protein kinase (MAPK) signaling cascades, the model captured a multi-faceted recognition motif. The most dominant feature identified is the stringent requirement for a Proline (P) at the P+1 position, which defines the canonical proline-directed signature (S/T-P) essential for MAPK signaling. Additionally, it captured a significant

secondary signal for basic amino acids, especially Lysine (K), at the P-2 position, showcasing its ability to learn complex sequence patterns (Supplementary Fig. 4c). Finally, for the Atypical Protein Kinase Family, a functionally diverse group that lacks sequence homology to the main protein kinase superfamily, the model accurately learned the Ł-S/T-Q consensus motif, which is the signature of the clinically significant PIKK subgroup (including mTOR, ATM, and ATR). This highlights the model's ability to capture the critical preference for a Leucine (L) at P-1 and a Glutamine (Q) at P+1 that governs recognition by these kinases (Supplementary Fig. 4d).

S.3.9 More Cases for Predicting PTM Alterations Driven by Pathogenic Mutations

In addition to the cases presented in the main text, we further highlight COMPASS-PTM's utility by examining several cancer-associated somatic mutations whose functional impacts have recently been computationally validated by the DeepMVP study [18].

For the TP53 p.G266R loss-of-function variant, COMPASS-PTM generated the novel hypothesis that the mutation induces a gain of phosphorylation at S269 (Supplementary Fig. 5a). This offers a compelling explanation for the variant's pathogenic effect, as S269 phosphorylation is reported to inhibit TP53 activity. Similarly, for the AKT1 p.E17K hotspot substitution, a known driver of oncogenic activation, our model predicted a substantial loss of acetylation at the adjacent K20 site (Supplementary Fig. 5c). This prediction aligns perfectly with experimental evidence that K20 acetylation inhibits AKT1 activity, thus providing a direct mechanistic link where the E17K variant activates the kinase by reducing this inhibitory modification. Furthermore, the model proposed a functional consequence for the recurrent but uncharacterized VHL p.L169P variant, predicting a gain of phosphorylation at the S168 site (Supplementary Fig. 5b). This suggests the variant may contribute to tumorigenesis by promoting an S168 phosphorylation event that is known to mark the VHL tumor suppressor for ubiquitination and degradation.

These examples further underscore the utility of COMPASS-PTM in generating precise, testable hypotheses to elucidate the molecular mechanisms underlying pathogenic mutations.

S.3.10 Predicted PTM-Abolishing Variants in Sperm Proteins

To demonstrate the utility of COMPASS-PTM in elucidating disease mechanisms, we first performed a systematic screen to identify pathogenic mutations with a high likelihood of altering the post-translational code. To probe the genetic basis of reproductive pathology, a critical yet understudied field, we cross-referenced pathogenic variants from the PhosphoSitePlus PTMVar database [19] against a comprehensive catalog of the human sperm proteome [20]. By applying a stringent filtering criterion (wild-type prediction score > 0.8 and mutant prediction score < 0.2) to pinpoint mutations predicted to abolish PTM events, our screen identified 62 high-confidence SNPs (Supplementary Table 3).

S.3.11 Detailed Ablation Studies

To systematically validate the design principles of COMPASS-PTM and quantify the contribution of each architectural component, we conducted a series of ablation experiments. These studies evaluate the effectiveness of each designed module, providing a clear rationale for the methodological choices that drive the model's performance.

S.3.11.1 Dissecting the Stage 1 Multi-label Site Profiling Network (MSPN)

Optimizing the Dual-Modal Encoder Architecture. The MSPN's performance is built upon its dual-modal encoder; we therefore began by optimizing its core components. First, for the protein language model, selection experiments revealed that ESM2-150M [10] provides the optimal balance between representational power and efficiency. This model improved the F1-score and MCC by 11.6% and 13.5% respectively over smaller variants, while larger models offered diminishing returns, as shown in Supplementary Fig. 6a. Next, evaluation of the chemical language model variants (Supplementary Fig. 6b) showed that the SELFIES-based CLM used in our model outperformed alternatives (7.1% F1-score and 8.9% MCC improvement), validating our hypothesis that physicochemical reactivity patterns provide crucial information complementary to evolutionary context. Finally, our

proposed dual-modal fusion architecture proved substantially more effective than conventional methods, outperforming simple concatenation and standard attention by 4.7% in F1-score, as shown in Supplementary Fig. 6c. Its gated-residual design effectively treats evolutionary features as primary signals while selectively incorporating chemical information, a principle that enhances discriminative capacity.

Contribution of Crosstalk-Aware Prompting. The crosstalk-aware prompting module is a critical innovation for modeling PTM crosstalk. Ablation experiments (Supplementary Fig. 6d-e) quantify its substantial impact: its removal results in a 10.7% decrease in F1-score and a 9.8% decrease in MCC, accompanied by a degradation in both precision and recall. This confirms that the prompt module successfully resolves ambiguity among competing PTM labels by refining its learned PTM co-occurrence patterns, leading to predictions that better reflect biological reality.

Contribution of LoRA Fine-Tuning. To efficiently adapt the large-scale protein language model to the PTM prediction task, we employed Low-Rank Adaptation (LoRA) [11] for parameter-efficient fine-tuning. A direct comparison between models trained with and without LoRA reveals the profound impact of this strategy. Shown in Supplementary Fig. 6d-e, the use of LoRA boosted key performance metrics by approximately 30%, including a 44% improvement in precision. This underscores the necessity of parameter-efficient fine-tuning for effectively specializing large, generalist PLMs to a nuanced task like PTM profiling.

Efficacy of the Hybrid Loss Function. Our hybrid loss function was designed to address the challenging double long-tail distribution of PTM datasets. Ablation experiments confirmed that individual loss functions offer a suboptimal trade-off: Dice loss achieves high recall but low precision, while Focal loss delivers high precision but poor recall. By combining them, our hybrid approach successfully leverages their complementary strengths to achieve a balanced performance profile, culminating in a state-of-the-art F1-score (0.423) and MCC (0.429), as shown in Supplementary Fig. 6f. This ensures both sensitivity to rare modifications and specificity for confident functional annotation.

Model Component Ablations. To disentangle the contribution of each modality and the fusion pathway, we conducted architecture-level component ablations on dbPTM-ML under the same data split and evaluation protocol (Supplementary Fig. 7 **a**). We compare four settings: (i) PLM-only, which predicts PTM labels from ESM2-derived residue representations without any chemical branch; (ii) w/o CLM, which removes the CLM branch from the otherwise unchanged full pipeline; (iii) w/o physchem (PLM+CLM, no physchem), which enables the CLM branch and PLM–CLM fusion while removing explicit physicochemical descriptors; and (iv) COMPASS-PTM (Full), the complete model with PLM–CLM fusion and explicit physicochemical descriptors. We use the full model as the reference point and interpret each ablation by the degradation it induces relative to this reference. First, removing the chemical pathway entirely (PLM-only) causes a substantial performance drop relative to the full model, with macro-F1 falling from 42.32% to 30.69% (and similar declines across the other metrics). This indicates that sequence-only PLM representations, while informative, are insufficient for robust proteome-scale multi-label PTM profiling in our setting. Second, removing the CLM branch within the otherwise unchanged full pipeline (w/o CLM) also leads to a pronounced degradation, reducing macro-F1 from 42.32% to 35.09%. This within-configuration drop directly supports that the CLM stream contributes materially to the integrated architecture rather than acting as a redundant auxiliary branch. Third, removing only the explicit physicochemical descriptors while retaining PLM–CLM fusion (w/o physchem) produces a smaller but still measurable decline, with macro-F1 decreasing from 42.32% to 41.54%. Thus, although the principal gain comes from multimodal PLM–CLM integration, the explicit physicochemical descriptors still provide complementary refinement and help the full model achieve the best overall performance. In addition, we implemented two extreme backbone-removal controls: CLM-only and strict w/o ESM. Both perform very poorly (macro-F1 = 0.056 for CLM-only; macro-F1 = 0.159 for w/o ESM), confirming that the contextualized PLM backbone is essential. Overall, these ablations support our PLM-anchored dual-modal design: the main performance gain arises from integrating the chemical branch with the PLM backbone, while explicit physicochemical descriptors provide additional complementary refinement. Together, the results support genuine multimodal complementarity rather than an improvement attributable to PLM fine-tuning alone.

Prompt Matrix Ablations and Robustness to Provenance Overlap. The crosstalk-aware prompting module injects a PTM-type dependency bias into attention through a learnable interaction matrix R, which is initialized from an external crosstalk resource (PTMCode2) and then fine-tuned

end-to-end. A key question is whether the observed gain could be explained by circularity if the external resource overlaps with benchmark proteins. We note that R_{prior} is computed from aggregated PTM-type co-occurrence statistics and does not contain protein IDs, site indices, sequence windows, or other instance-resolved benchmark information. It therefore provides only a type-level prior and cannot specify the ground-truth label assignment for any individual benchmark example. To provide empirical support, we first rebuilt the PTMCode2-based prior after excluding all records associated with proteins appearing in the validation/test splits. The resulting strict prior remained almost unchanged relative to the original matrix (upper-triangular comparison: Pearson $r = 0.9993$, Spearman $\rho = 0.9929$, MAE $= 4.35 \times 10^{-4}$, RMSE $= 2.50 \times 10^{-3}$), indicating that the global PTM-type crosstalk structure is highly stable under protein-overlap filtering. We then compared four settings: (i) Original prior (COMPASS-PTM); (ii) Strict de-duplicated prior (train-only provenance control). To explicitly eliminate any potential provenance overlap at the protein level, we removed from the PTMCode2 crosstalk source all records associated with proteins appearing in the validation/test splits and recomputed the type-level NPMI matrix from the remaining records (Supplementary Table 4). Importantly, this control retains the same type-level formulation but ensures that the prior is derived from proteins disjoint from evaluation proteins. (iii) Random initialization (learnable but uninformative). We also replace the PTMCode2 initialization with a randomly initialized matrix of the same shape, which is still learnable during training. This controls for the possibility that the gain arises merely from adding extra parameters or a generic attention bias, rather than from meaningful crosstalk structure. (iv) No prompt (w/o prompt). Finally, we remove the prompting term entirely, providing a lower-bound reference where the model must learn multi-label dependence purely from sequence-conditioned supervision. As shown in Supplementary Fig. 7 **b**, the strict de-duplicated prior achieves performance that is essentially indistinguishable from the original COMPASS-PTM initialization across macro-F1/MCC/precision/recall, indicating that the observed gains are not driven by benchmark overlap. In addition, both strict and COMPASS-PTM consistently outperform the learnable but semantically uninformative random initialization, and all prompted variants outperform the w/o prompt setting. Collectively, these results support our intended interpretation: even after explicitly removing from PTMCode2 all crosstalk records associated with proteins in the validation/test splits and recomputing a strict prior, performance remains essentially unchanged, ruling out a shortcut "retrieval" explanation based on shared test-set PTM pairs. Rather, the improvement arises from injecting meaningful PTM-type dependency structure as a soft, learnable inductive bias, beyond what can be obtained from an unstructured (random) initialization.

S.3.11.2 Validating the Stage 2 Enzyme-Substrate Pairing System (ESPS)

Optimal Fusion Strategy. To validate the superiority of our dual gated-residual module for fusing enzyme and substrate features, we benchmarked its performance against standard strategies, including concatenation, summation, weighted summation, and a canonical cross-attention mechanism. Our proposed architecture's superior performance across all evaluated metrics (Supplementary Fig. 6g) validates our gating mechanism, which learns to selectively focus on the most informative features from both the enzyme and substrate to accurately model their interaction.

Optimal Input Peptide Length. To identify the optimal local sequence context for the pairing task, we compared model performance using substrate peptide lengths of 15aa, 21aa, and 51aa, representing a common baseline length, a recent standard utilized by the PTMGPT2 [21] model, and a length consistent with our Stage 1 analysis, respectively. The results clearly indicate that the 15aa peptide length yielded the highest performance, as shown in Supplementary Fig. 6h. This finding suggests that the core determinants of enzyme-substrate specificity are concentrated within the immediate flanking residues, making the 15aa length not only the empirically optimal choice but also the most biologically plausible, as it captures this critical local context while minimizing noise from distal sequences.

S.4 Supplementary Methods

S.4.1 Multi-label Site Profiling Network

S.4.1.1 Implementation of the Fusion Module

The Bio-Coupled and Augmented Fusion module (Supplementary Fig. 8c) operates on the primary protein representation, denoted $\mathbf{X}_p = \mathbf{H}_s^{\text{prot}}$, and the auxiliary chemical representation, $\mathbf{X}_a = \mathbf{H}_s^{\text{chem}}$.

The gating mechanism, denoted $\mathbf{\Phi}$, uses the auxiliary features to generate a dynamic gate that modulates the primary features. This process filters out potential noise from $\mathbf{X}_a$ and selectively applies useful information. It is formulated as

$$\mathbf{\Phi}(\mathbf{X}_p, \mathbf{X}_a) = \sigma(\mathbf{W}_{g2}\ \text{ReLU}(\mathbf{W}_{g1}[\mathbf{X}_p; \mathbf{X}_a])) \odot \mathbf{X}_p \tag{S15}$$

where $[\cdot;\cdot]$ denotes feature concatenation, σ is the sigmoid activation, $\odot$ is element-wise multiplication, and $\mathbf{W}_{g1}$ and $\mathbf{W}_{g2}$ are trainable weight matrices in a two-layer multilayer perceptron.

The residual mechanism, denoted $\mathbf{\Psi}$, learns a residual mapping from the concatenated features to capture cross-modal interactions that enrich the final representation. It is defined as

$$\mathbf{\Psi}(\mathbf{X}_p, \mathbf{X}_a) = \mathbf{W}_{r2}\ \text{ReLU}(\mathbf{W}_{r1}[\mathbf{X}_p; \mathbf{X}_a]) \tag{S16}$$

where $\mathbf{W}_{r1}$ and $\mathbf{W}_{r2}$ are the trainable weights of a separate two-layer multilayer perceptron.

S.4.1.2 Prompting PTM Attention Matrices

The foundation of this prompt is a prior relationship matrix, $\mathbf{R}_{\text{prior}}$, derived from global PTM co-occurrence statistics. We computed this matrix using normalized pointwise mutual information on over 17 million PTM associations from the PTMCode 2 database [1]. The resulting normalized pointwise mutual information scores, ranging from -1 (mutually exclusive) to $+1$ (co-occurring), populate $\mathbf{R}_{\text{prior}}$, which is defined as symmetric with a zero diagonal to isolate crosstalk effects (Supplementary Fig. 2c and Supplementary Fig. 8a).

To adapt the global statistics to task-specific patterns, the static prior $\mathbf{R}_{\text{prior}}$ is transformed into a learnable matrix $\mathbf{R}$ via a projection network g_θ:

$$\mathbf{R} = g_\theta(\mathbf{R}_{\text{prior}}) \tag{S17}$$

Specifically, g_θ projects $\mathbf{R}_{\text{prior}}$ using two parallel linear layers, and the resulting matrices are combined via matrix multiplication to produce $\mathbf{R}$. This architecture enables the model to learn complex, non-linear dependencies between PTM types, while initializing the learning process with a strong biological prior (Supplementary Fig. 8b).

S.4.1.3 Detailed Loss Function Formulation

The macro-averaged Dice loss, denoted $\mathcal{L}_{\text{Dice}}$, is computed as the macro-average of the Dice coefficient over all C classes. This treats each class equally, regardless of its prevalence. The formulation is

$$\mathcal{L}_{\text{Dice}} = 1 - \frac{1}{C}\sum_{c=1}^{C} \frac{2\sum_{i=1}^{N} y_i^{(c)} \hat{y}_i^{(c)} + \epsilon}{\sum_{i=1}^{N} y_i^{(c)} + \sum_{i=1}^{N} \hat{y}_i^{(c)} + \epsilon} \tag{S18}$$

where N is the total number of sites in a batch, C is the number of PTM classes, $y_i^{(c)} \in \{0, 1\}$ is the ground-truth label, and $\hat{y}_i^{(c)}$ is the predicted sigmoid probability for site i and class c. The smoothing factor ϵ prevents division by zero.

The micro focal loss, denoted $\mathcal{L}_{\text{micro}}$, addresses class imbalance by reducing the loss contribution from well-classified examples, allowing the model to focus on hard negatives and positives. It is defined as

$$\mathcal{L}_{\text{micro}} = -\frac{1}{N}\sum_{i=1}^{N}\sum_{c=1}^{C}\Big[y_i^{(c)}\big(1-\hat{p}_i^{(c)}\big)^{\gamma}\log\big(\hat{p}_i^{(c)}\big) + \big(1-y_i^{(c)}\big)\big(\hat{p}_i^{(c)}\big)^{\gamma}\log\big(1-\hat{p}_i^{(c)}\big)\Big] \tag{S19}$$

where $\hat{p}_i^{(c)}$ is the predicted probability for sample i and class c. The focusing parameter γ is set to 2.0 and controls the down-weighting effect.

S.4.2 Enzyme–Substrate Pairing System

S.4.2.1 Implementation Details of Dual-Gated Residual Fusion

Our dual-gated architecture symmetrically processes both substrate peptide and enzyme features as co-primary modalities. This is achieved through two reciprocal gating units, where each gate learns to dynamically refine the representation of the opposing modality, thereby removing noise and isolating interaction-specific signals. Concurrently, a residual connection preserves the holistic information from both feature streams, preventing information loss during the filtering process (Supplementary Fig. 2d).

Input features are concatenated as

$$\mathbf{H}_{\text{combined}} = [\mathbf{H}_{\text{substrate}}; \mathbf{H}_{\text{enzyme}}]. \tag{S20}$$

Two independent multilayer perceptrons generate modality-specific gates:

$$\mathbf{g}_{\text{substrate}} = \sigma(\text{MLP}_{\text{gate,sub}}(\mathbf{H}_{\text{combined}}))\,, \tag{S21}$$

$$\mathbf{g}_{\text{enzyme}} = \sigma(\text{MLP}_{\text{gate,enz}}(\mathbf{H}_{\text{combined}}))\,. \tag{S22}$$

A third multilayer perceptron learns interaction-specific features:

$$\mathbf{H}_{\text{residual}} = \text{MLP}_{\text{res}}(\mathbf{H}_{\text{combined}}) \tag{S23}$$

References

[1] Minguez, P., Letunic, I., Parca, L., Garcia-Alonso, L., Dopazo, J., Huerta-Cepas, J., Bork, P.: Ptmcode v2: a resource for functional associations of post-translational modifications within and between proteins. Nucleic acids research **43**(D1), 494–502 (2015)

[2] Chung, C.-R., Tang, Y., Chiu, Y.-P., Li, S., Hsieh, W.-K., Yao, L., Chiang, Y.-C., Pang, Y., Chen, G.-T., Chou, K.-C., *et al.*: dbptm 2025 update: comprehensive integration of ptms and proteomic data for advanced insights into cancer research. Nucleic Acids Research **53**(D1), 377–386 (2025)

[3] Yu, K., Wang, Y., Zheng, Y., Liu, Z., Zhang, Q., Wang, S., Zhao, Q., Zhang, X., Li, X., Xu, R.-H., *et al.*: qptm: an updated database for ptm dynamics in human, mouse, rat and yeast. Nucleic Acids Research **51**(D1), 479–487 (2023)

[4] Hong, X., Li, N., Lv, J., Zhang, Y., Li, J., Zhang, J., Chen, H.-F.: Ptmint database of experimentally verified ptm regulation on protein–protein interaction. Bioinformatics **39**(1), 823 (2023)

[5] Türei, D., Korcsmáros, T., Saez-Rodriguez, J.: Omnipath: guidelines and gateway for literature-curated signaling pathway resources. Nature methods **13**(12), 966–967 (2016)

[6] Boutet, E., Lieberherr, D., Tognolli, M., Schneider, M., Bansal, P., Bridge, A.J., Poux, S., Bougueleret, L., Xenarios, I.: Uniprotkb/swiss-prot, the manually annotated section of the uniprot knowledgebase: how to use the entry view. Plant bioinformatics: methods and protocols, 23–54 (2016)

[7] Zhang, J., Hanqun, C., Gao, Z., Wang, X., Gu, C.: Sagephos: Sage bio-coupled and augmented fusion for phosphorylation site detection. In: The Thirteenth International Conference on Learning Representations

[8] Uniprot: the universal protein knowledgebase in 2023. Nucleic acids research **51**(D1), 523–531 (2023)

[9] Steinegger, M., Söding, J.: Mmseqs2 enables sensitive protein sequence searching for the analysis of massive data sets. Nature biotechnology **35**(11), 1026–1028 (2017)

[10] Lin, Z., Akin, H., Rao, R., Hie, B., Zhu, Z., Lu, W., Smetanin, N., Verkuil, R., Kabeli, O., Shmueli, Y., *et al.*: Evolutionary-scale prediction of atomic-level protein structure with a language model. Science **379**(6637), 1123–1130 (2023)

[11] Hu, E.J., Shen, Y., Wallis, P., Allen-Zhu, Z., Li, Y., Wang, S., Wang, L., Chen, W., *et al.*: Lora: Low-rank adaptation of large language models. ICLR **1**(2), 3 (2022)

[12] Kingma, D.P.: Adam: A method for stochastic optimization. arXiv preprint arXiv:1412.6980 (2014)

[13] Wang, D., Liu, D., Yuchi, J., He, F., Jiang, Y., Cai, S., Li, J., Xu, D.: Musitedeep: a deep-learning based webserver for protein post-translational modification site prediction and visualization. Nucleic Acids Research **48**(W1), 140–146 (2020)

[14] Tan, C., Cao, Z., Gao, Z., Wu, L., Li, S., Huang, Y., Xia, J., Hu, B., Li, S.Z.: Metoken: Uniform micro-environment token boosts post-translational modification prediction. arXiv preprint arXiv:2411.01856 (2024)

[15] Yan, Y., Jiang, J.-Y., Fu, M., Wang, D., Pelletier, A.R., Sigdel, D., Ng, D.C., Wang, W., Ping, P.: Mind-s is a deep-learning prediction model for elucidating protein post-translational modifications in human diseases. Cell reports methods **3**(3) (2023)

[16] Peng, F.Z., Wang, C., Chen, T., Schussheim, B., Vincoff, S., Chatterjee, P.: Ptm-mamba: a

ptm-aware protein language model with bidirectional gated mamba blocks. Nature Methods, 1–5 (2025)

[17] Sunar, E.A., Isik, Z., Pekey, M., Cinbis, R.G., Tastan, O.: Darkin: A zero-shot benchmark for phosphosite-dark kinase association using protein language models. bioRxiv, 2025–08 (2025)

[18] Wen, B., Wang, C., Li, K., Han, P., Holt, M.V., Savage, S.R., Lei, J.T., Dou, Y., Shi, Z., Li, Y., et al.: Deepmvp: deep learning models trained on high-quality data accurately predict ptm sites and variant-induced alterations. Nature Methods, 1–11 (2025)

[19] Hornbeck, P.V., Zhang, B., Murray, B., Kornhauser, J.M., Latham, V., Skrzypek, E.: Phosphositeplus, 2014: mutations, ptms and recalibrations. Nucleic acids research **43**(D1), 512–520 (2015)

[20] Castillo, J., Jodar, M., Oliva, R.: The contribution of human sperm proteins to the development and epigenome of the preimplantation embryo. Human reproduction update **24**(5), 535–555 (2018)

[21] Shrestha, P., Kandel, J., Tayara, H., Chong, K.T.: Post-translational modification prediction via prompt-based fine-tuning of a gpt-2 model. Nature Communications **15**(1), 6699 (2024)
