# Supplementary material for: Learning the PTM code through a coarse-to-fine mechanism-aware framework
Source: Nat Commun. 2026 May 15;17:6450. doi: 10.1038/s41467-026-73148-3 (PMC13377107; doi:10.1038/s41467-026-73148-3)
Supplement: Supplementary file 2 — Reporting Summary [file 41467_2026_73148_MOESM2_ESM.pdf]

nature portfolio

Corresponding author(s): Chang-Yu Hsieh, Chunbin Gu

Last updated by author(s): Apr 20, 2026

Reporting Summary

Nature Portfolio wishes to improve the reproducibility of the work that we publish. This form provides structure for consistency and transparency in reporting. For further information on Nature Portfolio policies, see our Editorial Policies and the Editorial Policy Checklist.

Statistics

For all statistical analyses, confirm that the following items are present in the figure legend, table legend, main text, or Methods section.

n/a	Confirmed	
[ ]	[x]	The exact sample size (*n*) for each experimental group/condition, given as a discrete number and unit of measurement
[ ]	[x]	A statement on whether measurements were taken from distinct samples or whether the same sample was measured repeatedly
[x]	[ ]	The statistical test(s) used AND whether they are one- or two-sided<br>*Only common tests should be described solely by name; describe more complex techniques in the Methods section.*
[x]	[ ]	A description of all covariates tested
[ ]	[x]	A description of any assumptions or corrections, such as tests of normality and adjustment for multiple comparisons
[ ]	[x]	A full description of the statistical parameters including central tendency (e.g. means) or other basic estimates (e.g. regression coefficient) AND variation (e.g. standard deviation) or associated estimates of uncertainty (e.g. confidence intervals)
[x]	[ ]	For null hypothesis testing, the test statistic (e.g. *F*, *t*, *r*) with confidence intervals, effect sizes, degrees of freedom and *P* value noted<br>*Give P values as exact values whenever suitable.*
[x]	[ ]	For Bayesian analysis, information on the choice of priors and Markov chain Monte Carlo settings
[x]	[ ]	For hierarchical and complex designs, identification of the appropriate level for tests and full reporting of outcomes
[x]	[ ]	Estimates of effect sizes (e.g. Cohen's *d*, Pearson's *r*), indicating how they were calculated

Our web collection on statistics for biologists contains articles on many of the points above.

Software and code

Policy information about availability of computer code

Data collection	no software was used.
Data analysis	The COMPASS-PTM model was developed and implemented using Python 3.12 and PyTorch 2.5.1. Data processing and analysis utilized NumPy 2.3.3 and Pandas 2.3.3. Sequence clustering and splitting were performed using MMseqs2 v16.747c6. Model evaluation and metrics calculation were conducted using scikit-learn 1.7.2. Visualization was performed using Matplotlib 3.10.3, Seaborn 0.13.2, and UMAP-learn 0.5.9. All custom code is available on GitHub (https://github.com/ZhangJJ26/COMPASS-PTM) under an MIT license.

For manuscripts utilizing custom algorithms or software that are central to the research but not yet described in published literature, software must be made available to editors and reviewers. We strongly encourage code deposition in a community repository (e.g. GitHub). See the Nature Portfolio guidelines for submitting code & software for further information.

Data

Policy information about availability of data

All manuscripts must include a data availability statement. This statement should provide the following information, where applicable:

- Accession codes, unique identifiers, or web links for publicly available datasets
- A description of any restrictions on data availability
- For clinical datasets or third party data, please ensure that the statement adheres to our policy

The COMPASS-PTM datasets generated and curated in this study have been deposited in Zenodo and are publicly available at https://doi.org/10.5281/zenodo.18389225. These files include the curated benchmark datasets, processed annotations, and train/validation/test splits used in this study. Source data are

provided with this paper.
The raw data used in this study are available from public databases. For the first stage (Multi-label Site Profiling), the raw post-translational modification data used in this study are available in the dbPTM database (https://biomics.lab.nycu.edu.tw/dbPTM/download.php), qPTM database (https://qptm.omicsbio.info/download.php), and PTMint database (https://ptmint.sjtu.edu.cn/Download). For the second stage (Enzyme–Substrate Pairing), the raw enzyme–substrate relationship data used in this study are available in the OmniPath database (https://omnipathdb.org/) and the SAGEPhos dataset repository (https://github.com/ZhangJJ26/SAGEPhos/releases). Full-length protein sequences used in this study are available in UniProt (https://www.uniprot.org/).

Research involving human participants, their data, or biological material

Policy information about studies with human participants or human data. See also policy information about sex, gender (identity/presentation), and sexual orientation and race, ethnicity and racism.

Reporting on sex and gender	This information has not been collected.
Reporting on race, ethnicity, or other socially relevant groupings	This information has not been collected.
Population characteristics	This information has not been collected.
Recruitment	This information has not been collected.
Ethics oversight	This information has not been collected.

Note that full information on the approval of the study protocol must also be provided in the manuscript.

Field-specific reporting

Please select the one below that is the best fit for your research. If you are not sure, read the appropriate sections before making your selection.

[x] Life sciences [ ] Behavioural & social sciences [ ] Ecological, evolutionary & environmental sciences

For a reference copy of the document with all sections, see nature.com/documents/nr-reporting-summary-flat.pdf

Life sciences study design

All studies must disclose on these points even when the disclosure is negative.

Sample size	No statistical methods were used to predetermine sample size. Sample sizes were determined based on the maximum availability of high-quality, curated data from public repositories, which are sufficient for training deep learning models with generalization capabilities.<br>In Stage 1, COMPASS-PTM was trained and evaluated on three constructed datasets: (1) dbPTM-ML (derived from dbPTM and UniProt), yielding 1,577,125 PTM sites; (2) qPTM-ML (derived from qPTM and UniProt), consisting of 662,072 PTM sites; and (3) PTMint-MC (derived from PTMint and UniProt), comprising 2,477 non-redundant PTM sites. In Stage 2, the model was trained and evaluated on the OmniPath and SAGEPhos datasets to facilitate enzyme-specific predictions. These datasets contain 39,201 enzyme-substrate interactions and 18,360 kinase-substrate pairs, respectively.
Data exclusions	PTM types with extremely limited data availability were aggregated into a generic 'Rare' category. Samples containing non-standard or invalid amino acid characters were excluded. We further validated the data by cross-referencing the PTM-centered peptides and their positions against full-length UniProt entries; any samples showing discrepancies or mapping failures were strictly excluded to prevent label noise.
Replication	We conducted multiple tests on individual methods, fully replicating the results reported in the paper, and the reported results represent the mean performance across these runs. Detailed instructions, source code, and datasets required to reproduce all findings are provided in the accompanying open-source repository.
Randomization	In Stage 1, sample allocation was based on sequence homology rather than randomization. We strictly partitioned the training, validation, and test sets using a 40% sequence similarity threshold via MMseqs2 to prevent data leakage. In Stage 2, three distinct splitting strategies were implemented: random splitting, enzyme-similarity-based splitting, and substrate-similarity-based splitting.
Blinding	The model was completely blinded to the test set throughout the training phase. The test set was strictly held out and was accessed only for the final performance evaluation, ensuring no data leakage occurred during model optimization.

Reporting for specific materials, systems and methods

We require information from authors about some types of materials, experimental systems and methods used in many studies. Here, indicate whether each material, system or method listed is relevant to your study. If you are not sure if a list item applies to your research, read the appropriate section before selecting a response.

Materials & experimental systems

n/a	Involved in the study
☒	☐ Antibodies
☒	☐ Eukaryotic cell lines
☒	☐ Palaeontology and archaeology
☒	☐ Animals and other organisms
☒	☐ Clinical data
☒	☐ Dual use research of concern
☒	☐ Plants

Methods

n/a	Involved in the study
☒	☐ ChIP-seq
☒	☐ Flow cytometry
☒	☐ MRI-based neuroimaging

Plants

Seed stocks: This information has not been collected.

Novel plant genotypes: This information has not been collected.

Authentication: This information has not been collected.
