# Supplementary material for: Learning the PTM code through a coarse-to-fine mechanism-aware framework
Source: Nat Commun. 2026 May 15;17:6450. doi: 10.1038/s41467-026-73148-3 (PMC13377107; doi:10.1038/s41467-026-73148-3)
Supplement: Supplementary file 3 — Transparent Peer Review file [file 41467_2026_73148_MOESM3_ESM.pdf]

nature portfolio

Peer Review File

Learning the PTM Code through a Coarse-to-Fine Mechanism-Aware Framework

Corresponding Author: Dr Chunbin Gu

This file contains all reviewer reports in order by version, followed by all author rebuttals in order by version.

Version 0:

Reviewer comments:

Reviewer #1

(Remarks to the Author)
The authors present COMPASS-PTM, a two-stage framework that combines multi-label PTM site prediction (MSPN) with an enzyme–substrate prediction module (ESPS). The system integrates ESM-2 embeddings, a chemical language model (SELF-BART), physicochemical features, and a crosstalk prior derived from PTMCode2. The overall idea looks good, and several of the reported results look good. However, I am a skeptical about the innovation and novelty of the approach. I share my major comments below.

Major Comment
1. While the proposed dual-modal architecture is a central contribution of the manuscript, the evaluation does not include the critical ablation experiments needed to validate the individual contributions of the PLM (ESM) and CLM components. The current ablation section examines the removal of certain modules (e.g., prompting, physicochemical features, LoRA), but it does not provide the core comparisons expected for a multimodal framework, namely: (i) a PLM-only vs. CLM-only model, (ii) the fusion of PLM+CLM without additional physicochemical features, and (iii) the full model vs. w/o CLM vs. w/o ESM. Without these baselines, it is difficult to assess whether the CLM genuinely contributes a meaningful signal or whether performance improvements arise almost entirely from ESM fine-tuning. Given that protein PLMs already encode substantial biochemical information, the additional value of CLM and physicochemical descriptors must be empirically demonstrated. These missing ablations are rquired, as they are necessary to substantiate the paper's claims about multimodal synergy.

2. The authors must explicitly check for overlap between the PTM associations in PTMCode2 and the test sets (dbPTM-ML/qPTM-ML). If the test set contains PTM pairs that were used to build the Rprior matrix, the model is not learning PTM grammar but merely retrieving memorized correlations. Although the authors acknowledge this risk in the Discussion section, simply acknowledging a flaw does not absolve the study of it, especially when it could fundamentally invalidate the results.

3. PTM crosstalk is often highly context-dependent (e.g., dependent on cell type, signaling state, adaptor proteins). Using a single global crosstalk matrix risks reinforcing co-occurrence patterns in contexts where they do not actually apply. The authors are encouraged to examine false positives, particularly on "orphan" sites where no known crosstalk exists, to evaluate whether the global prior induces hallucinated PTM interactions.

4. The authors report a macro-averaged F1-score of 0.423 for COMPASS-PTM on the dbPTM-ML dataset, compared to just 0.190 for the previous best method, SAGEPhos. Such a massive leap is impressive, but raises concerns about baseline fairness. SAGEPhos appears to be a method developed by the same corresponding author group. It is unclear if SAGEPhos was originally designed for multi-label prediction or if it was a binary/single-label predictor adapted for this task. If the baseline was not retrained with a multi-label objective, the comparison might be inherently unfair against the multi-label architecture of COMPASS-PTM. The authors should clarify how SAGEPhos and other existing predictors were adapted and trained to ensure a fair and meaningful comparison.

5. The manuscript repeatedly refers to COMPASS-PTM as “mechanism-aware,” but much of the model’s knowledge comes from co-occurrence statistics and curated enzyme–substrate pairs, which might represent correlation rather than causal biochemical mechanism. This issue is amplified by the model’s input design: Stage 1 uses relatively long sequence segments (≤50 aa), while Stage 2 restricts analysis to 15-mer windows. Many kinases (e.g., MAPKs, CDKs) rely on distal docking motifs located far outside a 15-residue window. As a result, the model cannot meaningfully capture these mechanistic determinants, and several of the interpretability claims may overreach what the architecture can actually represent. The authors should clarify the scope and limitations of their mechanistic claims.

6. It has to be noted that not all PTM’s are mediated by enzyme in that regard the enzyme prediction part has to be toned down to include only select group of PTM’s that are mediated by enzymes. The paper in fact only talks about Kinases aka phosphorylation.

Minor:
1. The adaptive segmentation approach should be formally defined for more clarity.

(Remarks on code availability)

Reviewer #2

(Remarks to the Author)
I co-reviewed this manuscript with one of the reviewers who provided the listed reports. This is part of the Nature Communications initiative to facilitate training in peer review and to provide appropriate recognition for Early Career Researchers who co-review manuscripts.

(Remarks on code availability)
The full datasets have not been provided in their GitHub repository. The README states that “the full dataset will be uploaded later,” which may become a bottleneck for reproducibility if the exact datasets are not made available.

Reviewer #3

(Remarks to the Author)
This paper introduces COMPASS-PTM, a two-stage, coarse-to-fine framework that jointly models residue-level multi-label PTM site prediction and enzyme–substrate assignment from sequence alone, arguing that these are two resolutions of the same underlying regulatory program rather than separable tasks. In Stage 1, the Multi-label Site Profiling Network (MSPN) fine-tunes ESM2 with LoRA and fuses it with a chemical modality derived from SELF-BART and explicit physicochemical descriptors via a gated residual fusion that treats the PLM as the primary signal. One of their key architectural contributions is a crosstalk-aware prompting mechanism that injects PTM co-occurrence priors (initialized from PTMCode2) directly into the attention logits through a learned PTM–PTM interaction matrix, enabling context-dependent, non-independent PTM predictions. They address the severe dual long-tail imbalance of PTM data using a hybrid macro–micro objective combining Dice loss, a probability-regularizing magnification term, and focal loss. In Stage 2, the Enzyme–Substrate Pairing System (ESPS), they freeze the PTM-aware substrate representation learned in Stage 1 and pairs it with enzyme embeddings (also derived from ESM2) using a dual-gated residual fusion module over 15-mer local substrate windows, predicting enzyme–site associations directly. Across their multiple benchmarks (dbPTM-ML, qPTM-ML, PTMint-MC, OmniPath, SAGEPhos, DARKIN), the framework reports large gains in macro-F1, MCC, AUC, and zero-shot kinase assignment, and the authors support interpretability claims via embedding visualizations, recovery of canonical kinase motifs, attribution analyses, and variant case studies that trace missense mutations through PTM loss/gain and inferred enzyme rewiring. Overall really good work!

I have some concerns that need to be addressed:

1. I think the crosstalk-aware prompting module is the most interesting part of the paper, but it is also where the risk of circularity is highest. The PTM–PTM interaction matrix is initialized from PTMCode2, and PTMCode2 itself aggregates curated PTM relationships that likely overlap with dbPTM, qPTM, and even downstream case studies. It would be great if the authors explicitly quantified this overlap and reran key experiments under stricter conditions: for example, (i) re-initializing the PTM–PTM matrix randomly, (ii) initializing it from a dataset disjoint in proteins from the training benchmarks, or (iii) training MSPN on PTM types whose co-occurrence statistics are deliberately withheld. Right now it is hard to tell how much of the performance and interpretability gain comes from learned generalization versus encoded prior knowledge.

2. The paper repeatedly frames the MSPN output as a PTM “program,” but almost all quantitative evaluation collapses back to residue-wise classification metrics such as macro-F1 and MCC? I think the program-level framing would be much stronger if the authors evaluated coherence at the protein level. For instance, it would be great to see metrics that assess whether predicted PTM sets respect known biochemical constraints, such as mutual exclusivity of lysine acetylation and ubiquitination at the same site, or whether predicted multi-PTM patterns better match experimentally observed combinations per protein compared to baselines. Without this, the notion of a learned regulatory program feels more conceptual than empirically demonstrated.

3. ESPS relies on a fixed 15-mer window around candidate sites, which is empirically justified via ablations, but this design choice has important biological implications. Many kinase and ligase recognition events depend on distal docking motifs, intrinsically disordered regions outside short windows, or domain-level context. It would be great if the authors evaluated sensitivity to window placement and length in a more targeted way, or tested whether incorporating longer-range substrate context improves cold-start enzyme generalization. At minimum, a clearer discussion of what classes of enzyme–substrate interactions this formulation can and cannot capture would help calibrate expectations.

4. I think the paper would benefit from a much more explicit comparison to PTM-Mamba (in Nature Methods, the authors may have incorrectly cited this as from a conference), which also proposes a PTM-aware sequence representation but from a fundamentally different angle. PTM-Mamba explicitly tokenizes modified residues and learns bidirectional sequence representations over wild-type and PTM-modified proteoforms using Mamba blocks, whereas COMPASS-PTM operates entirely on wild-type sequences and encodes PTM logic implicitly through multi-label supervision, co-occurrence prompting, and downstream enzyme pairing. Right now, this distinction is not clearly articulated, and it is easy for readers to conflate the two approaches. It would be great if the authors explicitly stated what information their representation can capture that PTM-Mamba cannot (for example, enzyme-specific rewiring from wild-type sequence alone), and conversely what they give up by not modeling explicit PTM tokens or modified proteoforms. A small experiment comparing MSPN embeddings to PTM-Mamba embeddings on a shared downstream task, or at least a conceptual diagram contrasting “implicit PTM programs” versus “explicit PTM tokenization,” would go a long way for me to see the novelty and scope of the contribution.

Minor Points

1. Fig. 2 radar plots are hard to read because absolute values are not labeled. I think adding numeric values directly to the bars or including a small table would improve interpretability.

2. UMAP and PCA figures (Fig. 4) reuse similar color palettes across panels, but the legend ordering changes. This makes it unnecessarily hard to track PTM types and kinase families across subfigures.

3. In Fig. 5 and Fig. 6, the probability values are shown with inconsistent precision (sometimes three decimals, sometimes four). Standardizing this would clean up the presentation.

4. The term “dual long-tail distribution” is introduced multiple times in the Introduction and Results. It would read better if it were defined once and then referenced consistently.

5. Several Supplementary figure references (e.g., S.2.x) appear before the figures are described conceptually in the main text.

6. Fig. 1b’s crosstalk-aware prompting module uses arrows and labels that are too small at print resolution. I think increasing font size or simplifying the diagram would help.

7. In the Methods, equations (3) and (4) introduce α twice with different meanings (scaling vs loss weighting). Renaming one would avoid confusion.

(Remarks on code availability)
The code is fine!

Version 1:

Reviewer comments:

Reviewer #1

(Remarks to the Author)
I am happy with the revision.

(Remarks on code availability)

Reviewer #2

(Remarks to the Author)
I co-reviewed this manuscript with one of the reviewers who provided the listed reports. This is part of the Nature Communications initiative to facilitate training in peer review and to provide appropriate recognition for Early Career Researchers who co-review manuscripts.

(Remarks on code availability)

Reviewer #3

(Remarks to the Author)
The authors have done a strong job of responding to my comments and I have no further requests. I recommend acceptance.

(Remarks on code availability)
The code is in good shape.

Open Access This Peer Review File is licensed under a Creative Commons Attribution 4.0 International License, which permits use, sharing, adaptation, distribution and reproduction in any medium or format, as long as you give appropriate credit to the original author(s) and the source, provide a link to the Creative Commons license, and indicate if changes were made.
In cases where reviewers are anonymous, credit should be given to 'Anonymous Referee' and the source.
The images or other third party material in this Peer Review File are included in the article's Creative Commons license, unless indicated otherwise in a credit line to the material. If material is not included in the article's Creative Commons license and your intended use is not permitted by statutory regulation or exceeds the permitted use, you will need to obtain permission directly from the copyright holder.
To view a copy of this license, visit https://creativecommons.org/licenses/by/4.0/

Responses to the Reviewers

We would like to express our sincere gratitude to the reviewers for their valuable and insightful comments. To address all of the reviewers' concerns, we provide point-to-point responses in this response letter. We also provide more experiments and discussions in our manuscript and highlight all the modifications using **red color**.

Reviewer #1

Comment 1.1: *1. While the proposed dual-modal architecture is a central contribution of the manuscript, the evaluation does not include the critical ablation experiments needed to validate the individual contributions of the PLM (ESM) and CLM components. The current ablation section examines the removal of certain modules (e.g., prompting, physicochemical features, LoRA), but it does not provide the core comparisons expected for a multimodal framework, namely: (i) a PLM-only vs. CLM-only model, (ii) the fusion of PLM+CLM without additional physicochemical features, and (iii) the full model vs. w/o CLM vs. w/o ESM. Without these baselines, it is difficult to assess whether the CLM genuinely contributes a meaningful signal or whether performance improvements arise almost entirely from ESM fine-tuning. Given that protein PLMs already encode substantial biochemical information, the additional value of CLM and physicochemical descriptors must be empirically demonstrated. These missing ablations are rquired, as they are necessary to substantiate the paper's claims about multimodal synergy.*

Response 1.1: We thank the reviewer for highlighting that, for a dual-modal framework, it is important to include core multimodal ablations that explicitly disentangle the respective contributions of the protein language model (PLM) and the chemical modality (CLM). We agree that such modality-level controls provide more direct evidence for the necessity and complementarity of the two modalities than the component-level ablations we originally reported (e.g., prompting, LoRA).

To directly address the reviewer's requested comparisons, we conducted additional architecture-level ablations on the dbPTM-ML dataset, using the same train/validation/test split and the identical evaluation protocol as the main experiments. Specifically, we evaluated: (i) **PLM-only** (ESM2 substrate representation with the same prediction head): macro-F1 = 30.69%; (ii) **PLM+CLM (w/o physchem)** (fusion of PLM with the CLM branch while removing explicit physicochemical descriptors): macro-F1 = 41.54%; (iii) **Full model w/o CLM** (removing the CLM branch while keeping the rest of the pipeline unchanged): macro-F1 = 35.09%; and (iv) **Full model**: macro-F1 = 42.32%. The performance are shown in Figure A**a**.

These ablations separate the effects of the PLM and the CLM branch, enabling a direct assessment of whether the CLM adds information beyond PLM-only modeling. **First**, the PLM-only baseline achieves a macro-F1 of 30.69%, indicating that sequence-only PLM representations by themselves are insufficient to reach strong multi-label PTM profiling performance in our setting. **Second**, adding the CLM branch to the PLM backbone even without explicit physicochemical descriptors improves macro-F1 from 30.69% to 41.54% (+10.85%), supporting that the chemical modality contributes substantial complementary signal beyond PLM-only modeling. **Third**, under an otherwise unchanged configuration, removing CLM from the full model reduces macro-F1 from 42.32% to 35.09% (–7.23%), showing that the gain is not driven by PLM fine-tuning alone, but reflects a material contribution from the CLM branch within the integrated architecture. **Fourth**, adding explicit physicochemical descriptors on top of PLM–CLM fusion further improves macro-F1 from 41.54% to 42.32% (+0.78%), indicating that these descriptors provide additional but smaller complementary refinement.

In addition, following the reviewer's suggestion, we implemented two extreme backbone-removal controls: **CLM-only** and **w/o ESM**. As expected, both perform very poorly (macro-F1 = 0.056 for CLM-only; macro-F1 = 0.159 for w/o ESM), indicating that the contextualized PLM backbone is essential to the full model. This is consistent with our design intent: in COMPASS-PTM, the CLM stream is introduced as an auxiliary residue-level representation that complements PLM-derived contextual features, rather than as an alternative backbone. Because these two settings lie far below the operating range of the within-family ablations and would substantially compress the visual differences among the more informative controlled comparisons, we report them in Supplementary Information text rather than in the main ablation panel.

Overall, these added ablations substantiate the manuscript's multimodal design claims at three levels: the PLM backbone is essential, the CLM branch contributes substantial complementary signal beyond PLM-only modeling, and explicit physicochemical descriptors provide an additional refinement on top of PLM–CLM fusion.

Changes in manuscript: We added these ablations in Supplementary Fig. 7 and discussed them in the revised Results 2.6 section and Supplementary Information S.2.7.1 section.

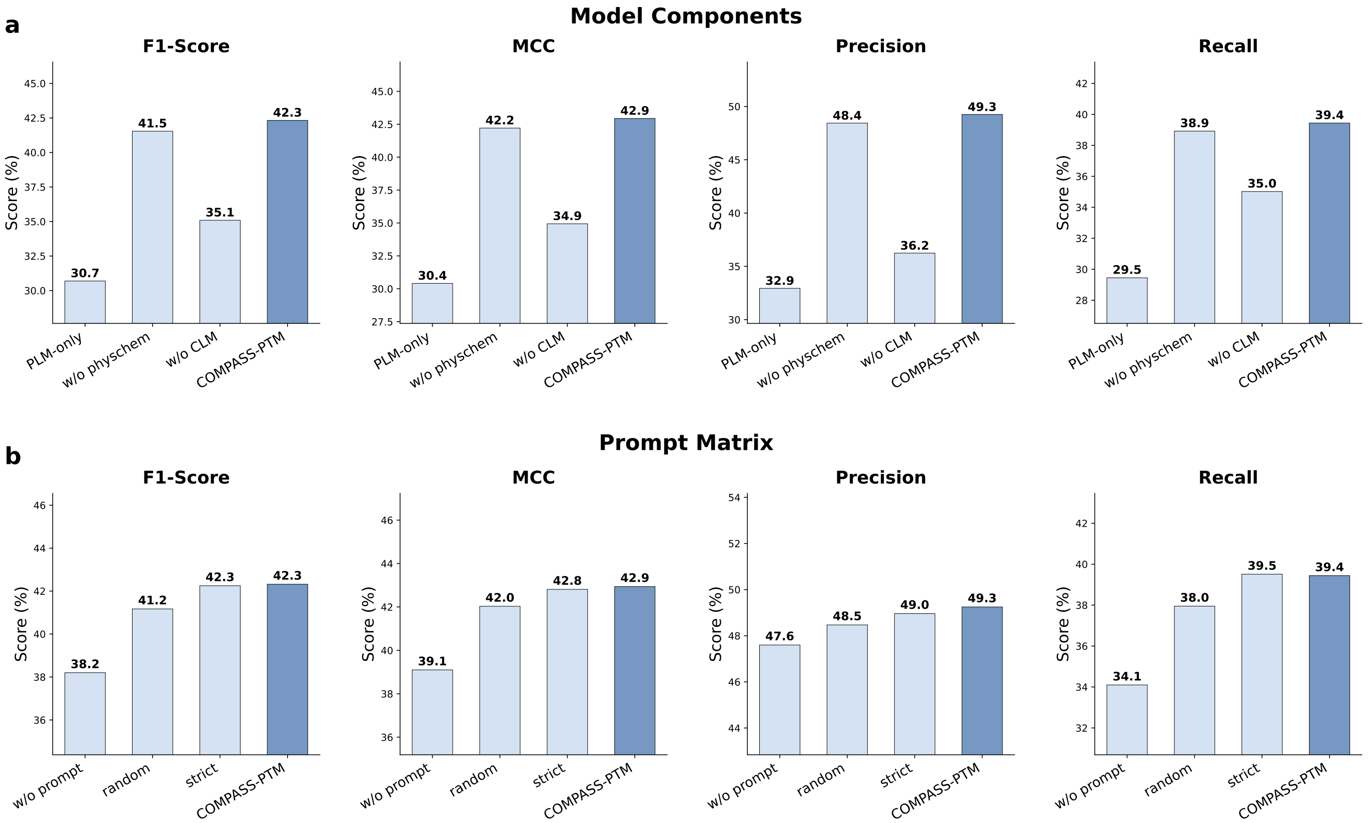

Figure A: **Ablation Studies of Model Components and Prompt Matrix Selection.** (**a**) Model-component ablations: PLM-only, w/o physchem, w/o CLM, and COMPASS-PTM. (**b**) Prompt-matrix ablations: w/o prompt, random, strict, and COMPASS-PTM. Bars show macro-F1, MCC, precision, and recall (in %; higher is better) under the same split and evaluation protocol; COMPASS-PTM is highlighted in dark blue.

Manuscript Results Section 2.6

And our hybrid loss function was confirmed to be highly effective, successfully balancing the precision-recall trade-off to outperform standard loss functions in the context of the severe dual long-tail data distribution. **Finally, our model component ablations further confirm that the performance gains are not attributable to any single ingredient: the PLM-anchored dual-modal design yields substantial improvements over a PLM-only baseline, and removing the CLM branch from the full pipeline leads to a pronounced degradation, supporting genuine multimodal synergy.** We further dissected the prompt matrix used by the crosstalk-aware prompting module by comparing the PTMCode2-initialized prior.

Manuscript Supplementary Information S.2.7.1

Model Component Ablations. To disentangle the contribution of each modality and the fusion pathway, we conducted architecture-level component ablations on dbPTM-ML under the same data split and evaluation protocol (Supplementary Fig. 7). We compare four settings: (i) **PLM-only**, which predicts PTM labels from ESM2-derived residue representations without any chemical branch; (ii) **w/o CLM**, which removes the CLM branch from the otherwise unchanged full pipeline; (iii) **w/o physchem (PLM+CLM, no physchem)**, which enables the CLM branch and PLM–CLM fusion while removing explicit physicochemical descriptors; and (iv) **COMPASS-PTM (Full)**, the complete model with PLM–CLM fusion and explicit physicochemical descriptors. We use the full model as the reference point and interpret each ablation by the degradation it induces relative to this reference. *First, removing the chemical pathway entirely* (PLM-only) causes a substantial performance drop relative to the full model, with macro-F1 falling from 42.32% to 30.69% (and similar declines across the other metrics). This indicates that sequence-only PLM representations, while informative, are insufficient for robust proteome-scale multi-label PTM profiling in our setting. *Second, removing the CLM branch within the otherwise unchanged full pipeline* (w/o CLM) also leads to a pronounced degradation, reducing

macro-F1 from 42.32% to 35.09%. This within-configuration drop directly supports that the CLM stream contributes materially to the integrated architecture rather than acting as a redundant auxiliary branch. *Third, removing only the explicit physicochemical descriptors* while retaining PLM–CLM fusion (w/o physchem) produces a smaller but still measurable decline, with macro-F1 decreasing from 42.32% to 41.54%. Thus, although the principal gain comes from multimodal PLM–CLM integration, the explicit physicochemical descriptors still provide complementary refinement and help the full model achieve the best overall performance. In addition, we implemented two extreme backbone-removal controls: **CLM-only** and strict **w/o ESM**. Both perform very poorly (macro-F1 $= 0.056$ for CLM-only; macro-F1 $= 0.159$ for w/o ESM), confirming that the contextualized PLM backbone is essential. Overall, these ablations support our PLM-anchored dual-modal design: the main performance gain arises from integrating the chemical branch with the PLM backbone, while explicit physicochemical descriptors provide additional complementary refinement. Together, the results support genuine multimodal complementarity rather than an improvement attributable to PLM fine-tuning alone.

Comment 1.2: *2. The authors must explicitly check for overlap between the PTM associations in PTMCode2 and the test sets (dbPTM-ML/qPTM-ML). If the test set contains PTM pairs that were used to build the Rprior matrix, the model is not learning PTM grammar but merely retrieving memorized correlations. Although the authors acknowledge this risk in the Discussion section, simply acknowledging a flaw does not absolve the study of it, especially when it could fundamentally invalidate the results.*

Response 1.2: We thank the reviewer for raising this important concern about potential circularity when external PTM resources (PTMCode2) are used to initialize our crosstalk prior. We agree that merely acknowledging this risk is not sufficient; the manuscript must state clearly what information is injected by the prior, whether it overlaps with benchmark annotations, and whether such overlap could invalidate the evaluation.

Clarifying our Discussion statement. In the Discussion, we previously noted that "*our use of PTM co-occurrence statistics and curated interaction networks as priors raises the possibility of circularity if the sources overlap with evaluation benchmarks; careful de-duplication and transparent reporting of data provenance will be essential for future extensions.*" Our intention was a forward-looking caution about future extensions in which richer priors, such as protein-/site-resolved interaction graphs or context-specific regulatory networks, might be incorporated. We agree, however, that this statement could be misread as implying that the current implementation directly leaks benchmark labels. We have therefore revised the text to explicitly distinguish: (i) broader provenance/circularity concerns relevant to future extensions, from (ii) the present paper's use of a global, PTM-type-level crosstalk statistic. The revised Discussion text is as follows:

> Manuscript Discussion
>
> Second, while our crosstalk-aware prompting is initialized from external PTM co-occurrence statistics and curated interaction resources, in this work the prior is used strictly as a global, type-level statistic and does not include protein-, site-, or sequence-resolved benchmark annotations. As a result, it does not constitute direct, instance-level test-set label leakage in the present evaluation. Nevertheless, as COMPASS-PTM is extended to incorporate richer priors—such as protein-/site-resolved interaction graphs or context-specific regulatory networks—careful de-duplication and transparent reporting of data provenance will remain essential to avoid circularity.

What R_{prior} actually contains (and what it does not contain). Although PTMCode2 entries are protein- and site-specific in their raw form (e.g., protein identifiers and residue indices), our preprocessing intentionally discards all such instance-level information. As implemented in `/models/preprocess/preprocess_matrix.py`, each PTM association is reduced to a coarse category defined only by (i) PTM type and (ii) modified residue class (single-letter amino-acid identity; e.g., phosphorylation@S or acetylation@K). Protein IDs, site indices, local sequence windows, and pair identifiers are removed, and benchmark annotations from dbPTM-ML/qPTM-ML are never used to build this prior.

We then aggregate these coarse categories across PTMCode2 to compute a global NPMI-based crosstalk statistic, which is used only to initialize a learnable interaction matrix $R \in \mathbb{R}^{C\times C}$ that is subsequently fine-tuned during training (Section 4.2.3 and Supplementary S.3.1.2). Therefore, R_{prior} contains no protein-, site-, or sequence-resolved information that could identify benchmark examples or encode test-set PTM labels at the instance level. In this sense, it cannot function as a lookup table for benchmark annotations; rather, it provides a dataset-agnostic bias toward broadly plausible PTM co-localization patterns at the PTM-type level.

Explicit overlap checks and stricter leakage control. We agree with the reviewer that potential overlap between PTMCode2-derived associations and the dbPTM-ML/qPTM-ML evaluation sets must be tested explicitly. In the revision, we therefore performed two levels of analysis. First, we explicitly screened for overlap at the **PTM-pair level** between PTMCode2 and the corresponding dbPTM-ML/qPTM-ML validation/test sets, and the numbers of overlapped PTMCode2 records are reported in Table A. To provide a stricter control against potential information leakage, we further applied **protein-level** filtering when constructing the revised prior. Concretely, for each benchmark split, we removed from PTMCode2 all records associated with proteins appearing in the corresponding validation/test sets, and then recomputed the NPMI matrix from the remaining records to obtain a train-only type-level prior (**strict** setting). We confirmed that all PTM-pair overlaps detected by the explicit pair-level screening were fully covered by this protein-level exclusion. Therefore, the strict setting is more conservative than removing only the matched PTM pairs, because it eliminates every detected pair overlap together with any additional PTMCode2 associations linked to those same evaluation proteins.

Importantly, this stricter filtering had almost no effect on the resulting type-level prior itself. Comparing the original and strict NPMI prompt matrices on their upper-triangular entries, we obtained Pearson $r = 0.9993$, Spearman $\rho = 0.9929$, MAE $= 4.35 \times 10^{-4}$, and RMSE $= 2.50 \times 10^{-3}$. Thus, after excluding PTMCode2 records associated with benchmark-overlapping proteins, the reconstructed crosstalk matrix remains nearly identical to the original one. This indicates that the global PTM-type dependency structure captured by the prior is highly stable and is not materially driven by benchmark-overlapping proteins.

Second, we performed three complementary prompt robustness controls on dbPTM-ML: **(a) w/o prompt**, where the attention-logit bias is removed entirely; **(b) random**, where R is randomly initialized (but remains learnable), thereby destroying PTM-

TABLE A: **Overlap filtering statistics for the PTMCode2 crosstalk source.** We first explicitly screened PTMCode2 for benchmark-specific PTM-pair overlap with the validation or test sets, and then applied a stricter protein-level de-duplication by removing all PTMCode2 records whose protein IDs overlap with the corresponding validation or test proteins. The remaining records were used to recompute the train-only, type-level NPMI prior ("strict" setting).

Benchmark	Split filtered	Original records	Removed (PTM-pair overlap)	Removed (protein overlap)
dbPTM-ML	Test	2,741,240	2,595	27,312
dbPTM-ML	Valid	2,741,240	3,149	22,013
qPTM-ML	Test	2,741,240	1,922	13,191
qPTM-ML	Valid	2,741,240	1,380	11,550

type co-occurrence semantics while preserving parameter count and architectural form; and **(c) strict**, where R_{prior} is recomputed after removing from PTMCode2 all records associated with proteins appearing in the validation/test sets.

As shown in Fig. A**b** (Prompt Matrix), the **strict** prior performs comparably to the default COMPASS-PTM initialization. Both **strict** and default COMPASS-PTM consistently outperform the learnable **random** initialization, and all prompted variants substantially exceed the **w/o prompt** setting. Together, these analyses directly test the reviewer's concern: even after explicitly removing benchmark-overlapping proteins from PTMCode2 and rebuilding a train-only type-level prior, both the prior matrix itself and the downstream performance remain essentially unchanged. This is inconsistent with the interpretation that the model is merely retrieving memorized benchmark correlations from PTMCode2. Instead, the results support our intended interpretation that the prompt contributes a soft, learnable PTM-type inductive bias rather than benchmark-specific annotation retrieval.

Changes in manuscript: We added the explicit overlap/provenance analyses in Supplementary Table 4 and Supplementary Fig. 7, discussed them in revised Results Section 2.6 and Supplementary Information Section S.2.7.1.

Manuscript Results Section 2.6

Finally, our model component ablations further confirm that the performance gains are not attributable to any single ingredient: the PLM-anchored dual-modal design yields substantial improvements over a PLM-only baseline, and removing the CLM branch from the full pipeline leads to a pronounced degradation, supporting genuine multimodal synergy. **We further dissected the prompt matrix used by the crosstalk-aware prompting module by comparing the PTMCode2-initialized prior with a strict de-duplicated prior recomputed after removing proteins overlapping with validation/test splits. The strict prior remained nearly identical to the original prompt matrix and performed indistinguishably from the default COMPASS-PTM initialization, while both outperformed a learnable random initialization. These results indicate that the prompt acts as a soft, learnable inductive bias rather than a benchmark-specific lookup mechanism (see Supplementary Note S.2.7.1 and Supplementary Fig. 7 for full details).**

Manuscript Supplementary Information S.2.7.1

Prompt Matrix Ablations and Robustness to Provenance Overlap. The crosstalk-aware prompting module injects a PTM-type dependency bias into attention through a learnable interaction matrix R, which is initialized from an external crosstalk resource (PTMCode2) and then fine-tuned end-to-end. A key question is whether the observed gain could be explained by circularity if the external resource overlaps with benchmark proteins. We note that R_{prior} is computed from aggregated PTM-type co-occurrence statistics and does not contain protein IDs, site indices, sequence windows, or other instance-resolved benchmark information. It therefore provides only a type-level prior and cannot specify the ground-truth label assignment for any individual benchmark example. To provide empirical support, we first rebuilt the PTMCode2-based prior after excluding all records associated with proteins appearing in the validation/test splits. The resulting strict prior remained almost unchanged relative to the original matrix (upper-triangular comparison: Pearson $r = 0.9993$, Spearman $\rho = 0.9929$, MAE $= 4.35 \times 10^{-4}$, RMSE $= 2.50 \times 10^{-3}$), indicating that the global PTM-type crosstalk structure is highly stable under protein-overlap filtering. We then compared four settings: (i) Original prior (**COMPASS-PTM**); (ii) **Strict** de-duplicated prior (train-only provenance control). To explicitly eliminate any potential provenance overlap at the protein level, we removed from the PTMCode2 crosstalk source all records associated with proteins appearing in the validation/test splits and recomputed the type-level NPMI matrix from the remaining records (Supplementary Table 4). Importantly, this control retains the same type-level formulation but ensures that the prior is derived from proteins disjoint from evaluation proteins. (iii) **Random** initialization (learnable but uninformative). We also replace the PTMCode2 initialization with a randomly initialized matrix of the same shape, which is still learnable during training. This controls for the possibility that the gain arises merely from adding extra parameters or a generic attention bias, rather than from meaningful crosstalk structure. (iv) No prompt (**w/o prompt**). Finally, we remove the prompting term entirely, providing a lower-bound reference where the model must learn multi-label dependence

purely from sequence-conditioned supervision. As shown in Supplementary Fig. 7**b**, the **strict** de-duplicated prior achieves performance that is essentially indistinguishable from the original COMPASS-PTM initialization across macro-F1/MCC/precision/recall, indicating that the observed gains are not driven by benchmark overlap. In addition, both **strict** and **COMPASS-PTM** consistently outperform the learnable but semantically uninformative **random** initialization, and all prompted variants outperform the **w/o prompt** setting. Collectively, these results support our intended interpretation: even after explicitly removing from PTMCode2 all crosstalk records associated with proteins in the validation/test splits and recomputing a **strict** prior, performance remains essentially unchanged, ruling out a shortcut "retrieval" explanation based on shared test-set PTM pairs. Rather, the improvement arises from injecting meaningful PTM-type dependency structure as a soft, learnable inductive bias, beyond what can be obtained from an unstructured (random) initialization.

Comment 1.3: *3. PTM crosstalk is often highly context-dependent (e.g., dependent on cell type, signaling state, adaptor proteins). Using a single global crosstalk matrix risks reinforcing co-occurrence patterns in contexts where they do not actually apply. The authors are encouraged to examine false positives, particularly on "orphan" sites where no known crosstalk exists, to evaluate whether the global prior induces hallucinated PTM interactions.*

Response 1.3: We thank the reviewer for highlighting this important point. We agree that PTM crosstalk is often context-dependent, and that a single global crosstalk matrix could, in principle, introduce false-positive PTM interactions in contexts where they do not truly apply.

To directly test this risk, we performed a targeted false-positive analysis on the dbPTM-ML test set under the same split and evaluation protocol as in the main experiments. Specifically, we compared the same model trained **with** the global crosstalk bias and **without** it (**w/ bias** vs. **w/o bias**). Our goal was to answer a simple question: `does the global prior cause the model to add unsupported extra PTM labels at a residue?`

How we define spurious predictions. For each residue i, let Y_i be the ground-truth PTM label set and $\hat{Y}_i$ the predicted label set after thresholding (threshold $= 0.5$, same as in the main evaluation). We define the unsupported extra labels at that residue as $\hat{Y}_i \setminus Y_i$. We then report two simple metrics: (i) **`spurious_any_rate`**: the fraction of residues that receive at least one unsupported extra PTM label; (ii) **`avg_spurious_per_fp_site`**: the average number of unsupported extra PTM labels among residues that have any such error.

We report these metrics in two views. The first is an **unconditional** view over all evaluated residues. The second is a **conditional** view restricted to residues where the model already predicts at least one correct PTM label ($|\hat{Y}_i \cap Y_i| > 0$). The conditional view is especially relevant here, because it directly tests whether the global prior makes the model "pile on" extra unsupported PTM types after making a correct call.

Results. As shown in Table B, adding the global crosstalk bias causes only a very small absolute change in the fraction of residues with any unsupported extra label. On all residues, the **`spurious_any_rate`** changes from 0.02028 (**w/o bias**) to 0.02123 (**w/ bias**) in the unconditional setting, and from 0.09544 to 0.09571 in the conditional setting. These changes are very small. Importantly, the severity of these errors does not increase. The **`avg_spurious_per_fp_site`** is slightly lower with the bias in every setting: for example, 1.0364 vs. 1.0419 in the unconditional all-site analysis, and 1.0677 vs. 1.0736 in the conditional analysis. In other words, when spurious extra labels occur, the global prior does not make them worse.

A stricter stress test: single-label residues. We agree that the reviewer's concern is most relevant for "orphan" cases. In the current benchmark, however, it is difficult to define residue-level sites with "no known crosstalk" in a fully reliable way, because external resources PTMCode2 do not provide an exhaustive, benchmark-aligned site-level annotation of crosstalk absence, so absence from them cannot be treated as a reliable negative label. Instead, we performed a stricter and benchmark-defined stress test on residues with only one curated PTM label ($|Y_i| = 1$). In this subset, any additional predicted PTM type is, by definition, an unsupported extra label under the benchmark protocol. Even in this stricter setting, the increase in **`spurious_any_rate`** remains small: 0.06066 to 0.06334 in the unconditional view, and 0.09231 to 0.09301 in the conditional view. Again, the **`avg_spurious_per_fp_site`** slightly decreases with the bias (1.1114 vs. 1.1238 unconditionally; 1.0681 vs. 1.0755 conditionally).

TABLE B: **Spurious (hallucinated) PTM analysis with vs. without the global crosstalk bias** (threshold = 0.5). We report (i) the fraction of residues with any unsupported extra PTM type (`spurious_any_rate`), and (ii) the average number of unsupported extra PTM types per such residue (`avg_spurious_per_fp_site`). "Conditional" denotes evaluation restricted to residues where at least one true PTM label is correctly predicted ($|\hat{Y}_i \cap Y_i| > 0$).

Setting	Subset	spurious_rate (FPR) ↓	avg_spurious_per_fp_site ↓
Unconditional	All sites	0.02123 (w/ bias) vs. 0.02028 (w/o)	1.0364 (w/ bias) vs. 1.0419 (w/o)
Unconditional	Single-label ($|Y|$=1)	0.06334 (w/ bias) vs. 0.06066 (w/o)	1.1114 (w/ bias) vs. 1.1238 (w/o)
Conditional ($|\hat{Y} \cap Y| > 0$)	All sites	0.09571 (w/ bias) vs. 0.09544 (w/o)	1.0677 (w/ bias) vs. 1.0736 (w/o)
Conditional ($|\hat{Y} \cap Y| > 0$)	Single-label ($|Y|$=1)	0.09301 (w/ bias) vs. 0.09231 (w/o)	1.0681 (w/ bias) vs. 1.0755 (w/o)

Why this is consistent with the model design. The crosstalk term is implemented as a soft attention-logit bias rather than a hard constraint, and the PTM–PTM interaction matrix is learnable and fine-tuned end-to-end. This allows the model to attenuate prior correlations when the local substrate evidence does not support them, rather than being forced to follow a fixed global co-occurrence table.

Conclusion. Overall, these analyses do not support the concern that the global crosstalk prior systematically hallucinates PTM interactions. Across both the full test set and the stricter single-label subset, adding the global prior leads to only very small absolute changes in the probability of producing any unsupported extra label, and it does not increase the number of extra labels when such errors occur. We therefore interpret the benefit of the crosstalk module as helping the model reinforce PTM combinations that are already compatible with the local substrate signal, rather than indiscriminately inflating PTM label sets.

Comment 1.4: *4. The authors report a macro-averaged F1-score of 0.423 for COMPASS-PTM on the dbPTM-ML dataset, compared to just 0.190 for the previous best method, SAGEPhos. Such a massive leap is impressive, but raises concerns about baseline fairness. SAGEPhos appears to be a method developed by the same corresponding author group. It is unclear if SAGEPhos was originally designed for multi-label prediction or if it was a binary/single-label predictor adapted for this task. If the baseline was not retrained with a multi-label objective, the comparison might be inherently unfair against the multi-label architecture of COMPASS-PTM. The authors should clarify how SAGEPhos and other existing predictors were adapted and trained to ensure a fair and meaningful comparison.*

Response 1.4: We thank the reviewer for the careful assessment of baseline fairness. We agree that, given the large performance gap on dbPTM-ML (macro-F1 0.423 vs. 0.190), it is essential to clarify (i) whether prior baselines—especially SAGEPhos—were truly evaluated under a multi-label objective consistent with our task, and (ii) whether they were retrained under the same protocol as COMPASS-PTM. We apologize that this was not described with sufficient precision in the original manuscript, and we have added baseline descriptions accordingly.

Uniform training/evaluation protocol. To ensure a rigorous comparison, we retrained all baselines from scratch using **the same train/validation/test splits** as COMPASS-PTM on dbPTM-ML (and analogously on qPTM-ML), the same PTM label set, and the same evaluation code for macro-F1/MCC. All methods are evaluated as **multi-label predictors** under an identical test-time protocol. For baselines whose original formulations are binary/single-label, we minimally modify their output heads to produce one score per PTM type and train them under a standard multi-label loss.

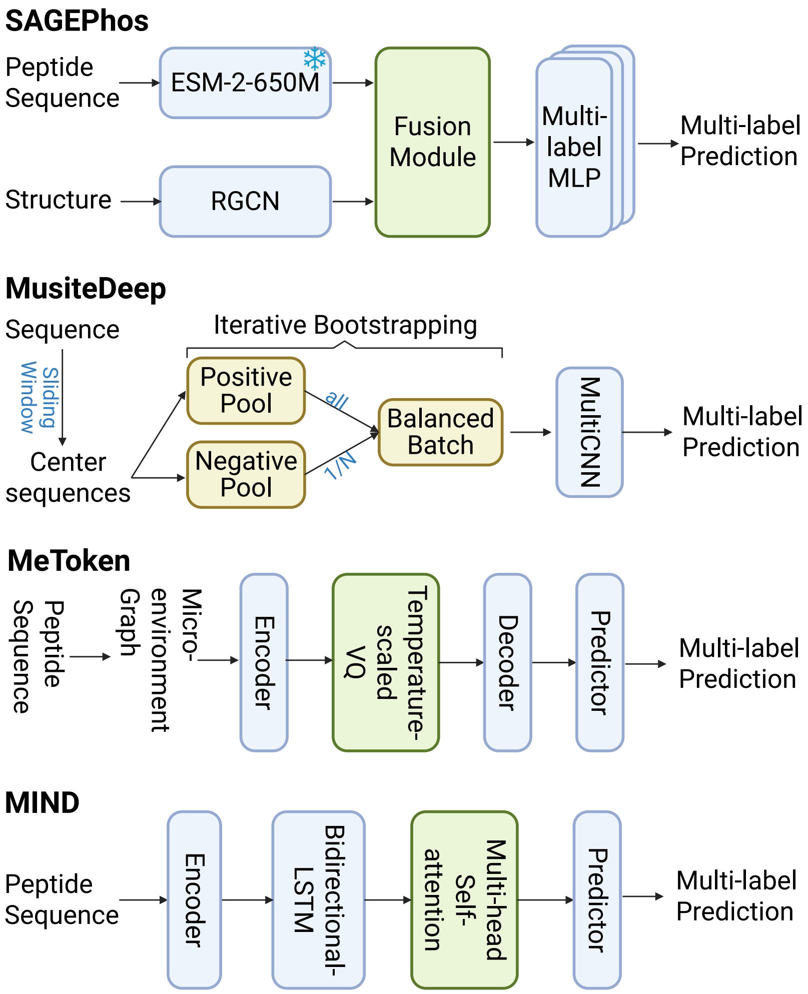

Figure B: **Baseline model architectures.** Schematic overview of four representative baselines considered in this study: SAGEPhos, MusiteDeep, MeToken, and MIND-S.

Baseline-specific adaptations. As illustrated in the revised baseline architecture figure B, we made the minimal changes required to make each method compatible with residue-level multi-label PTM prediction:

- **SAGEPhos.** SAGEPhos was originally introduced in our prior work under an enzyme–substrate association formulation. For this manuscript, we adapted it to residue-level multi-label PTM site prediction by (i) using the same substrate inputs as COMPASS-PTM, and (ii) replacing the original binary head with a multi-label MLP head that outputs one probability

per PTM type, as shown in the adapted SAGEPhos diagram. The adapted model was retrained on the COMPASS-PTM training split and evaluated on the same test split. In response to the reviewer's question about why the gap can be large despite the same author group, we note that the adapted SAGEPhos is a minimal retrofit of a model originally designed for enzyme–substrate association, whereas COMPASS-PTM is explicitly optimized for residue-level multi-label PTM profiling. In the adapted SAGEPhos, the main representational capacity largely comes from the frozen ESM2 substrate encoder, and the modification is primarily confined to replacing the binary head with a multi-label classifier. By contrast, COMPASS-PTM introduces several components that directly target the multi-label residue-level objective, including LoRA-based adaptation of the PLM to PTM supervision, the incorporation of a complementary chemical modality (CLM), and the crosstalk-aware prompting module. Our ablations show that these additions account for substantial portions of the observed gains, explaining why a minimally adapted baseline can lag behind a purpose-built multi-modal, crosstalk-aware framework, even when evaluated under the same split and protocol.

- **MusiteDeep.** MusiteDeep is historically formulated as independent binary predictors. We adapted it to our multi-label setting by expanding the output layer to C sigmoid units (one per PTM type) and training with multi-label BCE, while preserving its core CNN/bootstrapping design (as depicted). The model was retrained on the same training split and evaluated on the same test split.
- **MeToken.** MeToken was originally designed for multi-class prediction. For our multi-label PTM profiling benchmark, we keep the original encoder–decoder model unchanged and adapt its prediction procedure to produce a set of PTM labels per residue under the same evaluation protocol as COMPASS-PTM (as depicted). The adapted model was retrained on the same training split.
- **MIND-S.** MIND-S is inherently multi-label (BiLSTM + multi-head self-attention). We followed the original architecture and retrained it on our training split to ensure a fair, dataset-matched baseline.

Conclusion. With these adaptations and retraining, SAGEPhos and all other baselines are evaluated as proper multi-label predictors under the same data splits and evaluation protocol as COMPASS-PTM. Therefore, the reported improvement (macro-F1 0.423 on dbPTM-ML) reflects the advantage of our proposed modeling choices (dual-stage learning, multimodal fusion, and PTM-dependency modeling), rather than differences stemming from inconsistent training or evaluation protocols across methods.

Changes in manuscript: We added a brief baseline description in the main text. And we added a dedicated Baseline adaptation and retraining protocol subsection (Supplementary Information S.1.4) and included the revised baseline architecture diagram (Supplementary Fig. 3).

Manuscript Results Section 2.2.1

We compared MSPN against representative state-of-the-art PTM predictors (SAGEPhos, MusiteDeep, MeToken, and MIND-S). For a fair comparison under the multi-label setting, each baseline was adapted where necessary to match the benchmark label space and then retrained using the same train/validation/test splits and evaluation protocol as our model. Full implementation details and baseline adaptations are provided in Supplementary Information **S.1.4**.

Manuscript Supplementary Information S.1.4

To ensure a fair and reproducible comparison under residue-level multi-label PTM site prediction, we evaluated MSPN against four representative baselines: SAGEPhos, MusiteDeep, MeToken, and MIND-S. For all baselines, we used the **same** train/validation/test splits as COMPASS-PTM on each benchmark (dbPTM-ML and qPTM-ML), and we report results using an identical evaluation pipeline (macro-averaged metrics; Supplementary Information S.1.2). When a baseline's original formulation did not natively match the multi-label setting (multiple PTM types may co-occur at the same residue), we applied the minimal architectural modifications necessary to align the output space and supervision with the benchmark label set while preserving the core encoder design.

a) SAGEPhos (adapted to multi-label prediction).: SAGEPhos was originally for an enzyme–substrate association formulation. For the substrate-level multi-label PTM task in this manuscript, we adapted SAGEPhos to predict PTM types at each residue by replacing the original prediction head with a multi-label MLP that outputs one score per PTM type. The upstream representation learning components (sequence encoder based on ESM2 and the fusion module; with an optional structural branch when applicable) were kept consistent with the baseline architecture, and the adapted model was retrained from scratch on our training split.

b) MusiteDeep (binary-to-multi-label head adaptation).: MusiteDeep is historically formulated as independent binary predictors. To align it with the multi-label benchmark setting, we expanded its output layer to produce C outputs corresponding to the benchmark PTM label set. The remainder of the architecture, including its CNN backbone and iterative bootstrapping strategy, was preserved. The adapted MusiteDeep was retrained from scratch using our training split and selected using the same validation protocol.

c) MeToken (codebook assignment adapted for PTM co-occurrence).: MeToken employs a vector-quantized (VQ) codebook and an encoder–decoder objective, where the original quantization step typically enforces a near-hard assignment of each input to a single codeword. In multi-label PTM prediction, however, multiple PTM types can co-occur at the same residue, which benefits from representations that can simultaneously express multiple discrete factors. To accommodate our benchmark setting, we apply a minimal adaptation by softening the codebook assignment via temperature scaling, allowing an input representation to be expressed as a mixture over multiple codewords rather than collapsing to a single discrete token. We retrain the model from scratch on the same training split with model selection on the same validation split.

d) MIND-S (native multi-label baseline).: MIND-S is natively designed for multi-label prediction (BiLSTM encoder with multi-head self-attention and a multi-label predictor). We kept the original architecture intact and retrained it from scratch on our training split to obtain a dataset-matched baseline under the same evaluation protocol.

e) Training and evaluation consistency.: Across all baselines, the final reported numbers were computed on the held-out test set using the same metric implementation (macro-F1, macro-MCC, etc.). This ensures that the observed improvements reflect differences in modeling assumptions and representational capacity rather than mismatched label spaces or evaluation procedures.

Comment 1.5: *5. The manuscript repeatedly refers to COMPASS-PTM as "mechanism-aware," but much of the model's knowledge comes from co-occurrence statistics and curated enzyme–substrate pairs, which might represent correlation rather than causal biochemical mechanism. This issue is amplified by the model's input design: Stage 1 uses relatively long sequence segments (less than 50 aa), while Stage 2 restricts analysis to 15-mer windows. Many kinases (e.g., MAPKs, CDKs) rely on distal docking motifs located far outside a 15-residue window. As a result, the model cannot meaningfully capture these mechanistic determinants, and several of the interpretability claims may overreach what the architecture can actually represent. The authors should clarify the scope and limitations of their mechanistic claims.*

Response 1.5: We thank the reviewer for pointing out an important distinction between causal biochemical mechanism and statistical correlates.

Mechanism-aware scope and limitations. In this work, we use "mechanism-aware" to denote enzyme-grounded and specificity-aware PTM regulation, rather than a claim of causal pathway-level biochemical mechanism. Concretely, COMPASS-PTM couples residue-level PTM site profiling (Stage 1, MSPN) with enzyme–substrate assignment (Stage 2, ESPS), extending the prediction target from "where a PTM may occur" to "who most plausibly catalyzes it". This enzyme-resolved coupling grounds site-level predictions in catalytic regulation and distinguishes COMPASS-PTM from purely site-centric models that output PTM propensities without regulatory attribution.

We also agree that some enzymes rely on distal docking motifs and domain-/scaffold-level context beyond a short local window. Our current ESPS instantiation represents the substrate by a 15-mer peptide centered on the candidate site; thus it is well-suited for capturing proximal motif grammar but does not explicitly encode distal docking interactions. We will clarify this limitation and temper interpretability statements accordingly in the revised manuscript.

Terminology revision.

To avoid over-interpretation, we will revise the text wherever "mechanism-aware" could be misconstrued as pathway-level causal claims. In the revised manuscript, we will retain "mechanism-aware" in a limited, framework-level sense, and explicitly define it at first use as enzyme-resolved, specificity-aware modeling. Elsewhere, we will preferentially adopt more precise phrasing such as "enzyme-resolved assignment" and "mechanistically interpretable hypotheses".

Manuscript Introduction

Guided by this view, we introduce **COMPASS-PTM** (Coarse-to-fine Oriented Multi-label PTM Site Prediction And enzyme-Substrate aSsignment), a two-stage, mechanism-aware **(enzyme-resolved specificity-aware modeling)** framework that learns a single, unified PTM-aware representation bridging both levels of inference,

Manuscript Discussion

Third, the current model primarily encodes linear sequence information. It does not explicitly account for higher-order structural features, cellular context, or temporal dynamics of signaling, all of which are known to modulate PTM accessibility and enzyme specificity. **In addition, ESPS represents substrates using a site-centered 15-mer, which is well-suited for capturing proximal recognition motifs but cannot explicitly model distal docking interactions or long-range domain/scaffold effects.**

Empirical motivation of the 15-mer window. The 15-mer choice was empirically motivated: an ablation over 15/21/51-aa substrate contexts shows 15-aa to be optimal under the OmniPath enzyme–substrate benchmark setting (Supplementary Fig. 6h), indicating that within this benchmark the dominant learnable signal concentrates in the immediate flanking residues, whereas longer windows likely introduce additional contextual noise/ambiguity. In parallel, Stage 1 MSPN uses adaptive segmentation (peptides $\leq$ 50 aa containing at least one PTM site) for site profiling, preserving broader local context for coarse PTM "program" inference.

New analysis to directly test long-range substrate context. To directly address the reviewer's concern about distal determinants, we implemented an ESPS variant that augments the 15-mer local substrate representation with full-length substrate context. Specifically, we compute a global substrate sequence embedding $\mathbf{H}^{\text{global}}$ over the full-length substrate sequence, and fuse it with the local 15-mer hidden states $\mathbf{H}^{\text{local}}$ via a single lightweight cross-attention layer with residual connection:

$$\mathbf{H}^{\text{local}} \leftarrow \text{LayerNorm}\Big(\mathbf{H}^{\text{local}} + \text{Dropout}(\text{Attn}(\mathbf{Q} = \mathbf{H}^{\text{local}}, \mathbf{K} = \mathbf{H}^{\text{global}}, \mathbf{V} = \mathbf{H}^{\text{global}}))\Big).$$

We evaluate this variant under the same training protocol and the same OmniPath enzyme–substrate split as the default ESPS.

Overall, adding full-length substrate context yields a small but consistent increase in ranking-oriented metrics (AUROC/AUPRC) and a marginal increase in accuracy, while F1 remains essentially unchanged. This pattern suggests that, for this benchmark, the dominant learnable signal for enzyme–site compatibility is already concentrated in the immediate flanking residues captured

Setting	ACC	F1	AUROC	AUPRC
15aa ESPS (default)	0.7909	**0.7981**	0.8633	0.8436
15aa + full-length substrate cross-attn (new)	**0.7918**	0.7951	**0.8678**	**0.8496**

TABLE C: **Full-length substrate context in ESPS on the OmniPath enzyme–substrate benchmark.** We compare the default site-centered 15aa substrate formulation to a variant that injects full-length substrate context via cross-attention. Best values are in bold.

by the 15aa window, such that the local ESPS formulation can match the global-context variant to within a narrow margin. We therefore interpret the global-context results as being consistent with the view that, under the current benchmark and supervision, much of the predictive signal for enzyme–site compatibility is already captured by the site-centered 15aa window. Distal docking and domain-/scaffold-level determinants remain biologically important for particular enzyme families; however, because the curated labels do not indicate which distal segments mediate recognition, a straightforward full-length fusion may not translate that information into a clear or systematic improvement in this setting. Practically, the full-length fusion also introduces additional model and inference complexity. For this reason, we retain the 15aa ESPS as the default instantiation, while clarifying in the revised manuscript that capturing distal docking and domain/scaffold effects likely requires more targeted long-range modeling (e.g., sparse retrieval of candidate docking segments, domain/disorder-aware cues, or structure-informed constraints) and/or finer-grained supervision.

Comment 1.6: *6. It has to be noted that not all PTM's are mediated by enzyme in that regard the enzyme prediction part has to be toned down to include only select group of PTM's that are mediated by enzymes. The paper in fact only talks about Kinases aka phosphorylation.*

Response 1.6: We thank the reviewer for this important clarification and agree that the scope of the enzyme-assignment component should be stated more precisely.

Clarify scope of Stage 2 (ESPS). Not all PTMs are strictly enzyme-mediated, and even for PTMs that are commonly enzyme-catalyzed, non-enzymatic chemistry can occur in specific contexts. We have therefore revised the manuscript to avoid over-generalizing the enzyme-assignment claims. Specifically, COMPASS-PTM comprises two stages with different intended scopes: **Stage 1 (MSPN)** performs residue-level multi-label PTM site profiling and is applicable to any PTM type represented in supervision, whereas **Stage 2 (ESPS)** is formulated and evaluated only for enzyme-mediated PTM regulations where a cognate regulator is defined through curated enzyme–substrate annotations. Throughout the revised manuscript, we describe ESPS as an enzyme–substrate pairing/assignment module in this setting, and we avoid implying applicability to PTMs without well-defined catalytic regulators or to non-enzymatic modification processes.

Coverage beyond kinase. We also acknowledge that phosphorylation and kinases appear prominently in our examples and discussion. This emphasis is primarily driven by data availability: kinase–substrate annotations are currently the most abundant and reliable across public resources, and kinase recognition motifs are among the best-characterized, making them a natural choice for illustrative case studies. Importantly, however, ESPS is not restricted to kinases in formulation. In our OmniPath-based evaluation, ESPS is applied to multiple enzyme-mediated modification classes with curated regulator–substrate annotations, including phosphorylation/dephosphorylation and other enzyme-mediated PTMs such as acetylation and methylation. For transparency, we summarize the PTM-category coverage statistics and regulator types in Supplementary Fig. 1**i**, and we have revised the Abstract/Introduction/Results to clearly state that Stage 2 is evaluated only where curated regulator–substrate supervision is available.

Manuscript Introduction

Rather than acting as an independent classifier, ESPS asks which enzymes best explain the PTM patterns encoded by MSPN for a given **enzyme-mediated substrate.** In this way, Stage 2 refines the coarse PTM program produced by Stage 1 into a mechanistic map that ties specific **enzyme-mediated** modification sites and patterns to the **annotated** enzymes that write or erase them, without discarding the information learned at the site level.

Manuscript Results Section 2.2.3

The second stage of our framework, Enzyme–Substrate Pairing System (ESPS), demonstrates exceptional predictive power on both our curated OmniPath dataset and the established SAGEPhos benchmark. We evaluate ESPS only on enzyme-mediated PTMs with curated enzyme–substrate annotations: OmniPath spans multiple enzyme-mediated modification classes beyond kinases, whereas SAGEPhos is restricted to kinase–substrate (phosphorylation) relationships.

Manuscript Supplementary Information S.1.1.1

OmniPath is a meta-database that integrates molecular prior knowledge from more than 100 publicly available resources to enable systems-level analysis of cellular regulation. Its core innovation lies in standardizing fragmented biological data into five interoperable modules: signaling networks, annotated protein complexes, multi-attribute protein annotations, intercellular communication roles, and critically—a comprehensive repository of curated enzyme–substrate relationships underlying diverse enzyme-mediated PTMs. Importantly, this PTM module is not restricted to kinases/phosphorylation: beyond kinase–substrate interactions, it also includes other enzymatic modification classes such as acetylation (acetyltransferases), methylation (methyltransferases), ubiquitination (E1/E2/E3 cascade), as shown in Supplementary Fig. 1**i**. The PTM module, curated from 11 specialized resources, defines 39,201 enzyme–substrate interactions involving 1,821 enzymes and 16,467 substrate-specific modification sites across humans, mice, and rats.

Comment 1.7: *Minor: 1. The adaptive segmentation approach should be formally defined for more clarity.*

Response 1.7: We thank the reviewer for pointing out that our "adaptive segmentation" strategy should be defined more formally. We agree that this clarity is important for reproducibility, and we have revised the manuscript to explicitly define the procedure in the Methods and to point readers to the Supplementary Information where the full details are provided.

Stage 1 (MSPN): adaptive greedy segmentation + sequence-space split. In Stage 1, "adaptive segmentation" refers to a greedy segmentation algorithm that converts each protein into a minimal set of local peptides for training, under two constraints: (i) each peptide has length ($\leq$ 50) aa, and (ii) each peptide must contain at least one annotated PTM site. Concretely, we traverse PTM sites along the sequence and dynamically choose cleavage boundaries so that each extracted peptide covers one or more nearby PTM sites while avoiding non-informative fragments that contain no positives. This differs from a fixed-step sliding window because boundaries are chosen adaptively to satisfy the "($\leq$ 50) aa and ($\geq$ 1) PTM site" constraints and to eliminate redundant windows. We now state this explicitly in the revised Methods. To reduce evolutionary leakage during evaluation, we define the train/val/test split by clustering proteins using MMseqs2 at 40% sequence identity and then splitting clusters into 80%/10%/10% subsets. This is now explicitly referenced when introducing the segmentation procedure. These descriptions already appear in Supplementary Section S.1.1.2.

Stage 2 (ESPS): warm/cold-start splits. For ESPS, peptides are extracted as fixed-length windows centered on the candidate modification site, and "adaptive evaluation" refers to our three formally defined splitting settings: warm-start, substrate cold-start (test substrates absent from training), and enzyme cold-start (test enzymes absent from training). Negative samples are constructed by selecting unmodified residues of the same amino-acid type within the same substrate peptide, maintaining a 1:1 positive/negative ratio. We have added an explicit pointer to Supplementary Section S.1.1.3, where these settings are defined.

Changes in manuscript: We revised the Methods to formally define the Stage 1 adaptive segmentation procedure, i.e., an adaptive greedy construction of PTM-site–anchored peptides ($\leq$ 50 aa) from full-length proteins, and we added explicit pointers to Supplementary Section S.1.1.2 for complete dataset-processing details. In addition, we clarified the Stage 2 evaluation protocol by describing the warm-start and two cold-start split settings (substrate cold-start and enzyme cold-start), with full split specifications referenced in Supplementary Section S.1.1.3.

Manuscript Results Section 2.2.1

To rigorously evaluate the performance and generalization capabilities of the first stage MSPN, we curated a suite of three distinct benchmark datasets from established proteomics resources. Our evaluation suite comprises two multi-label benchmarks, dbPTM-ML and qPTM-ML, which we developed from the dbPTM and qPTM datasets, respectively, to assess the model on the complex biological reality of sites harboring multiple, co-occurring PTM types. To specifically test the model's generalization to a different task formulation, we also included the PTMint-MC dataset, a multi-class benchmark where each site is annotated with a single PTM type, developed from PTMint dataset. **Across these benchmarks, proteins are processed into PTM-site–anchored segments (≤ 50 aa) and split at the protein level via MMseqs2 clustering (40% identity) to minimize redundancy and evolutionary leakage, detailed specifications for each dataset are provided in the Supplementary Information S.1.1.2.**

Manuscript Results Section 2.2.4

The second stage of our framework, Enzyme–Substrate Pairing System (ESPS), demonstrates exceptional predictive power on both our curated OmniPath dataset and the established SAGEPhos benchmark. We evaluate ESPS only on enzyme-mediated PTMs with curated enzyme–substrate annotations: OmniPath spans multiple enzyme-mediated modification classes beyond kinases, whereas SAGEPhos is restricted to kinase–substrate (phosphorylation) relationships. **To assess generalization under realistic deployment scenarios, we evaluate ESPS under three split settings: a warm-start setting where enzymes and substrates may both appear in training and test sets, and two stringent cold-start settings that hold out novel entities, including (i) a substrate cold-start split where all test substrates are excluded from training, and (ii) an enzyme cold-start split where all test enzymes are absent from training. Dataset construction, split protocols, and additional implementation details are provided in Supplementary Information S.1.1.3.**

Manuscript Methods Section 4.2.1

For Stage 1 training, we convert each full-length protein sequence $S = \{s_i\}_{i=1}^{L}$ (length L) with an annotated PTM-site set $\mathcal{P} = \{p_k\}_{k=1}^{M}$ (M sites) into a set of substrate peptides $\mathcal{W} = \{(l_t, r_t)\}_{t=1}^{T}$ (T peptides) via an adaptive greedy segmentation procedure. Each peptide is a contiguous subsequence $S[l_t : r_t]$ subject to two constraints: (i) $r_t - l_t + 1 \leq 50$, and (ii) it contains at least one annotated PTM site (i.e., $\mathcal{P} \cap [l_t, r_t] \neq \emptyset$).

Algorithmically, we process PTM sites in ascending order and iteratively form the next peptide to cover the leftmost uncovered site, choosing boundaries (l_t, r_t) to include as many subsequent nearby PTM sites as possible while respecting the 50-aa limit. This yields a compact set of informative peptides (no all-negative fragments) and avoids the redundant overlap induced by fixed-step sliding windows. Full details are provided in Supplementary Section S.1.1.2.

Reviewer #2

Comment 2.1: *The full datasets have not been provided in their GitHub repository. The README states that "the full dataset will be uploaded later," which may become a bottleneck for reproducibility if the exact datasets are not made available.*

Response 2.1: We thank the reviewer for highlighting the importance of full data availability for reproducibility. We agree that the original wording in our GitHub README ("the full dataset will be uploaded later") could create uncertainty and may hinder immediate reuse.

To clarify, our GitHub repository currently provides (i) the trained checkpoints and (ii) a representative dataset sample to illustrate the exact data format and preprocessing pipeline. The **full training/evaluation datasets** used in this study have now been uploaded to **Zenodo**, the direct download link is "https://zenodo.org/records/18389225". This ensures that the precise datasets corresponding to all reported experiments are publicly accessible and can be reproduced end-to-end.

Reviewer #3

Comment 3.1: *1. I think the crosstalk-aware prompting module is the most interesting part of the paper, but it is also where the risk of circularity is highest. The PTM–PTM interaction matrix is initialized from PTMCode2, and PTMCode2 itself aggregates curated PTM relationships that likely overlap with dbPTM, qPTM, and even downstream case studies. It would be great if the authors explicitly quantified this overlap and reran key experiments under stricter conditions: for example, (i) re-initializing the PTM–PTM matrix randomly, (ii) initializing it from a dataset disjoint in proteins from the training benchmarks, or (iii) training MSPN on PTM types whose co-occurrence statistics are deliberately withheld. Right now it is hard to tell how much of the performance and interpretability gain comes from learned generalization versus encoded prior knowledge.*

Response 3.1: We thank the reviewer for highlighting the potential circularity risk of the crosstalk-aware prompting module and for suggesting stricter provenance controls. We agree that, because the PTM–PTM interaction matrix is initialized from PTMCode2, it is important to disentangle gains arising from learned generalization from those attributable to injected prior knowledge.

Clarification on what the prior can (and cannot) encode. In our formulation, the PTM–PTM matrix prior is not constructed at the protein- or site-instance level. Instead, R_{prior} is computed from aggregated PTM-type co-occurrence statistics (NPMI) over coarse PTM categories (PTM type paired with residue class), and it deliberately excludes protein identifiers, residue indices, peptide windows, and local sequence context. Consequently, even if PTMCode2 contains records derived from proteins that also appear in dbPTM/qPTM, the prior itself does not contain the information required to look up benchmark annotations at the instance level; it can only encode broad, type-level co-occurrence tendencies, and it is further fine-tuned end-to-end during training.

Benchmark-aware overlap controls. Nevertheless, we agree with the reviewer that overlap in source provenance could still bias the prior toward benchmark-associated PTM relationships, and this should be tested explicitly. In the revision, we therefore implemented the most direct benchmark-aware controls among the reviewer's suggestions (Fig. C): (i) **Random re-initialization.** We replace the PTM–PTM matrix initialization with a random matrix of the same shape (still learnable), thereby breaking PTM-type co-occurrence semantics while controlling for parameter count and optimization. (ii) **Protein-disjoint (strict) initialization.** We explicitly quantified protein-ID overlap between PTMCode2 and the benchmark validation/test proteins (Table D), removed all PTMCode2 crosstalk records associated with those proteins, and recomputed a **strict** train-only type-level NPMI prior from the remaining records. (iii) **No-prompt control.** We also removed the prompting term entirely (**w/o prompt**) as a lower-bound reference.

TABLE D: **Protein-level overlap filtering statistics for the PTMCode2 crosstalk source.** We performed protein-ID based de-duplication by removing PTMCode2 crosstalk records whose protein IDs overlap with the validation or test sets of our benchmarks. The remaining records were used to recompute the train-only, type-level NPMI prior ("strict" setting).

Benchmark	Split filtered	Filtering strategy	Original records	Removed (overlap)
dbPTM-ML	Test	Protein-level filtering	2,741,240	27,312
dbPTM-ML	Valid	Protein-level filtering	2,741,240	22,013
qPTM-ML	Test	Protein-level filtering	2,741,240	13,191
qPTM-ML	Valid	Protein-level filtering	2,741,240	11,550

Importantly, this stricter filtering had almost no effect on the resulting type-level prior itself. Comparing the original and strict NPMI prompt matrices on their upper-triangular entries, we obtained Pearson $r = 0.9993$, Spearman $\rho = 0.9929$, MAE $= 4.35 \times 10^{-4}$, and RMSE $= 2.50 \times 10^{-3}$. Thus, after excluding PTMCode2 records associated with benchmark-overlapping proteins, the reconstructed crosstalk matrix remains nearly identical to the original one, indicating that the global PTM-type dependency structure is highly stable under this provenance control.

As shown in Fig. C, the **strict** prior achieves essentially the same performance as the original COMPASS-PTM initialization, while both **strict** and COMPASS-PTM consistently outperform the learnable **random** initialization and substantially outperform **w/o prompt**. Taken together, these controls make two points clear. First, the performance gain is not materially driven by proteins overlapping between PTMCode2 and the benchmark validation/test splits, because removing those proteins leaves both the prior matrix and downstream performance essentially unchanged. Second, the advantage of the original/**strict** priors over a learnable but semantically uninformative **random** initialization indicates that the benefit does not arise merely from adding extra parameters or an arbitrary attention bias. Rather, the results support the interpretation that the prompt contributes meaningful PTM-type dependency structure as a soft, learnable inductive bias, rather than serving as a benchmark-specific retrieval mechanism.

Changes in manuscript: We added the explicit overlap/provenance analyses in Supplementary Table 4 and Supplementary Fig. 7, and discussed them in the revised Results Section 2.6 and Supplementary Information Section S.2.7.1.

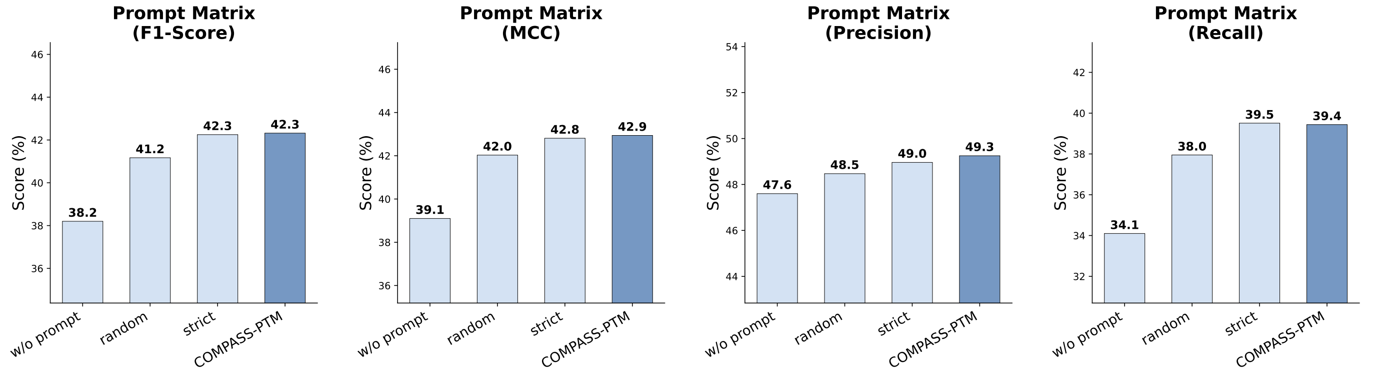

Figure C: **Ablation Studies of Prompt Matrix Selection.** Bars show macro-F1, MCC, precision, and recall (in %; higher is better) under the same split and evaluation protocol; COMPASS-PTM is highlighted in dark blue.

Manuscript Results Section 2.6

Finally, our model component ablations further confirm that the performance gains are not attributable to any single ingredient: the PLM-anchored dual-modal design yields substantial improvements over a PLM-only baseline, and removing the CLM branch from the full pipeline leads to a pronounced degradation, supporting genuine multimodal synergy. **We further dissected the prompt matrix used by the crosstalk-aware prompting module by comparing the PTMCode2-initialized prior with a strict de-duplicated prior recomputed after removing proteins overlapping with validation/test splits. The strict prior remained nearly identical to the original prompt matrix and performed indistinguishably from the default COMPASS-PTM initialization, while both outperformed a learnable random initialization. These results indicate that the prompt acts as a soft, learnable inductive bias rather than a benchmark-specific lookup mechanism (see Supplementary Note S.2.7.1 and Supplementary Fig. 7 for full details).**

Manuscript Supplementary Information S.2.7.1

Prompt Matrix Ablations and Robustness to Provenance Overlap. The crosstalk-aware prompting module injects a PTM-type dependency bias into attention through a learnable interaction matrix R, which is initialized from an external crosstalk resource (PTMCode2) and then fine-tuned end-to-end. A key question is whether the observed gain could be explained by circularity if the external resource overlaps with benchmark proteins. We note that R_{prior} is computed from aggregated PTM-type co-occurrence statistics and does not contain protein IDs, site indices, sequence windows, or other instance-resolved benchmark information. It therefore provides only a type-level prior and cannot specify the ground-truth label assignment for any individual benchmark example. To provide empirical support, we first rebuilt the PTMCode2-based prior after excluding all records associated with proteins appearing in the validation/test splits. The resulting strict prior remained almost unchanged relative to the original matrix (upper-triangular comparison: Pearson $r = 0.9993$, Spearman $\rho = 0.9929$, MAE $= 4.35 \times 10^{-4}$, RMSE $= 2.50 \times 10^{-3}$), indicating that the global PTM-type crosstalk structure is highly stable under protein-overlap filtering. We then compared four settings: (i) Original prior (**COMPASS-PTM**); (ii) **Strict** de-duplicated prior (train-only provenance control). To explicitly eliminate any potential provenance overlap at the protein level, we removed from the PTMCode2 crosstalk source all records associated with proteins appearing in the validation/test splits and recomputed the type-level NPMI matrix from the remaining records (Supplementary Table 4). Importantly, this control retains the same type-level formulation but ensures that the prior is derived from proteins disjoint from evaluation proteins. (iii) **Random** initialization (learnable but uninformative). We also replace the PTMCode2 initialization with a randomly initialized matrix of the same shape, which is still learnable during training. This controls for the possibility that the gain arises merely from adding extra parameters or a generic attention bias, rather than from meaningful crosstalk structure. (iv) No prompt (**w/o prompt**). Finally, we remove the prompting term entirely, providing a lower-bound reference where the model must learn multi-label dependence purely from sequence-conditioned supervision. As shown in Supplementary Fig. 7**b**, the **strict** de-duplicated prior achieves performance that is essentially indistinguishable from the original COMPASS-PTM initialization across macro-F1/MCC/precision/recall, indicating that the observed gains are not driven by benchmark overlap. In addition, both **strict** and **COMPASS-PTM** consistently outperform the learnable but semantically uninformative **random** initialization, and all prompted variants outperform the **w/o prompt** setting. Collectively, these results support our intended interpretation:

even after explicitly removing from PTMCode2 all crosstalk records associated with proteins in the validation/test splits and recomputing a **strict** prior, performance remains essentially unchanged, ruling out a shortcut "retrieval" explanation based on shared test-set PTM pairs. Rather, the improvement arises from injecting meaningful PTM-type dependency structure as a soft, learnable inductive bias, beyond what can be obtained from an unstructured (random) initialization.

Comment 3.2: *2. The paper repeatedly frames the MSPN output as a PTM "program," but almost all quantitative evaluation collapses back to residue-wise classification metrics such as macro-F1 and MCC? I think the program-level framing would be much stronger if the authors evaluated coherence at the protein level. For instance, it would be great to see metrics that assess whether predicted PTM sets respect known biochemical constraints, such as mutual exclusivity of lysine acetylation and ubiquitination at the same site, or whether predicted multi-PTM patterns better match experimentally observed combinations per protein compared to baselines. Without this, the notion of a learned regulatory program feels more conceptual than empirically demonstrated.*

Response 3.2: We thank the reviewer for highlighting an important gap between our conceptual framing of a protein-level "PTM program" and our original emphasis on residue-wise metrics such as macro-F1 and MCC. We agree that, if this framing is to be convincing, it should be supported by analyses that go beyond site-wise correctness. To directly strengthen the empirical support for the "PTM program" interpretation, we added three complementary analyses that quantify protein/program-level coherence beyond residue-wise macro-F1/MCC.

(1) Protein-level PTM program agreement.

To instantiate the "PTM program" framing at the protein level, we added a protein-wise evaluation in which each protein is treated as a structured output object, represented as a set of residue–PTM events. For each protein p, we compute event-set F1 between the predicted and ground-truth PTM event sets, and then average across proteins. This analysis evaluates whether the predicted PTM composition over an entire protein matches the observed protein-level modification pattern, rather than only measuring residue-wise correctness in aggregate.

Using this protocol, COMPASS-PTM achieves a mean protein-level F1 of 0.6430, indicating that its improvement is not limited to local residue classification but extends to the protein-level PTM event composition.

(2) Spurious multi-label burden: coherence of the predicted PTM set at a residue.

An important aspect of program-level coherence is whether, once a residue is predicted as modified, the associated PTM set remains clean and well restrained, rather than being expanded with additional unsupported PTM types. In our analysis, we found that a common failure mode of strong baselines is precisely this kind of multi-label over-calling: after recovering one true PTM label at a site, the model tends to add extra PTM types beyond the curated label set. Such errors can artificially inflate apparent combinatorial PTM patterns and thereby weaken the interpretation of a coherent protein-level PTM program. We therefore explicitly quantified the spurious multi-label burden.

For each residue i, let Y_i denote the ground-truth PTM label set and $\widehat{Y_i}$ the predicted set. A residue is counted as spurious if $\widehat{Y_i} \setminus Y_i \neq \emptyset$. We report two quantities: (i) `spurious_rate`, the fraction of evaluated residues with at least one extra predicted PTM type beyond the ground truth; and (ii) `avg_spurious_per_fp_site`, the average number of extra PTM types among spurious residues.

We evaluate two complementary settings. In the **unconditional** setting, all evaluated residues are included in the denominator, reflecting realistic end-to-end scanning of a protein sequence. In the **conditional** setting, we restrict to residues where the model already predicts at least one correct PTM label, i.e., $\widehat{Y_i} \cap Y_i \neq \emptyset$, and then ask whether the model still adds unnecessary extra PTM types on these correctly localized modified sites. The conditional setting is especially relevant to the reviewer's concern because it focuses on the coherence of the predicted PTM set once a site has already been identified as modified.

We further report results on both **all sites** and the **single-label subset** ($|Y_i| = 1$). The single-label subset is particularly informative because the annotation itself indicates a single curated PTM type, so any additional predicted PTM type is unambiguously an unsupported extra call.

TABLE E: **Spurious multi-label analysis (SAGEPhos vs. COMPASS-PTM).** `spurious_rate`: fraction of residues with at least one extra predicted PTM type beyond the ground truth. `avg_spurious_per_fp_site`: average number of extra PTM types among spurious residues. Unconditional uses all evaluated residues as the denominator; Conditional restricts to residues where at least one true PTM label is correctly recovered.

Setting	Subset	Model	spurious_rate ↓	avg_spurious_per_fp_site ↓
Unconditional	all_sites	SAGEPhos	0.7621	1.7555
		COMPASS-PTM	**0.0212**	**1.0364**
	single-label ($\lvert Y\rvert = 1$)	SAGEPhos	0.7156	1.4445
		COMPASS-PTM	**0.0633**	**1.1114**
Conditional	all_sites	SAGEPhos	0.6541	1.2993
		COMPASS-PTM	**0.0957**	**1.0677**
	single-label ($\lvert Y\rvert = 1$)	SAGEPhos	0.6513	1.2971
		COMPASS-PTM	**0.0930**	**1.0681**

As shown in Table E, COMPASS-PTM substantially reduces this failure mode relative to the strongest baseline SAGEPhos. Under the unconditional all-sites setting, the spurious rate decreases from 0.7621 to 0.0212, indicating that COMPASS-PTM does not generate widespread unsupported co-labeling during proteome-scale scanning. The same pattern holds in the single-

label subset, where the spurious rate decreases from 0.7156 to 0.0633, substantially reducing the risk of fabricating combinatorial PTM patterns on residues annotated with a single PTM type.

Crucially, the advantage persists under the conditional setting, which more directly reflects program-level coherence after a site has already been correctly recognized as modified. On all sites, the conditional spurious rate decreases from 0.6541 to 0.0957. This means that, once a true PTM label has been recovered, COMPASS-PTM is far less likely than SAGEPhos to add additional unsupported PTM types. Moreover, when spurious predictions do occur, their multiplicity is also lower in COMPASS-PTM (1.04–1.11 extra labels on average) than in SAGEPhos (1.30–1.76), indicating that COMPASS-PTM produces cleaner and more interpretable PTM-type compositions at modified residues.

(3) Residue-type biochemical feasibility.

Beyond set-level coherence, we also evaluated biochemical plausibility at the residue level by examining whether residue-restricted PTM types are spuriously assigned to incompatible amino-acid types. This provides a direct test of whether the model's predictions remain biochemically disciplined rather than merely accurate in an aggregate classification sense.

In our analysis, we found that a common qualitative failure mode of the strongest baseline is to assign PTM types to residue types on which they are biochemically infeasible. By contrast, COMPASS-PTM rarely produces such feasibility-violating predictions, consistent with its substantially lower spurious multi-label burden and its cleaner residue-type constrained behavior. This further supports that COMPASS-PTM learns a more biologically plausible PTM output structure.

Takeaway.

In summary, we agree with the reviewer that residue-wise macro-F1/MCC alone are insufficient to fully support the "PTM program" framing. We therefore strengthened the manuscript with three complementary analyses: protein-level PTM-program agreement, spurious multi-label burden, and residue-type biochemical feasibility. Together, these results show that COMPASS-PTM improves not only residue-wise predictive accuracy, but also the coherence, restraint, and biological plausibility of the inferred PTM event composition at the protein level.

Changes in manuscript: We revised the Results and Supplementary Information to add protein-level PTM-program agreement analysis, spurious multi-label burden analysis, and residue-type biochemical feasibility analysis, thereby providing stronger empirical support for the "PTM program" interpretation beyond residue-wise macro-F1/MCC.

Manuscript Results Section 2.2.2

While residue-wise macro-F1 and MCC quantify per-residue correctness, a "PTM program" interpretation additionally requires that the PTM set assigned at each site remain coherent and restrained. A common multi-label failure mode is over-calling: after a site is localized as modified, the model assigns extra PTM types beyond the curated label set, which can inflate apparent combinatorial PTM patterns and blur protein-level regulatory structure.

To quantify this aspect of program-level coherence, we evaluate spurious multi-label burden in both overall and conditional settings, further stratified by all sites versus the single-label subset. We also report the average number of extra labels per spurious residue to capture error severity (details in Supplementary Information S.2.1 and Supplementary Table 1).

Across the multi-label benchmarks, COMPASS-PTM substantially reduces both the frequency and severity of spurious over-calling relative to the strongest baseline, SAGEPhos. In the conditional setting, where at least one true PTM label is already recovered at a residue, the spurious rate decreases from 0.654 to 0.096, indicating that COMPASS-PTM is far less likely to assign additional unsupported PTM types once a site is localized. Moreover, when spurious errors do occur, COMPASS-PTM typically adds only one extra label on average (1.04–1.11), whereas SAGEPhos more often produces multiple extra labels (1.30–1.76). The same pattern holds on the single-label subset, supporting that COMPASS-PTM produces cleaner and more coherent PTM label sets rather than relying on over-expanded multi-label predictions.

Consistent with these set-level coherence gains, COMPASS-PTM achieves a mean protein-level event-F1 of 0.6430 on the test set, indicating that the predicted residue–PTM event collection aligns with the ground-truth program at the level of whole proteins.

Manuscript Supplementary Information S.2.1

Residue-wise metrics (macro-F1/MCC) quantify per-residue correctness but do not directly assess whether the predicted PTM label sets are coherent. In multi-label PTM site prediction, a frequent failure mode is over-calling: after localizing a modified residue, the model assigns additional PTM types beyond the curated label set, which can artificially inflate apparent PTM co-occurrence and weaken protein-level "PTM program" interpretation. To quantify this behavior, we introduce complementary program-level coherence metrics and report them in Supplementary Table 1.

Evaluation Metrics

Notation and thresholding. For residue i, let Y_i denote the ground-truth PTM label set and $\widehat{Y}_i$ the predicted set after

thresholding. For a fair comparison, we apply the same thresholding strategy used in the main benchmark to all methods (COMPASS-PTM and baselines), without method-specific tuning beyond the validation split (i.e., the operating point used to compute macro-F1/MCC is reused here).

Spurious multi-label burden. We define a residue as spurious if it contains any predicted PTM labels not present in the ground truth:

$$\mathrm{Spurious}(i) = \mathbb{I}\big[\widehat{Y}_i \setminus Y_i \neq \emptyset\big]. \tag{1}$$

We report three related quantities: (i) an unconditional spurious rate over all evaluated residues, (ii) a conditional spurious rate restricted to residues where at least one true PTM label is correctly recovered, and (iii) the average number of extra labels per spurious residue:

$$\mathrm{SpuriousRate}_{\mathrm{uncond}} = \frac{1}{N}\sum_{i=1}^{N}\mathrm{Spurious}(i), \tag{2}$$

$$\mathrm{SpuriousRate}_{\mathrm{cond}} = \frac{1}{|\mathcal{S}|}\sum_{i\in\mathcal{S}}\mathrm{Spurious}(i), \quad \mathcal{S} = \{i \mid \widehat{Y}_i \cap Y_i \neq \emptyset\}, \tag{3}$$

$$\mathrm{AvgExtraLabels} = \mathbb{E}\Big[|\widehat{Y}_i \setminus Y_i| \;\Big|\; \widehat{Y}_i \setminus Y_i \neq \emptyset\Big]. \tag{4}$$

We additionally report results on the single-label subset $\{i \mid |Y_i| = 1\}$ to isolate unambiguous coherence cases, where extra PTM types are particularly undesirable.

Protein-level event-F1. To connect residue-level sets to protein-level "programs", for each protein p we form an event set $E_p = \{(i,t) \mid t \in Y_i\}$ and a predicted event set $\widehat{E}_p = \{(i,t) \mid t \in \widehat{Y}_i\}$, compute an event-level F1 per protein, and macro-average across proteins.

Analysis and interpretation.

Supplementary Table 1 shows that COMPASS-PTM produces substantially more coherent multi-label outputs than SAGEPhos, beyond improvements in residue-wise macro-F1/MCC.

First, COMPASS-PTM dramatically reduces the frequency of spurious over-calling. Under the unconditional evaluation, which reflects end-to-end proteome-scale scanning, the spurious rate decreases from 0.7621 (SAGEPhos) to 0.0212 (COMPASS-PTM) on all sites, and from 0.7156 to 0.0633 on the single-label subset. This indicates that COMPASS-PTM does not generate widespread unsupported co-labeling when scanning full protein sequences. The advantage remains clear under the conditional evaluation, which isolates set-level coherence once at least one true PTM label is recovered at a residue: the spurious rate further decreases from 0.6541 to 0.0957 on all sites, and from 0.6513 to 0.0930 on the single-label subset. Together, these results show that COMPASS-PTM is not only better at localizing modified residues, but also markedly more disciplined in deciding which PTM types to assign once a site is recognized as modified.

Second, COMPASS-PTM also reduces the severity of spurious errors when they occur. Across all settings, $\mathrm{AvgExtraLabels}$ is consistently lower for COMPASS-PTM (around 1.04–1.11) than for SAGEPhos (around 1.30–1.76), meaning that SAGEPhos more often produces cascades of multiple extra PTM types at the same residue, whereas COMPASS-PTM's spurious errors are typically limited to a single additional label. This difference is important because excessive multi-label over-calling can inflate apparent residue-level co-occurrence and distort protein-level PTM patterns.

Third, the single-label subset ($|Y| = 1$) provides an especially unambiguous coherence diagnostic. When the curated annotation indicates exactly one PTM type at a residue, additional predicted PTM types are particularly likely to be unsupported. COMPASS-PTM maintains both low spurious rates and low $\mathrm{AvgExtraLabels}$ in this subset, supporting that the model does not rely on over-expanded multi-label predictions to achieve residue-wise recall.

Finally, we complement these residue-set coherence analyses with a protein-level event-F1 (macro over proteins), which evaluates each protein as a structured set of residue–PTM events. On the test set, COMPASS-PTM achieves a mean protein-level F1 of 0.6430, indicating that the predicted PTM event composition across each protein is substantially aligned with the ground-truth program, rather than being accurate only in an averaged residue-wise sense.

Comment 3.3: *3. ESPS relies on a fixed 15-mer window around candidate sites, which is empirically justified via ablations, but this design choice has important biological implications. Many kinase and ligase recognition events depend on distal docking motifs, intrinsically disordered regions outside short windows, or domain-level context. It would be great if the authors evaluated sensitivity to window placement and length in a more targeted way, or tested whether incorporating longer-range substrate context improves cold-start enzyme generalization. At minimum, a clearer discussion of what classes of enzyme–substrate interactions this formulation can and cannot capture would help calibrate expectations.*

Response 3.3: We thank the reviewer for raising this important point. We agree that using a fixed site-centered 15-mer window is not only a modeling choice but also an implicit biological assumption about the determinants of enzyme–substrate recognition, and that many interactions can involve distal or broader context beyond a short local window. In ESPS, we encode enzymes using full-length sequence representations, while summarizing substrates by a site-centered peptide; accordingly, ESPS is intended to capture a predominantly site-centric regime where local sequence determinants are informative, and it should not be interpreted as a complete mechanistic model of docking- or context-dependent recognition.

(i) Testing longer-range substrate context via window length on kinase enzyme cold-start. Following the reviewer's suggestion, we evaluated whether incorporating longer-range substrate context improves enzyme generalization by varying the substrate window length while keeping the ESPS architecture, enzyme encoder, training protocol, and evaluation split fixed. As shown in Table F, extending the site-centered substrate context from 15aa to 21aa and 51aa yields modest gains on several ranking- and balance-sensitive metrics (e.g., F1/AUROC/AUPRC), with 51aa achieving the strongest overall values among the tested lengths (F1 0.7283; AUROC 0.7759; AUPRC 0.7636), while accuracy remains comparable across settings. These results are aligned with the reviewer's hypothesis that broader substrate context can matter for enzyme generalization: under the enzyme cold-start split, expanding the site-centered window from 15aa to 21aa and 51aa yields a small but consistent improvement in F1/AUROC/AUPRC, with 51aa performing best overall. This pattern suggests that, beyond the immediate 15-mer motif core, additional flanking residues can contribute complementary sequence cues that help distinguish enzyme-specific preferences in a harder generalization regime. The gains, however, remain modest. A likely reason is that the supervision in this benchmark is still residue- or site-centered, rather than explicitly labeling which distal segments mediate docking or long-range recognition. As a result, the extra context may offer only weak auxiliary cues, instead of a directly supervised signal for distal regulatory determinants.

(ii) A direct test of incorporating full-length substrate context (baseline). To further test a longer-range context formulation beyond simply enlarging the local window, we implemented a minimal ESPS variant in which the local 15-mer representation attends to a full-length substrate representation via a single cross-attention layer with a residual connection:

$$\mathbf{H}^{\text{local}} \leftarrow \text{LayerNorm}\Big(\mathbf{H}^{\text{local}} + \text{Dropout}(\text{Attn}(\mathbf{Q} = \mathbf{H}^{\text{local}}, \mathbf{K} = \mathbf{H}^{\text{global}}, \mathbf{V} = \mathbf{H}^{\text{global}}))\Big).$$

Empirically, this full-length fusion baseline performs comparably to the local-window models but does not yield a clear, consistent improvement across metrics (Table F). In particular, its AUROC and ACC are similar to those of the local 15-mer-window variants, whereas its AUPRC is lower. We interpret this conservatively: while distal docking and domain-/scaffold-level determinants are biologically important for certain enzyme families, the curated enzyme–substrate supervision does not specify which distal segments mediate recognition. As a result, a straightforward full-length fusion may mix informative and non-informative regions and may not reliably translate distal context into improved generalization without more targeted long-range modeling or finer-grained supervision.

Setting	ACC	F1	AUROC	AUPRC
15aa (default)	**0.7068**	0.7123	0.7696	0.7498
21aa	0.7034	0.7247	0.7726	0.7529
51aa	0.7037	**0.7283**	**0.7759**	**0.7636**
15aa + global substrate cross-attn (baseline)	0.7066	0.7242	0.7693	0.7382

TABLE F: **Testing longer-range substrate context on kinase enzyme cold-start.** We vary substrate window length (15/21/51aa) and additionally test a minimal full-length substrate fusion via cross-attention (15aa + global).

(iii) Clarifying what ESPS can and cannot capture (revision to Discussion). We will revise the Discussion to calibrate scope explicitly: (a) Better captured: interactions that are primarily governed by the proximal sequence context around the modified residue. (b) Not fully captured: cases where specificity critically depends on distal docking motifs, multi-domain substrate context, scaffold/adaptor effects, or 3D proximity, which are not explicitly represented in the current ESPS formulation.

Manuscript Discussion

It does not explicitly account for higher-order structural features, cellular context, or temporal dynamics of signaling, all of which are known to modulate PTM accessibility and enzyme specificity. **In addition, ESPS represents substrates using a site-centered 15-mer, which is well-suited for capturing proximal recognition motifs but cannot explicitly model distal docking interactions or long-range domain/scaffold effects.**

Comment 3.4: *4. I think the paper would benefit from a much more explicit comparison to PTM-Mamba (in Nature Methods, the authors may have incorrectly cited this as from a conference), which also proposes a PTM-aware sequence representation but from a fundamentally different angle. PTM-Mamba explicitly tokenizes modified residues and learns bidirectional sequence representations over wild-type and PTM-modified proteoforms using Mamba blocks, whereas COMPASS-PTM operates entirely on wild-type sequences and encodes PTM logic implicitly through multi-label supervision, co-occurrence prompting, and downstream enzyme pairing. Right now, this distinction is not clearly articulated, and it is easy for readers to conflate the two approaches. It would be great if the authors explicitly stated what information their representation can capture that PTM-Mamba cannot (for example, enzyme-specific rewiring from wild-type sequence alone), and conversely what they give up by not modeling explicit PTM tokens or modified proteoforms. A small experiment comparing MSPN embeddings to PTM-Mamba embeddings on a shared downstream task, or at least a conceptual diagram contrasting "implicit PTM programs" versus "explicit PTM tokenization," would go a long way for me to see the novelty and scope of the contribution.*

Response 3.4: We thank the reviewer for highlighting **PTM-Mamba** as a closely related and influential study that advances PTM-aware sequence representation through explicit PTM tokenization and proteoform-level modeling. We agree that the current manuscript does not distinguish PTM-Mamba and COMPASS-PTM clearly enough, which may lead readers to conflate these two complementary frameworks. In the revision, we will therefore add an explicit side-by-side comparison that clarifies (i) the conceptual differences and trade-offs between PTM-Mamba and COMPASS-PTM, (ii) a new head-to-head evaluation on a shared ESPS task under identical protocols.

(1) Conceptual distinction between PTM-Mamba and COMPASS-PTM (explicit proteoforms vs. implicit regulatory programs).

PTM-Mamba learns a PTM-aware language model by explicitly tokenizing modified residues and training on PTM-modified sequences (proteoforms) with bidirectional gated Mamba blocks (optionally fused with ESM-2 features), thereby encoding the identity and position of PTMs directly in the input representation; this design is particularly suitable for settings where proteoform context itself is central (e.g., PTM-conditioned functional readouts such as disease/druggability prediction over modified sequences, PTM effects on PPIs, or masked-position PTM discovery). In contrast, COMPASS-PTM targets a different regime: starting from wild-type sequence alone, it learns a coarse-to-fine, mechanism-aware representation that first predicts a multi-label PTM site profile as an implicit PTM program, and then refines this program into enzyme–substrate pairing (writer/eraser assignment) through a shared substrate representation; mechanistically, PTM logic is captured implicitly via multi-label supervision, crosstalk-aware prompting (injecting PTM co-occurrence priors as attention bias), and downstream enzyme pairing that forces the representation to be aligned with catalytic specificity. As a result, COMPASS-PTM is explicitly optimized to capture enzyme-specific rewiring and regulatory hypotheses from wild-type sequence alone and to encourage program-level coherence under learned PTM-dependency priors, whereas it gives up direct conditioning on a specific proteoform state and does not aim to model proteoform-conditioned consequences in the same explicit manner as PTM-Mamba.

(2) New empirical comparison on a shared downstream task (ESPS).

Following the reviewer's suggestion, we performed a head-to-head comparison between PTM-Mamba and COMPASS-PTM on the shared enzyme–substrate pairing task (ESPS, OmniPath). To ensure a fair and controlled comparison, we kept the entire ESPS pipeline identical to COMPASS-PTM and only replaced the substrate encoder with PTM-Mamba. Concretely, the pairing head architecture, loss, training schedule, hyperparameters, negative sampling strategy, and data splits are all the same as in COMPASS-PTM; the only change is the source of substrate embeddings (PTM-Mamba vs. MSPN). The results are summarized in Table G and added to the updated main text Fig. 3 (shown in Fig. D).

TABLE G: Performance Comparison between PTM-Mamba and COMPASS-PTM

Dataset	Method	ACC (%)	F1-Score (%)	AUC (%)	AUPRC (%)
OmniPath Warm-start	PTM-Mamba	73.77 ± 0.29	75.43 ± 0.31	81.10 ± 0.31	78.19 ± 0.26
	COMPASS-PTM	$\mathbf{79.28 \pm 0.14}$	$\mathbf{80.11 \pm 0.23}$	$\mathbf{86.55 \pm 0.13}$	$\mathbf{84.55 \pm 0.12}$
OmniPath Sub Cold-start	PTM-Mamba	72.01 ± 0.46	73.04 ± 0.09	79.43 ± 0.38	76.99 ± 0.57
	COMPASS-PTM	$\mathbf{76.89 \pm 0.07}$	$\mathbf{77.99 \pm 0.83}$	$\mathbf{84.85 \pm 0.03}$	$\mathbf{83.02 \pm 0.21}$
OmniPath Enz Cold-start	PTM-Mamba	65.44 ± 0.61	67.92 ± 0.89	71.34 ± 0.54	68.31 ± 0.63
	COMPASS-PTM	$\mathbf{70.68 \pm 0.44}$	$\mathbf{71.23 \pm 0.42}$	$\mathbf{76.96 \pm 0.35}$	$\mathbf{74.98 \pm 0.27}$

Across OmniPath ESPS warm-start and both cold-start regimes, COMPASS-PTM shows a consistent improvement over PTM-Mamba. Specifically, we observe about 4.9–5.5% absolute gains in ACC, 3.3–5.0% in F1, 5.4–5.6% in AUC, and 6.0–6.7% in AUPRC, and this advantage persists under both substrate- and enzyme-cold-start splits. These results indicate that learning an implicit, crosstalk-aware PTM program from wild-type sequences yields a substrate representation that transfers more effectively to enzyme–substrate pairing. Stage 1 is trained to jointly model multi-label PTM site patterns and to absorb PTM co-occurrence structure through prompting, encouraging the representation to organize residues and sequence context around biochemically plausible modification programs rather than explicit proteoform token identities. This program-level structure provides a stronger substrate-side signal for the downstream ESPS head and remains robust when generalization is stressed by cold-start settings. At the same time, PTM-Mamba is particularly well-suited for proteoform-conditioned scenarios

where explicit PTM tokenization is essential; our ESPS comparison primarily highlights that COMPASS-PTM's program-centric training objective better matches enzyme-centric generalization from wild-type sequences.

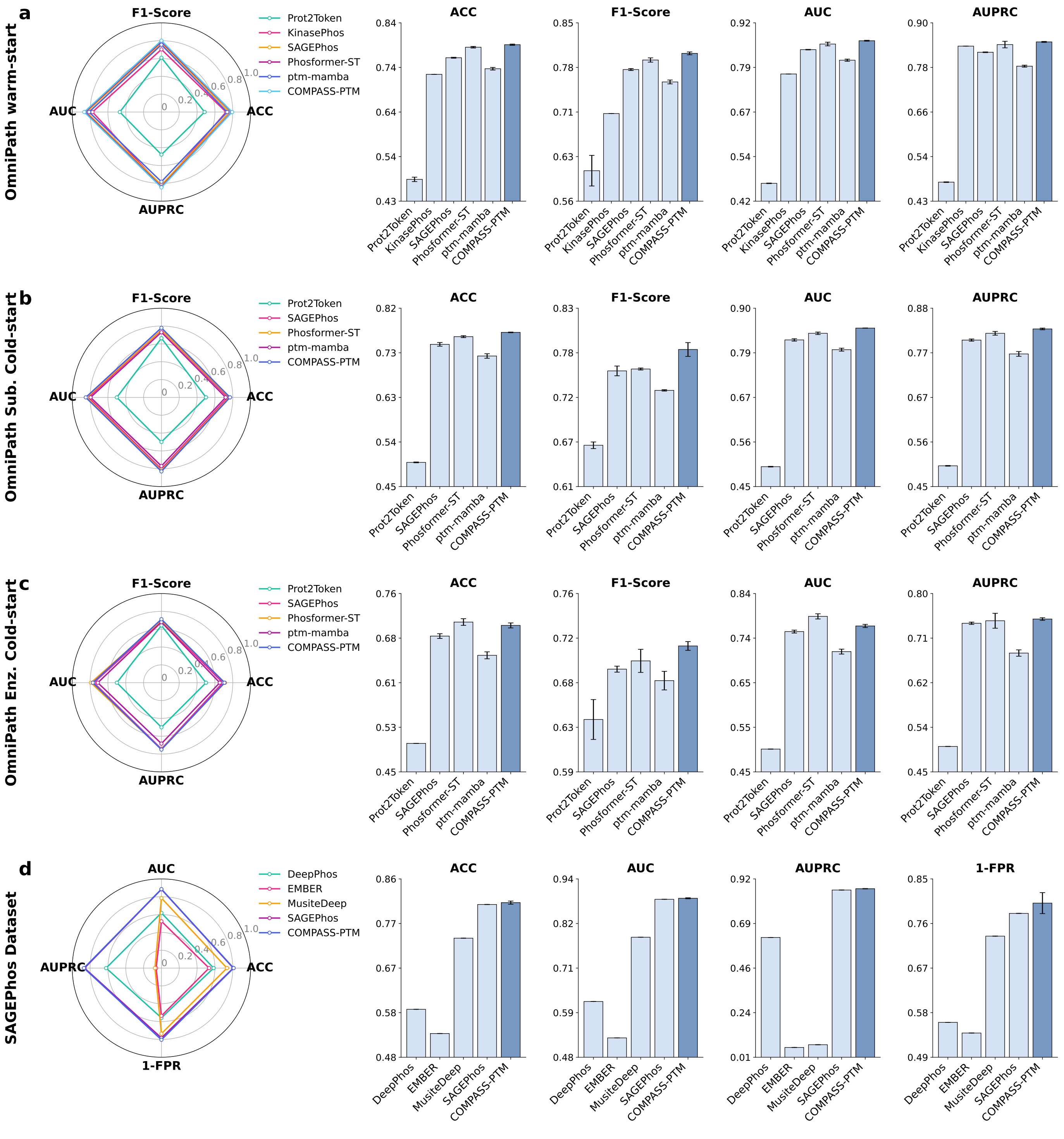

Figure D: **ESPS demonstrates robust and high-fidelity performance in enzyme-substrate prediction across diverse benchmarks.**

(3) Manuscript changes for clarity (implemented in revision). To address the reviewer's request on presentation, we have made the following concrete changes:

- **Main Text Fig. 3 updated:** include PTM-Mamba as an additional baseline on OmniPath benchmarks with identical evaluation splits and reporting (Tab. G).
- **New discussion paragraph:** explicitly clarify the conceptual and practical differences between COMPASS-PTM and PTM-Mamba (implicit PTM program learning from wild-type sequences with crosstalk prompting and downstream enzyme

pairing vs. explicit PTM tokenization over PTM-modified proteoforms), so readers do not conflate the two approaches.
- **Citation correction:** we have corrected the PTM-Mamba reference in the manuscript to properly cite the Nature Methods journal article (instead of the earlier conference-style citation), as the reviewer noted.

Manuscript Results Section 2.2.3

On the OmniPath benchmark, we re-trained all relevant baselines on OmniPath under the same protocol to ensure a fair comparison, keeping each baseline's original architecture unchanged. The only exception is **PTM-Mamba**: rather than re-implementing an alternative ESPS pipeline, we performed a controlled substitution by replacing the MSPN substrate encoder in our ESPS framework with PTM-Mamba while keeping all other ESPS components identical. We include a detailed discussion of PTM-Mamba and its relationship to COMPASS-PTM in Supplementary Information S.1.4.

Manuscript Supplementart Information S.1.4

PTM-Mamba (ESPS comparison baseline).
Beyond residue-level PTM site prediction, our study also evaluates **enzyme–substrate pairing** (ESPS) on OmniPath (Stage 2). Since ESPS relies on a substrate sequence representation as input to the pairing model, we additionally compare against **PTM-Mamba**, a recent work that learns PTM-aware sequence embeddings via explicit proteoform tokenization. In PTM-Mamba, modified residues are treated as distinct tokens and the model is trained over PTM-modified proteoforms, producing embeddings that explicitly reflect the presence and type of modifications. In contrast, COMPASS-PTM operates on wild-type sequences only: Stage 1 learns an **implicit, multi-label PTM program** with crosstalk-aware prompting, and Stage 2 uses this program-oriented substrate representation for downstream ESPS. To compare representations without conflating differences in downstream architecture, we use a controlled substitution strategy on OmniPath benchmarks: we keep the entire ESPS pipeline identical to COMPASS-PTM and only replace the substrate encoder (MSPN) with PTM-Mamba. All other components (pairing head, loss, optimizer, training schedule, and data splits and so on) remain unchanged.

Comment 3.5: *Minor Points: 1. Fig. 2 radar plots are hard to read because absolute values are not labeled. I think adding numeric values directly to the bars or including a small table would improve interpretability.*

Response 3.5: We thank the reviewer for this helpful suggestion. We agree that the original radar plots in Fig. 2 could be difficult to interpret without explicit absolute values. In the revised manuscript, we have added numeric values directly on the radar plots at each vertex to make the exact performance readily readable, while keeping the visualization uncluttered, as shown in Fig. E.

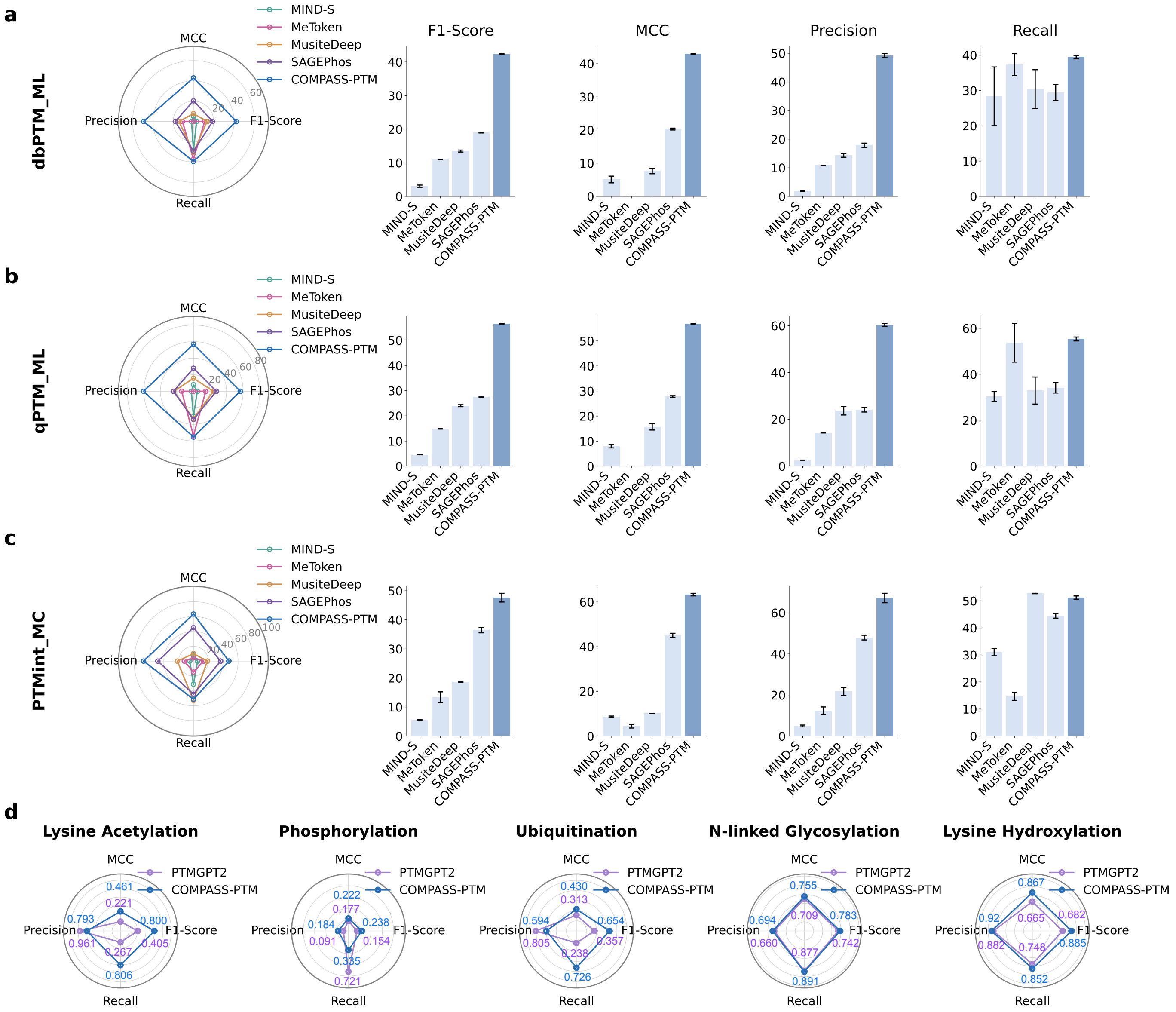

Figure E: **MSPN demonstrates state-of-the-art performance and robust generalization across benchmark datasets.**

Comment 3.6: *2. UMAP and PCA figures (Fig. 4) reuse similar color palettes across panels, but the legend ordering changes. This makes it unnecessarily hard to track PTM types and kinase families across subfigures.*

Response 3.6: We agree that tracking categories across panels can be hindered when a single legend serves multiple subfigures. In the revised Main Text Fig. 4 (Fig. F), we reorganized the legends by semantic groups and placed them adjacent to the corresponding panels (single-PTM, multi-PTM, and kinase-family), while keeping a fixed and consistent category order within each legend. This reduces cross-panel lookup and improves readability.

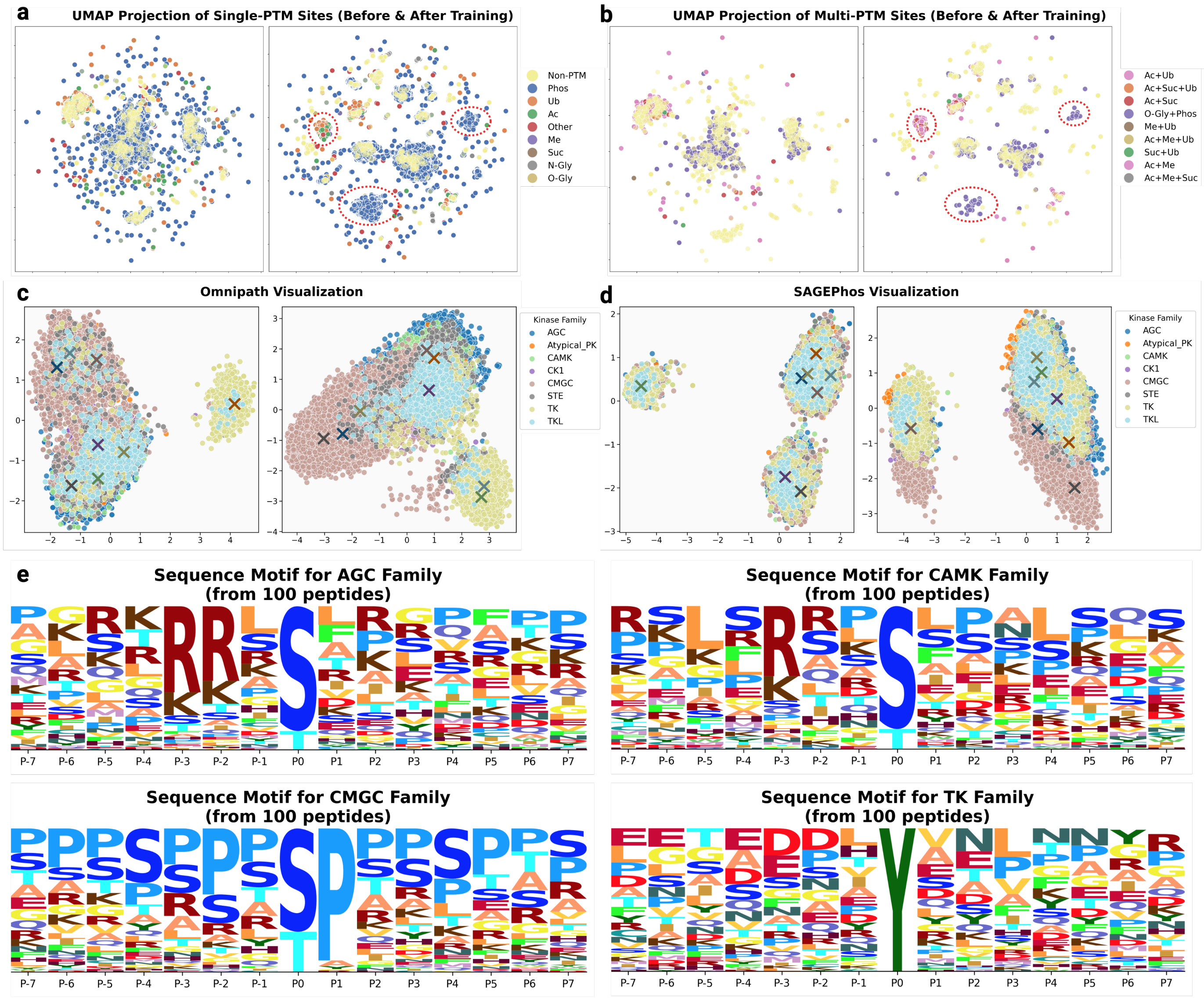

Figure F: **Visualizing the learned representations of the COMPASS-PTM framework.**

Comment 3.7: *3. In Fig. 5 and Fig. 6, the probability values are shown with inconsistent precision (sometimes three decimals, sometimes four). Standardizing this would clean up the presentation.*

Response 3.7: We thank the reviewer for noting this presentation issue. We agree that the probability annotations in Fig. 5 and Fig. 6 previously used inconsistent numeric precision. To improve readability and ensure a consistent visual standard, we have revised the figure annotations and standardized the probability values in Fig. 6 to three decimal places throughout, as shown in Fig. G.

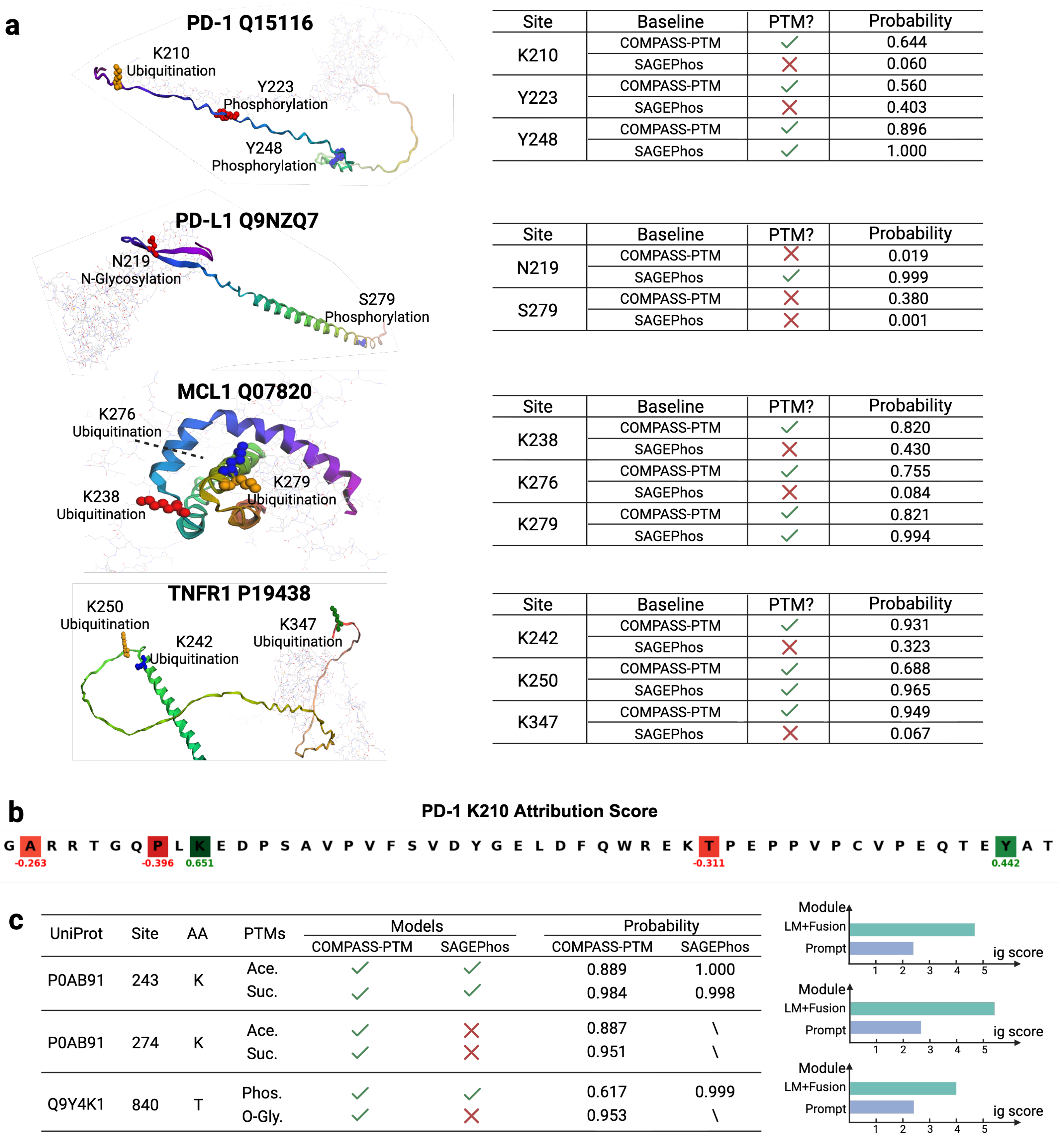

Site	Baseline	PTM?	Probability
K210	COMPASS-PTM	✓	0.644
	SAGEPhos	✗	0.060
Y223	COMPASS-PTM	✓	0.560
	SAGEPhos	✗	0.403
Y248	COMPASS-PTM	✓	0.896
	SAGEPhos	✓	1.000

Site	Baseline	PTM?	Probability
N219	COMPASS-PTM	✗	0.019
	SAGEPhos	✓	0.999
S279	COMPASS-PTM	✗	0.380
	SAGEPhos	✗	0.001

Site	Baseline	PTM?	Probability
K238	COMPASS-PTM	✓	0.820
	SAGEPhos	✗	0.430
K276	COMPASS-PTM	✓	0.755
	SAGEPhos	✗	0.084
K279	COMPASS-PTM	✓	0.821
	SAGEPhos	✓	0.994

Site	Baseline	PTM?	Probability
K242	COMPASS-PTM	✓	0.931
	SAGEPhos	✗	0.323
K250	COMPASS-PTM	✓	0.688
	SAGEPhos	✓	0.965
K347	COMPASS-PTM	✓	0.949
	SAGEPhos	✗	0.067

UniProt	Site	AA	PTMs	Models		Probability	
				COMPASS-PTM	SAGEPhos	COMPASS-PTM	SAGEPhos
P0AB91	243	K	Ace.	✓	✓	0.889	1.000
			Suc.	✓	✓	0.984	0.998
P0AB91	274	K	Ace.	✓	✗	0.887	\
			Suc.	✓	✗	0.951	\
Q9Y4K1	840	T	Phos.	✓	✓	0.617	0.999
			O-Gly.	✓	✗	0.953	\

Figure G: **Case studies demonstrating the accuracy and mechanistic interpretability of COMPASS-PTM on clinically relevant targets.**

Comment 3.8: *4. The term "dual long-tail distribution" is introduced multiple times in the Introduction and Results. It would read better if it were defined once and then referenced consistently.*

Response 3.8: We thank the reviewer for this helpful editorial suggestion. We agree that repeatedly introducing the term "dual long-tail distribution" without a single fixed definition can reduce readability. In the revised manuscript, we now define **"dual long-tail distribution"** only once, at its first occurrence in the Introduction, and we use the term consistently thereafter without re-defining it in later sections. Specifically, by "dual long-tail distribution" we mean two coupled sources of imbalance in PTM learning: **(i) a long-tailed distribution over PTM types**, where a few common modifications contribute the majority of labeled examples while many biologically important PTMs are rare; and **(ii) a long-tailed distribution over sequence positions**, where true modified sites are extremely sparse relative to the overwhelming background of unmodified residues. Together, these two effects induce pronounced imbalance at both the class and instance levels.

Changes in manuscript: We added this explicit definition at the first mention of "dual long-tail distribution" in the Introduction and removed the later redundant re-definitions in the Results and related sections, so that subsequent occurrences now refer back to the same definition with consistent wording.

Manuscript Introduction

However, three fundamental challenges remain. **First**, most methods lack an explicit mechanism to encode biochemical rules of crosstalk—such as when phosphorylation facilitates ubiquitination, or acetylation competes with methylation on lysine—so they struggle in co-modified contexts where conditional dependence is central to function. **Second, PTM data exhibit a dual long-tail distribution: (i) across PTM types, a small number of common modifications account for most labeled instances, whereas many biologically important PTMs are rare; and (ii) across sequence positions, positive modification sites are extremely sparse relative to the overwhelming background of unmodified residues. Together, these two coupled long-tail effects induce severe imbalance at both the class and instance levels.**

Comment 3.9: *5. Several Supplementary figure references (e.g., S.2.x) appear before the figures are described conceptually in the main text.*

Response 3.9: We thank the reviewer for pointing this out. We have revised the main text to ensure that Supplementary figure/table references are introduced after the corresponding concept has been described in the narrative, so readers understand what each Supplementary item is intended to support before being directed to it. For example, in Results Section 2.2.1 we now first introduce that Supplementary Fig. 1 summarizes key benchmark dataset statistics and label-distribution properties, and only then cite the relevant panels.

Manuscript Results Section 2.2.1

Across these benchmarks, proteins are processed into PTM-site–anchored segments ($\leq$ 50 aa) and split at the protein level via MMseqs2 clustering (40% identity) to minimize redundancy and evolutionary leakage, detailed specifications for each dataset are provided in the Supplementary Information S.1.1.2. **And Supplementary Fig. 1 summarizes key dataset statistics for our benchmarks, including PTM-type frequency and site-level sparsity/imbalance, as well as sequence-level distributions.** Critically, all three datasets exhibit the dual long-tail distributions characteristic of real-world proteomic data (Supplementary Fig. 1**b**,**e**,**h**,**j**), providing a stringent and realistic testbed for evaluating proteome-scale prediction models.
We assessed model performance using a suite of metrics, primarily F1-score and the Matthews Correlation Coefficient (MCC), two metrics renowned for their reliability in evaluating classifiers on imbalanced multi-label data. To rigorously evaluate performance across both common and rare PTMs, all metrics were calculated using a macro-averaging approach that prevents class imbalance from skewing the results. **Detailed description of these metrics are shown in Supplementary Information S.1.2.**

Comment 3.10: *6. Fig. 1b's crosstalk-aware prompting module uses arrows and labels that are too small at print resolution. I think increasing font size or simplifying the diagram would help.*

Response 3.10: We thank the reviewer for this helpful suggestion. We agree that the arrows and text labels in the crosstalk-aware prompting pathway of Fig. 1b (e.g., the PTM-relation prior feeding into the prompt and prediction modules, as well as the small arrow annotations within the MSPN block) can become difficult to read at print resolution. In the revised manuscript, we have increased the font size and line weights for the relevant arrows/labels and simplified the visual annotations to improve legibility while preserving the intended workflow.

Changes in manuscript: We updated Fig. 1b by enlarging the fonts and arrow annotations in the crosstalk-aware prompting module and streamlining the labels to ensure readability in the final printed layout, as shown in Fig. H.

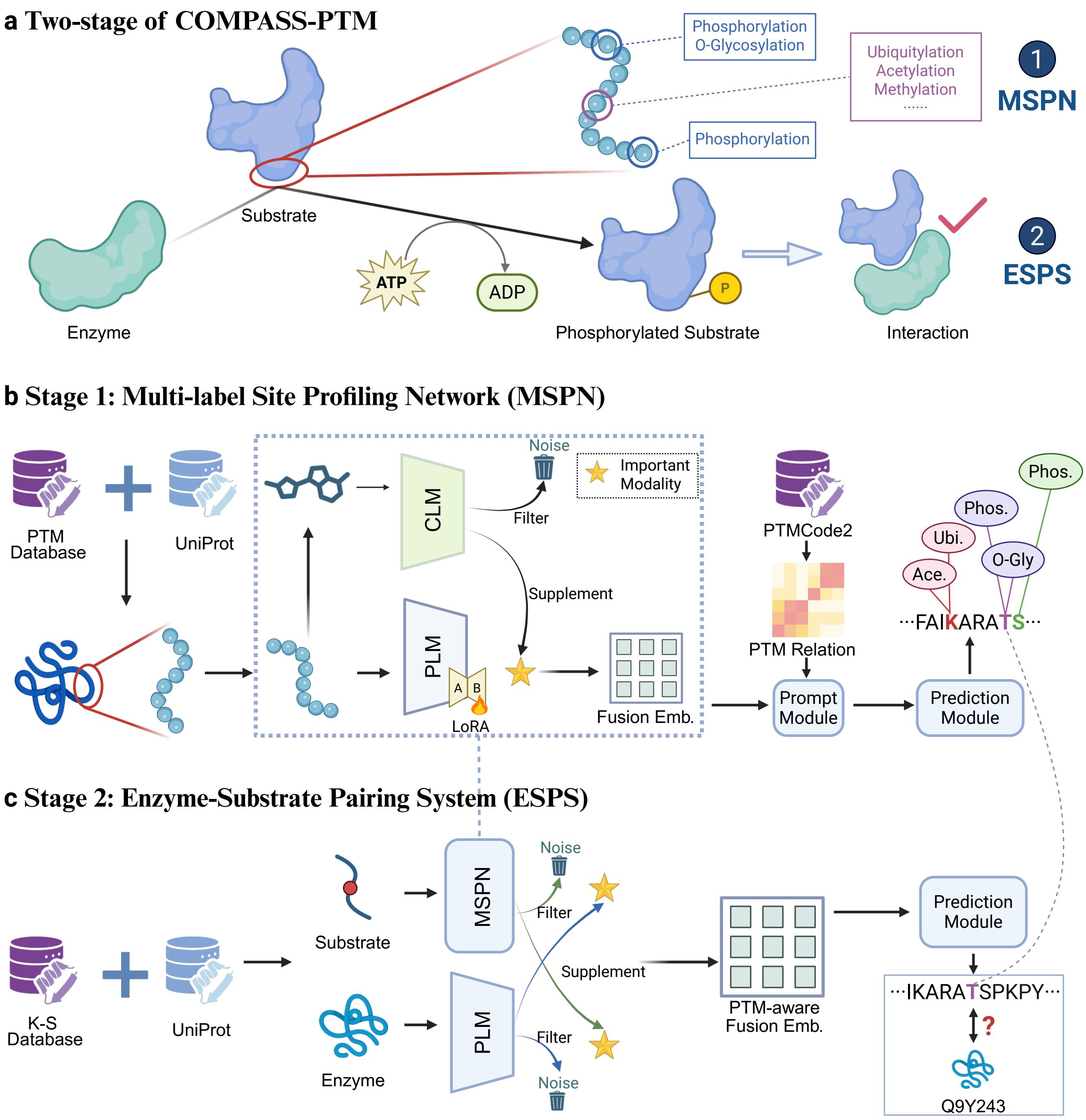

Figure H: **The COMPASS-PTM framework for mechanism-aware PTM prediction.**

Editorial note: figure Created in BioRender. Cao, H. (2026) https://BioRender.com/ax2kzid

Comment 3.11: *7. In the Methods, equations (3) and (4) introduce α twice with different meanings (scaling vs loss weighting). Renaming one would avoid confusion.*

Response 3.11: We thank the reviewer for pointing out this notation ambiguity. We agree that the symbol (α) was previously used to denote two different concepts in the Methods: (i) the learnable scaling factor applied to the crosstalk interaction term in Eq. (3), and (ii) the learnable fusion coefficients controlling the gated/residual components in the PLM–CLM fusion module. To avoid confusion, in the revised manuscript we have renamed the scaling parameter in Eq. (3) from (α) to (γ), while retaining (α) and (β) for the fusion coefficients. This change is purely notational and does not affect the model or any results.

Changes in manuscript: In Eq. (3) and the associated text, we replaced (α) with a learnable parameter (γ).

Manuscript Methods Section 4.2.3

Using these probabilities, we dynamically compute a site-pair-specific attention prompt, $\mathbf{B}$. The prompt value between any two positions, i and j, quantifies the biochemical plausibility of their co-modification, mediated by the learned relationship matrix $\mathbf{R}$. This interaction term is passed through a 'tanh' activation, regularized with 'Dropout', and scaled by **a learnable parameter** γ:

$$\mathbf{B}_{ij} = \text{Dropout}(\tanh(\mathbf{p}_i^T \mathbf{R} \mathbf{p}_j)) \cdot \gamma$$
